# Supplementary material for: Successive remodeling of IgG glycans using a solid-phase enzymatic platform
Source: Commun Biol. 2022 Apr 7;5:328. doi: 10.1038/s42003-022-03257-4 (PMC8990068; doi:10.1038/s42003-022-03257-4)
Supplement: Supplementary file 2 — Supplementary Information [file 42003_2022_3257_MOESM2_ESM.pdf]

Supplementary Information for

Successive remodeling of IgG glycans using a solid-phase enzymatic platform

Yen-Pang Hsu, Deeptak Verma, Shuwen Sun, Caroline McGregor, Ian Mangion, Benjamin F. Mann*

*Corresponding author.

Correspondence should be addressed to ben.mann@gmail.com

Raw data that support the findings of this study are available upon request.

Table of content

Supplementary Methods………………………………………………………………………………………………… 2

Supplementary Notes………………………………………………………………………………………………… 2

Supplementary Tables………………………………………………………………………………………………… 4

Supplementary Figures………………………………………………………………………………………………… 8

Supplementary References………………………………………………………………………………………… 25

Supplementary Methods

Human serum IgG (I4506), Tris-HCl (T-5941), HEPES (H4034), Sodium acetate (S2889), Calcium chloride (C5670), Magnesium chloride (M4880), Manganese Chloride solution (M1787), Sodium chloride solution (S5150), CMP-NANA (C8271), UDP-Gal (U4500), UDP-GlcNAc (U4375), GDP-Fucose (G4401), Acetonitrile (900667), Discovery glycan SPE columns (55465-U), and empty SPE column and frit (57607-U) are purchased from Sigma-Aldrich. Protein A resin (53139), Protein A-IgG binding buffer (54200), Protein A-IgG elution buffer (21027), H_2O (10977015) were purchased from ThermoFisher. HILIC columns for chromatography (186004742), SPE µPlate (186002780), µPlate extraction manifold (186001831), SPE vacuum manifold (WAT200607), RapiGest SF (186008090), Glycoworks buffer (186008100), glycan quantitative standard (186008791), and Rapifluor-MS (186008091) were purchased from Waters. MWCO filters (UFC503096) were purchased from Millipore Sigma. Rapid PNGaseF (P0710) was purchased from NEB. Human FcR AlphaLISA Binding Kit (AL3081C) was purchased from PerkinElmer. Please refer to **Table S1** for the vendor information of glycoengineering enzymes. No unexpected or unusually high safety hazards were encountered in this work.

Chromatography and mass spectrometry analyses were conducted using an Agilent 1290 Infinity II LC system tandem with Agilent 6500 Series quadrupole time-of-flight MS system. Enzyme concentration was determined by absorbance at 280 nm using NanoDrop 2000 (Thermo Scientific). Temperature-controlled reactions/incubations were performed in ThermoFisher MaxQ 6000 incubator and Fisherbrand Thermal Mizer II. Measurements of hydrodynamic diameter, melting temperature (T_m) and aggregation temperature (T_{agg}) were performed on Uncle (all-in-one biologics stability screening platform) from Unchained Labs. The detection of AlphaLISA-based assays for IgG-FcγR binding studies was conducted by EnVision Multimode Plate Reader 2105 (PerkinElmer).

Supplementary Notes

1. Comparison between substrate immobilization and enzyme immobilization in SPGR

An alternative approach to conduct SPGR is immobilizing the glycoengineering enzymes on solid supports instead of the IgG substrates **(Figure S15)**.[1,2] In this way, glycoengineering enzymes can be easily pulled out from the reaction pools for recovery. This could improve the efficiency of large-scale productions where the enzymes are re-charged in multiple batches of reactions. Furthermore, because substrates present the majority of the substance in the reactions, immobilizing the enzymes, instead of the substrate, could reduce the cost of immobilization in the production pipelines. However, there are also restrictions in this manner. For example, the selection of enzymes for the remodeling processes is much more limited in this case because the buffer of the IgG solution can not be swapped easily. In a remodeling process involving both glycosidases and glycosyltransferases—which prefer very different conditions (pH, cations)— the use of a generic working buffer would significantly compromise the enzyme activities. In addition, the preparation of resin-immobilized glycoengineering enzymes could be difficult. Whether immobilization affects their activity and substrate selectivity, as well as the immobilization protocol itself, remains to be investigated. Together, for the glycan remodeling that involves multiple glycoengineering enzymes, we believe the substrate-immobilization SPGR (this study) is preferred.

2. Endoglycosidases for glycan remodeling

IgG glycan trimming can also be implemented through the chitobiose core GlcNAc residues. Endoglycosidases specifically cleave the β1-4 linkage between the GlcNAc residues in the chitobiose core, shaving off the majority of glycans [3-6]. This makes them powerful tools when the removal of IgG glycan is needed. They have also been

employed for chemoenzymatic glycan modification where native glycans of glycoproteins are removed by endoglycosidases, followed by synthetic glycan installation back to the proteins using mutated endoglycosidases (also known as glycosynthases).[7] To know whether endoglycosidases can be applied to SPGR, we analyzed the activity of endoglycosidases on intact IgG. Of the six tested enzymes, the candidate from *Streptococcus pyogenes* (known as Endo S) exhibited the highest conversion ratio on intact IgG (CR_{50}=1:0.007, **Figure S16**). It has been known that endoglycosidases have different preferences for substrate structures. In agreement with reported studies, Endo S effectively liberates N-glycans from human IgG in SPGR.[4] On the other hand, the ones with higher specificity to high-mannose glycans, such as endoglycosidase D, did not show detectable IgG glycan conversion in our screening (**Table S1**).[8] A complete Endo S reaction led to the removal of glycan majority, giving a relatively clean chromatogram as shown in **Figure S16**.

3. *Candidatus omnitrophica* fucosidase has a wide spectrum on substrate selectivity

With the use of high enzyme concentration and long incubation time, we found that fucosidase from *C. omnitrophica* functioned on all the IgG glycoforms. **(Figure S4c)** While we also observed decreased activity as the structural complexity of the glycans increased **(Figure S4d)**, the broad spectrum of substrate selectivity makes this enzyme an attractive tool for glycan remodeling on intact IgG. Opportunities to improve its activity through genetic engineering is worth to be investigated.

4. Steric hindrance created by IgG immobilization reduces glycoengineering enzyme activities

IgG immobilization enables efficient washing and reaction-swapping processes in SPGR. However, we observed reduced enzyme activities on immobilized IgG compared to non-immobilized, free IgG **(Figure S17a)**. Such a "trade-off" partially results from the relatively limited surface area in heterogeneous reactions but could mainly be attributed to the increased steric hindrance created by IgG binding to protein A. It's reported that protein A binds to the IgG Fc at the interface between the CH2 and CH3 domains.[9] This binding region is not only close to but also interacting with the Asn297 glycans.[10] Computational modeling of interactions between the full-length protein A (with four domains) and the Fc region indicates steric hindrance, specifically at the CH2 region **(Figure S17b-c)**. This could lead to reduced accessibility of the glycans by glycoengineering enzymes. This hypothesis of spatial hindrance and reduced accessibility is supported by the size effect of glycoengineering enzymes whose reduced activity correlates inversely with their molecular weight. For example, the largest enzyme in our toolset, Gal from *S. pneumoniae* (231kD), showed an activity reduction of 74% when functioning on immobilized IgG. A significant activity reduction was also found in the fucosidase reactions despite the smaller size of the enzyme (49kD) whose chemistry takes place at the reducing end of the glycan structure. In addition to steric hindrance, multiple IgG Fc regions could be interacting with the same protein A molecule, thus leading to a crowding effect and reduced enzymatic activity.

Supplementary Tables

	Enzyme	Source	Size (kD)	Targets (glycans with)	Conversion Ratio (%) 1 hour	Conversion Ratio (%) 24 hours	Vender & Cat #
Exoglycosidases							
V	Neuraminidase	*C. perfringens*	43	Terminal sialic acid	66.4 ± 4.7	90.2 ± 1.3	NEB P0720
	Neuraminidase A	*A. ureafaciens*	100	Terminal sialic acid	55 ± 3.5	56.5 ± 1.2	NEB P0722
	Neuraminidase S	*S. pneumoniae*	74	Terminal sialic acid	N.D.	9.6 ± 1.6	NEB P0743
V	Galactosidase S	*S. pneumoniae*	231	Terminal galactose	5.5 ± 2.2	51 ± 8.1	NEB P0745
	Galactosidase	*B. taurus* (testis)	71	Terminal galactose	3.5 ± 0.9	3.7 ± 1.6	NEB P0746
	Galactosidase 1	*H. sapiens*	74	Terminal galactose	N.D.	3 ± 0.7	R&D Systems 6464-GH
V	N-Acetylglucosaminidase S	*S. pneumoniae*	125	Terminal GlcNAc	47 ± 1	82.9 ± 1.8	NEB P0744
	N-Acetylhexosaminidase F	*S. picatus*	100	Terminal GlcNAc	N.D.	N.D.	NEB P0721
	Fucosidase	*B. Taurus* (kidney)	52	Terminal Fucose	N.D.	1.2 ± 0.9	NEB P0748
V	Fucosidase O	*C. Omnitrophica*	49	Terminal Fucose	1.3 ± 0.2	2.4 ± 1	NEB P0749
	Fucosidase	*Prunus dulcis*	56	Terminal Fucose	N.D.	N.D.	NEB P0769
	Fucosidase	*C. meningosepticum*	50	Terminal Fucose	N.D.	N.D.	Sigma 344826
	Fucosidase	*E. miricola*	56	Terminal Fucose	N.D.	N.D.	Sigma F6272
	Fucosidase	*H. sapiens*	51	Terminal Fucose	N.D.	N.D.	R&D Systems 7039-GH
	Fucosidase	*T. maritima*	54	Terminal Fucose	N.D.	N.D.	R&D Systems 6556-GH
Endoglycosidases							
	Endoglycosidase A	*A. protophormia*	69	GlcNAc-GlcNAc linkage	N.D.	N.D.	Chemily G. EN01017
	Endoglycosidase D	*S. pneumoniae*	140	GlcNAc-GlcNAc linkage	N.D.	N.D.	NEB P0742
	Endoglycosidase F2	*E. miricola*	40	GlcNAc-GlcNAc linkage	N.D.	N.D.	NEB P0772
	Endoglycosidase F3	*E. minicola*	39	GlcNAc-GlcNAc linkage	17.1 ± 5.7	56.7 ± 13.4	NEB P0771
	Endoglycosidase M	*M. hiemalis*	85	GlcNAc-GlcNAc linkage	N.D.	N.D.	TCI A1651
V	Endoglycosidase S	*S. pyogenes*	136	GlcNAc-GlcNAc linkage	39.9 ± 1.9	68.4 ± 5.2	NEB P0741
Glycosyltransferases							
	α2-6 Sialyltransferase	*P. damsela*	59	Terminal Galactose	N.D.	N.D.	Sigma S2076
	α2-6 Sialyltransferase	*P. multocida*	46	Terminal Galactose	N.D.	N.D.	Sigma S1951
V	α2-6 Sialyltransferase (ST6Gal1)	*H. sapiens*	44	Terminal Galactose	7.8 ± 1.6	29.3 ± 0.4	Sigma SAE0090
	α2-6 Sialyltransferase (ST6Gal2)	*H. sapiens*	33	Terminal Galactose	N.D.	1.6 ± 1.2	R&D Systems 8330-GT
	α2-6 Sialyltransferase (ST6GalNAc4)	*H. sapiens*	31	Terminal Galactose	N.D.	1.2 ± 0.4	R&D Systems 6876-GT
	α2-3 Sialyltransferase (ST3-Gal1)	*H. sapiens*	33	Terminal Galactose	N.D.	2.1 ± 0.4	R&D Systems 6905-GT
	α2-3 Sialyltransferase (ST3-Gal2)	*H. sapiens*	35	Terminal Galactose	N.D.	1.2 ± 1.1	R&D Systems 7275-GT
V	β1-4 Galactosyltransferase 1	Homo sapiens	40	Terminal GlcNAc	7 ± 1.8	22.2 ± 1.1	Sigma SAE0093
	β1-4 Galactosyltransferase	*B. Taurus* (milk)	45	Terminal GlcNAc	3.1 ± 1	23 ± 1.2	Sigma G5507
V	N-Acetylglucosaminyltransferase 1	*H. sapiens*	48	(F)M3 glycoforms	8.4 ± 2.6*	17.6 ± 0.2*	R&D Systems 8334-GT
V	N-Acetylglucosaminyltransferase 3	*H. sapiens*	58	(F)A2 glycoforms	N.D.	6 ± 2.1	R&D Systems 7359-GT
V	N-Acetylglucosaminyltransferase 5	*H. sapiens*	65	(F)A2/(F)A3 glycoforms	N.D.	N.D.	R&D Systems 5469-GT
V	Fucosyltransferase 8	*H. sapiens*	64	(F)M3 glycoforms	1.7± 0.6	9.2 ± 2.1	R&D Systems 5768-GT

Table S1. **Activity screening of glycoengineering enzymes.** Enzyme activity was determined by using the SPGR protocol with 1mg human serum IgG (66.7 µM) and glycoengineering enzyme (0.25 µM) for 1 or 24 hours. HILIC-MS was employed for characterizing glycan structures and quantification. N.D. = not detectable. V= enzymes showed the highest activity in the screening (selected for SPGR). 0.1 ml Protein A resin was used for immobilization and the final reaction volume was 0.1 ml for all the reactions. Reactions in this screening were conducted using the working conditions suggested by the vendors without further optimization. *Glycoengineered IgG was used as the substrate for the screening.

	Exp.	Enzyme (Amount)	Substrate	Buffer	Mol % (E-to-S)	Weight % (E-to-S)	Reaction Time	Temp.	pH	Cation
	Fig S1A	Neuraminidase (2 µg)	Human serum IgG	50 mM Sodium acetate	0.7%	0.2%	1h	**Variable**	5.5	Ca^{2+}
	Fig S1B	Neuraminidase	Human serum IgG	50 mM Sodium acetate	**Variable**	**Variable**	1h	42°C	5.5	Ca^{2+}
	Fig S1C	Neuraminidase (16 µg)	Human serum IgG	50 mM Sodium acetate	5.6%	1.6%	**Variable**	42°C	5.5	Ca^{2+}
	Fig 2	Neuraminidase (32 µg)	Human serum IgG	50 mM Sodium acetate	11.2%	3.2%	4h	42°C	5.5	Ca^{2+}
	Fig S2A	Galactosidase S (12.5 µg)	Human serum IgG	50 mM Sodium acetate	0.75%	1.25%	1h	**Variable**	5.5	Ca^{2+}
	Fig S2B	Galactosidase S	Human serum IgG	50 mM Sodium acetate	**Variable**	**Variable**	1h	37°C	5.5	Ca^{2+}
	Fig S2C	Galactosidase S (75 µg)	Human serum IgG	50 mM Sodium acetate	4.5%	7.5%	**Variable**	37°C	5.5	Ca^{2+}
	Fig 2	Galactosidase S (37.5 µg)	Human serum IgG	50 mM Sodium acetate	2.25%	3.75%	16h	37°C	5.5	Ca^{2+}
	Fig S3A	N-Acetylglucosaminidase S (3 µg)	Human serum IgG	50 mM Sodium acetate	0.37%	0.3%	1h	**Variable**	5.5	Ca^{2+}
	Fig S3B	N-Acetylglucosaminidase S	Human serum IgG	50 mM Sodium acetate	**Variable**	**Variable**	1h	42°C	5.5	Ca^{2+}
	Fig S3C	N-Acetylglucosaminidase S (25 µg)	Human serum IgG	50 mM Sodium acetate	3.2%	2.5%	**Variable**	42°C	5.5	Ca^{2+}
	Fig 2	N-Acetylglucosaminidase S (35 µg)	Human serum IgG	50 mM Sodium acetate	4.2%	3.5%	4h	42°C	5.5	Ca^{2+}
	Fig S4A	Fucosidase O (1.32 mg)	FM3 IgG	50 mM Sodium acetate	400%	132%	3-days	37°C	4.5	None
	Fig S4C	Fucosidase O (0.33 mg)	Human serum IgG	50 mM Sodium acetate	100%	33%	5-days	37°C	4.5	None
	Fig S16A	Endoglycosidase S (27 µg)	Human serum IgG	50 mM Sodium acetate	3%	2.7%	4h	42°C	5.5	Ca^{2+}
	Fig S16B	Endoglycosidase S (3.4 µg)	Human serum IgG	50 mM Sodium acetate	0.37%	0.34%	1h	**Variable**	5.5	Ca^{2+}
	Fig S16C	Endoglycosidase S	Human serum IgG	50 mM Sodium acetate	**Variable**	**Variable**	1h	42°C	5.5	Ca^{2+}
	Fig S16D	Endoglycosidase S (13.6 µg)	Human serum IgG	50 mM Sodium acetate	1.5%	1.36%	**Variable**	42°C	5.5	Ca^{2+}
	Fig S5A	α2-6 Sialyltransferase (1.1 µg)	Human serum IgG	25 mM Tris-HCl	0.37%	0.11%	24h	**Variable**	7.5	Ca^{2+}, Mn^{2+}
	Fig S5B	α2-6 Sialyltransferase (1.1 µg)	Human serum IgG	25 mM Tris-HCl	0.37%	0.11%	24h	37°C	**Variable**	Ca^{2+}, Mn^{2+}
	Fig S5C	α2-6 Sialyltransferase (1.1 µg)	Human serum IgG	25 mM Tris-HCl	0.37%	0.11%	24h	37°C	7.5	**Variable**
	Fig S5D	α2-6 Sialyltransferase	Human serum IgG	25 mM Tris-HCl	**Variable**	**Variable**	1h	37°C	7.5	Mg^{2+}
	Fig S5E	α2-6 Sialyltransferase (44 µg)	Human serum IgG	25 mM Tris-HCl	14.8%	4.4%	**Variable**	37°C	7.5	Mg^{2+}
	Fig 2	α2-6 Sialyltransferase (15 µg)	Human serum IgG	25 mM Tris-HCl	5%	1.5%	16h	37°C	7.5	Mg^{2+}
	Fig S6A	β1-4 Galactosyltransferase 1 (10 µg)	Human serum IgG	25 mM Tris-HCl	3.7%	1%	1h	**Variable**	7	Na^{+}, Mn^{2+}
	Fig S6B	β1-4 Galactosyltransferase 1 (8 µg)	Human serum IgG	25 mM Tris-HCl	3%	0.8%	2h	50°C	**Variable**	Na^{2+}, Mn^{2+}
	Fig S6C	β1-4 Galactosyltransferase 1 (4 µg)	Human serum IgG	25 mM Tris-HCl	1.5%	0.4%	3h	50°C	7	**Variable**
	Fig S6D	β1-4 Galactosyltransferase 1	Human serum IgG	25 mM Tris-HCl	**Variable**	**Variable**	1h	50°C	7	Mn^{2+}
	Fig S6E	β1-4 Galactosyltransferase 1 (30 µg)	Human serum IgG	25 mM Tris-HCl	11.2%	3%	**Variable**	50°C	7	Mn^{2+}
	Fig 2	β1-4 Galactosyltransferase 1 (25 µg)	Human serum IgG	25 mM Tris-HCl	9.4%	2.5	16	50°C	7	Mn^{2+}
	Fig S8A	N-Acetylglucosaminyltransferase 1 (1.2 µg)	FM3 IgG	20 mM HEPES	0.37%	0.12%	4h	**Variable**	7	Na^{+}, Mn^{2+}
	Fig S8B	N-Acetylglucosaminyltransferase 1 (1.2 µg)	FM3 IgG	20 mM HEPES	0.37%	0.12%	4h	37°C	**Variable**	Na^{+}, Mn^{2+}
	Fig S8C	N-Acetylglucosaminyltransferase 1 (2.4 µg)	FM3 IgG	20 mM HEPES	0.74%	0.24%	2h	30°C	7.5	**Variable**
	Fig S8D	N-Acetylglucosaminyltransferase 1	FM3 IgG	20 mM HEPES	**Variable**	**Variable**	1h	30°C	7.5	Na^{+}, Ca^{2+}, Mg^{2+}, Mn^{2+}
	Fig S8E	N-Acetylglucosaminyltransferase 1 (15 µg)	FM3 IgG	20 mM HEPES	4.5%	1.5%	**Variable**	30°C	7.5	Na^{+}, Ca^{2+}, Mg^{2+}, Mn^{2+}
	Fig S8F	N-Acetylglucosaminyltransferase 1 (30 µg)	FM3 IgG	20 mM HEPES	**Variable**	**Variable**	**Variable**	30°C	7.5	Na^{+}, Ca^{2+}, Mg^{2+},Mn^{2+}
	Fig S7	N-Acetylglucosaminyltransferase 1 (20 µg)	FM3 IgG	20 mM HEPES	6%	2%	4	30°C	7.5	Na^{+}, Ca^{2+}, Mg^{2+}, Mn^{2+}
	Fig S9A	N-Acetylglucosaminyltransferase 3 (9 µg)	Human serum IgG	20 mM HEPES	1.5%	0.9%	24h	**Variable**	7	Na^{+}, Mn^{2+}
	Fig S9B	N-Acetylglucosaminyltransferase 3 (9 µg)	Human serum IgG	20 mM HEPES or MES	1.5%	0.9%	24h	37°C	**Variable**	Na^{+}, Mn^{2+}
	Fig S9C	N-Acetylglucosaminyltransferase 3 (9 µg)	Human serum IgG	20 mM MES	1.5%	0.9%	24h	30°C	6.5	**Variable**
	Fig S9D	N-Acetylglucosaminyltransferase 3	Human serum IgG	20 mM MES	**Variable**	**Variable**	1h	30°C	6.5	Na^{+}, Mn^{2+}
	Fig S9E	N-Acetylglucosaminyltransferase 3 (45 µg)	Human serum IgG	20 mM MES	7.5%	4.5%	**Variable**	30°C	6.5	Na^{+}, Mn^{2+}
	Fig 2	N-Acetylglucosaminyltransferase 3 (30 µg)	Human serum IgG	20 mM MES	5%	3%	16h	30°C	6.5	Na^{+}, Mn^{2+}
	Fig S10	N-Acetylglucosaminyltransferase 5 (33 µg)	Human serum IgG	20 mM HEPES	7.5%	3.3%	24h	30°C	7.5	Na^{+}, Mn^{2+}
	Fig S11	Fucosyltransferase (60 µg)	Defucosylated IgG	100 mM MES	14%	6%	5 days	37°C	7	Na^{+}

Table S2. Reaction conditions used for SPGR reactions in this work. Protein A resin (0.1 ml) was used for IgG (1 mg) immobilization for all the reactions. The final reaction volume was 0.1 ml. (F)M3 IgG was prepared from human serum IgG by applying sequential glycan remodeling using SPGR as described in this study. Cation ion concentration: 5 mM for Ca^{2+} ($CaCl_2$), 50 mM for Na^{+} (NaCl) and 10 mM for Mg^{2+} and Mn^{2+} ($MgCl_2$, $MnCl_2$).

	Serum IgG	Endo S-treated	Fuc treated	Featured terminal (Bi-antennary)				Mono-antennary			Bisecting		
				1	2	3	4	5	6	7	8	9	10
**Hydrodynamic diameter (nm)**	13.3±1.2	--	--	11.5±0.5	17.7±2	13.6±0.4	11.8±0.6	14.8±0.7	14.2±0.9	13.3±0.9	17.4±1.2	12.8±0.4	18±2.4
**Melting temperature (°C)**	66.7±0.7	56.4±1.4	--	63.4±0.4	64.8±0.7	66.3±0.3	66.1±0.2	61.4±0.5	62.9±0.7	63±0.4	64.8±1.1	64.5±0.3	65±0.7
**Aggregation temperature (°C)**	62±1.7	48.6±1.3	--	59±2.3	63.3±1	66.1±0.5	64.3±1.1	62.2±0.5	62.2±1.2	63±0.9	60.9±1.2	63.3±0.4	64.9±0.6
**FcγR I binding (EC_{50}, 10^{-7} g/ml)**	1.9±0.4	--	1.6±0.4	2.2±0.7	2.3±0.5	1.7±0.5	2±0.2	2.3±0.3	2.5±0.2	2.4±0.3	0.8±0.1	0.9±0.1	1.2±0.3

Table S3. Physical and biochemical properties of SPGR-engineered IgGs. Fuc: fucosidase

	Enzyme	Donor	Substrate glycan species (Acceptor) (excluding low-abundant, non-detectable glycoforms)
	Neuraminidase	N.A.	(F)A2(B)G2S2, (F)A2(B)G2S1, (F)A2(B)G1S1
	Galactosidase S	N.A.	(F)A2(B)G2S1, (F)A2(B)G2, (F)A2(B)G1
	N-Acetylglucosaminidase S	N.A.	(F)A2(B)G1, (F)A2(B)
	Fucosidase O	N.A.	All fucosylated IgG glycans
	Endoglycosidase S	N.A.	All IgG glycans
	α2-6 Sialyltransferase	Cytidine-5'-monophospho-N-acetylneuraminic acid (CMP-NANA)	(F)A2(B)G2, (F)A2(B)G1, (F)A2(B)G2S1
	β1-4 Galactosyltransferase 1	Uridine 5'-diphosphogalactose (UDP-Gal)	(F)A2(B), (F)A2(B)G1
	N-Acetylglucosaminyltransferase 1	Uridine 5'-diphospho-N-acetylglucosamine (UDP-GlcNAc)	(F)M3 (glycoengineered IgG)
	N-Acetylglucosaminyltransferase 3	Uridine 5'-diphospho-N-acetylglucosamine (UDP-GlcNAc)	(F)A2, (F)A2G1
	Fucosyltransferase	Guanosine 5'-diphospho-β-L-fucose (GDP-Fucose)	M3 (glycoengineered IgG)

Table S4. Saccharide donor and acceptors in each enzymatic reaction. The substrate glycan species (acceptors) were listed based on our observation in this study.

Supplementary Figures

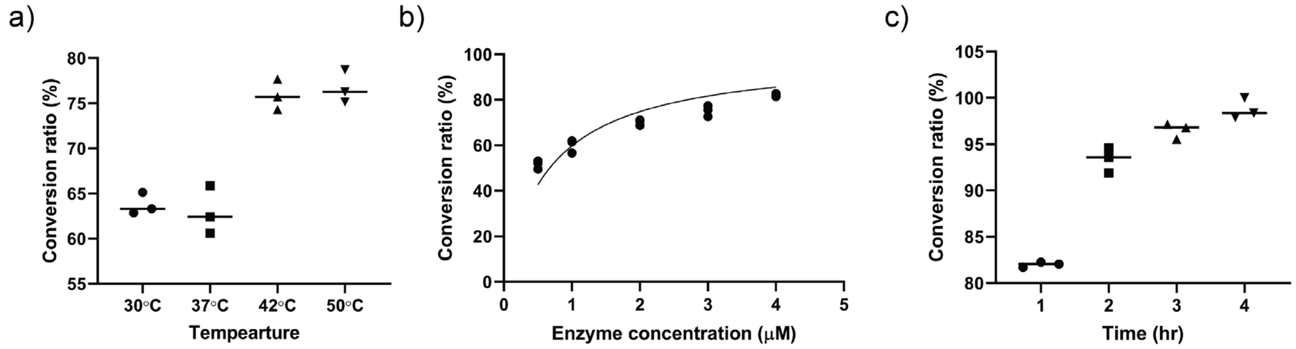

Figure S1. Characterization of Neuraminidase (*C. perfringens*) activity and its working condition optimization. **(a)** Temperature optimization (N=3). **(b)** Dose-dependent experiment (N=3). **(c)** Time-course study (N=3). Refer to **Table S2** for reaction conditions.

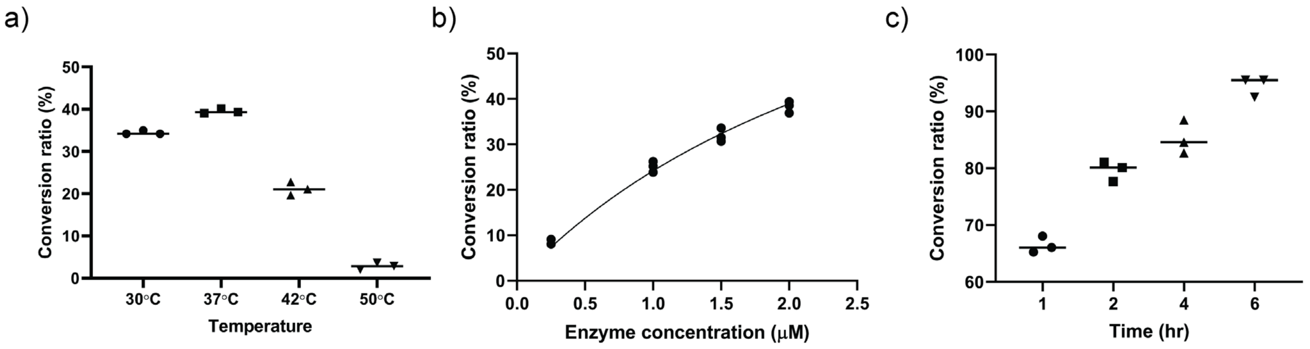

Figure S2. Characterization of Galactosidase S (*S. pneumoniae*) activity and its working condition optimization. **(a)** Temperature optimization (N=3). **(b)** Dose-dependent experiment (N=3). **(c)** Time-course study (N=3). Refer to **Table S2** for reaction conditions.

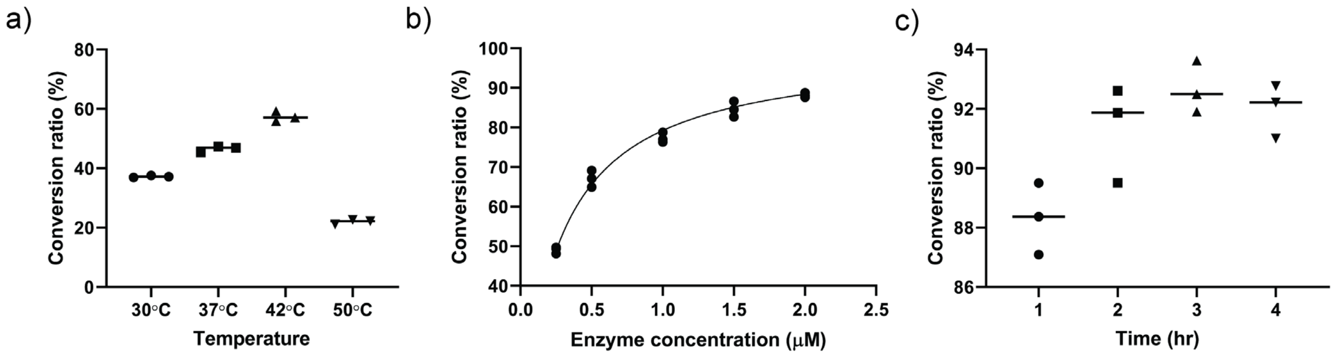

Figure S3. Characterization of *N*-Acetylglucosaminidase S (*S. pneumoniae*) activity and its working condition optimization. **(a)** Temperature optimization (N=3). **(b)** Dose-dependent experiment (N=3). **(c)** Time-course study (N=3). Refer to **Table S2** for reaction conditions.

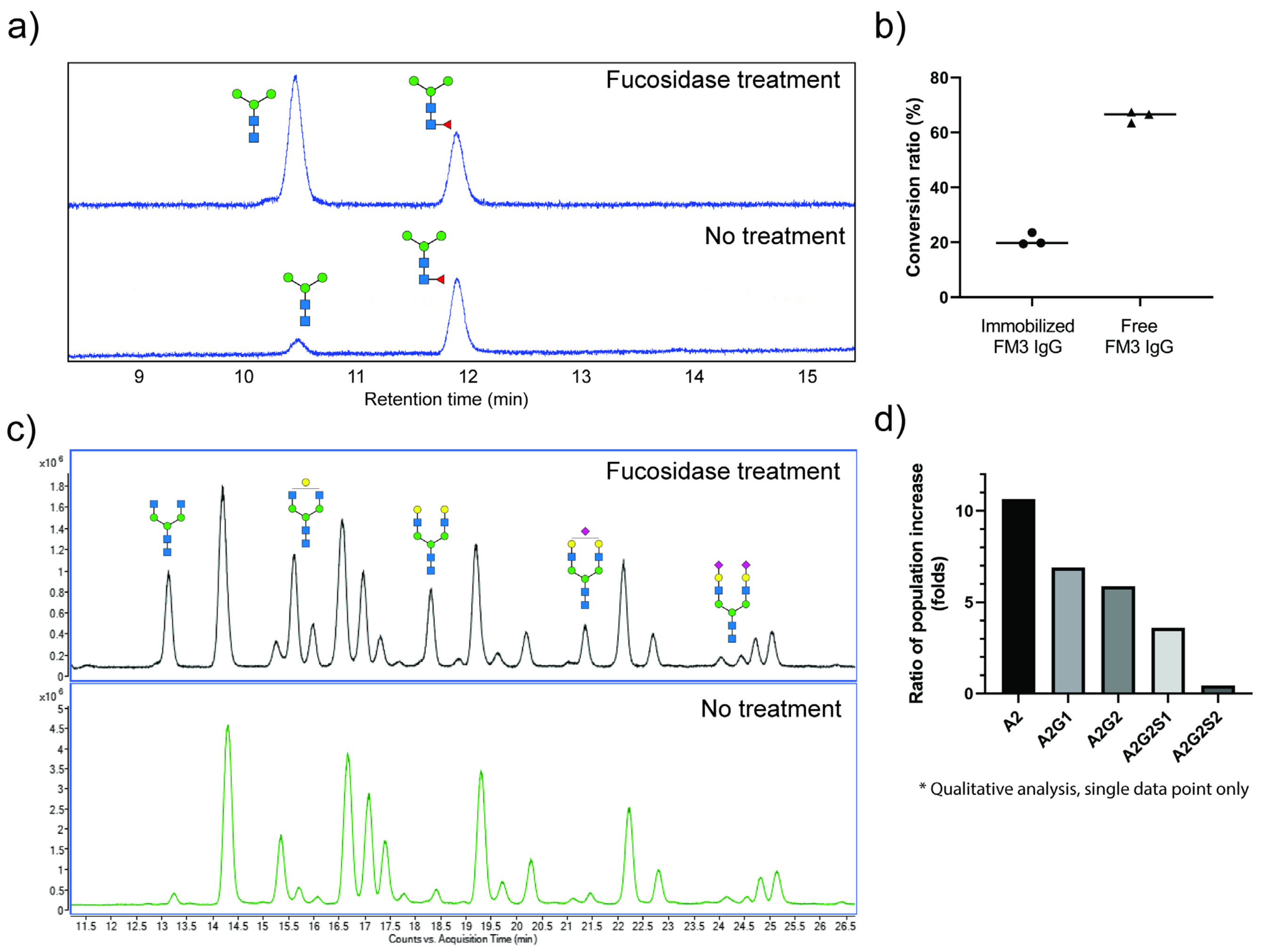

Figure S4. Characterization of fucosidase (*Candidatus Omnitrophica*) activity on intact IgG. **(a)** A 3-days reaction with intact IgG bearing (F)M3 glycans revealed the activity of fucosidase. The (F)M3 glycoforms were prepared using SPGR. **(b)** Comparison of conversion ratio between reactions with immobilized IgG and free IgG (N=3). **(c)** A 5-days reaction with intact IgG (no immobilization) revealed the broad substrate spectrum of the enzyme. Defucosylated populations were increased (highlighted with glycan images). See supplementary discussion for more details. **(d)** Fucosidase activity decreased as the structural complexity of glycans increased. The data was calculated based on the changes of peak area shown in the chromatograms **(c)** Qualitative analysis only (N=1). Refer to **Table S2** for reaction conditions.

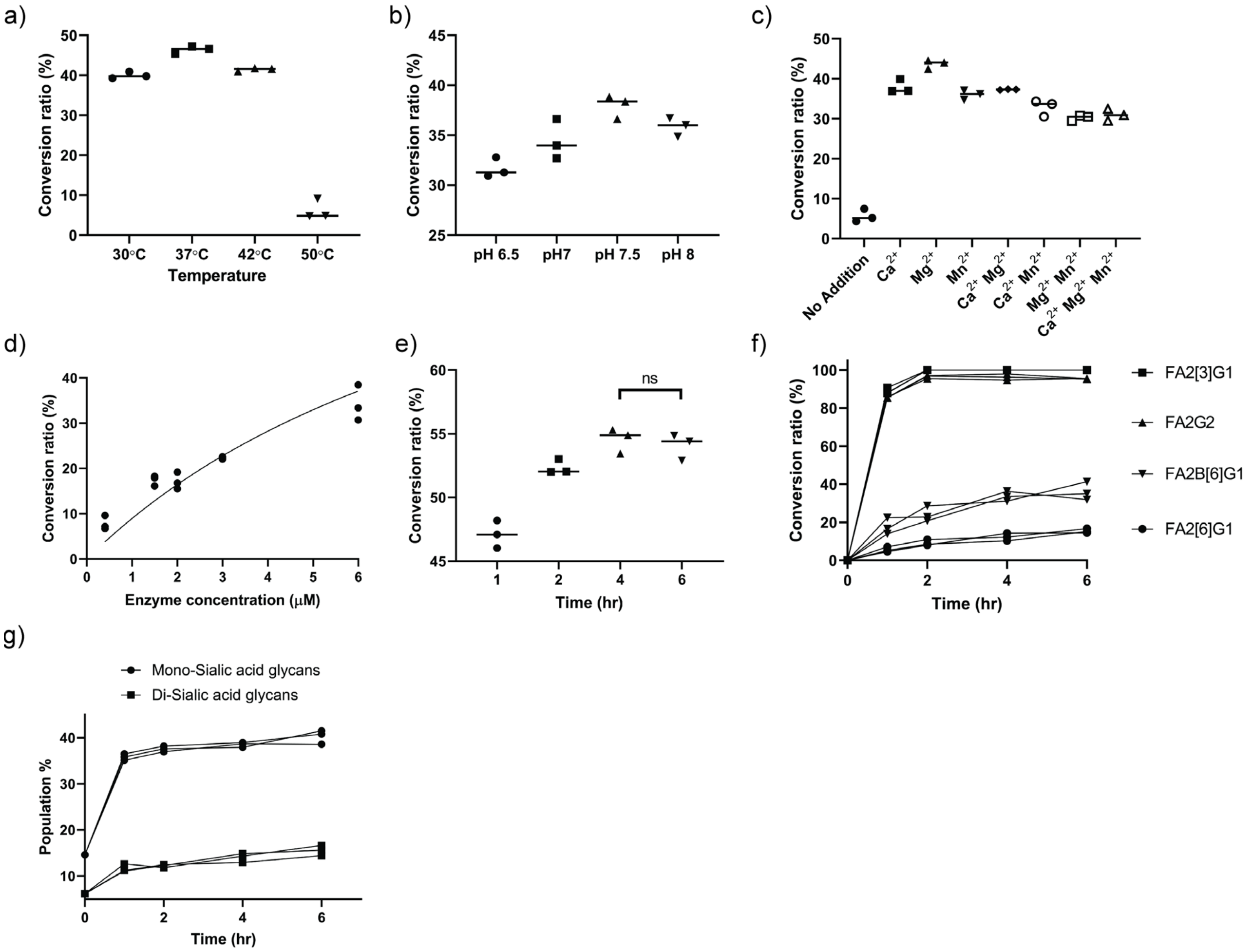

Figure S5. Characterization of α2-6 Sialyltransferase (*H. sapiens*) activity and its working condition optimization. **(a)** Temperature optimization (N=3). **(b)** pH optimization (N=3). **(c)** Cation optimization (N=3). **(d)** Dose-dependent experiment (N=3). **(e)** Time-course study (N=3, t-test: p=0.58). ns: not significant. Conversion ratio calculations for α2-6 Sialyltransferase reactions were based on the consumption of non-sialylated glycans with terminal galactoses. **(f)** Comparison of enzyme activity between different IgG glycoforms. The data was collected from the time-course experiments (N=3). **(g)** Comparison of enzyme activity between the installation of 1st and 2nd sialic acid (N=3). Refer to **Table S2** for reaction conditions.

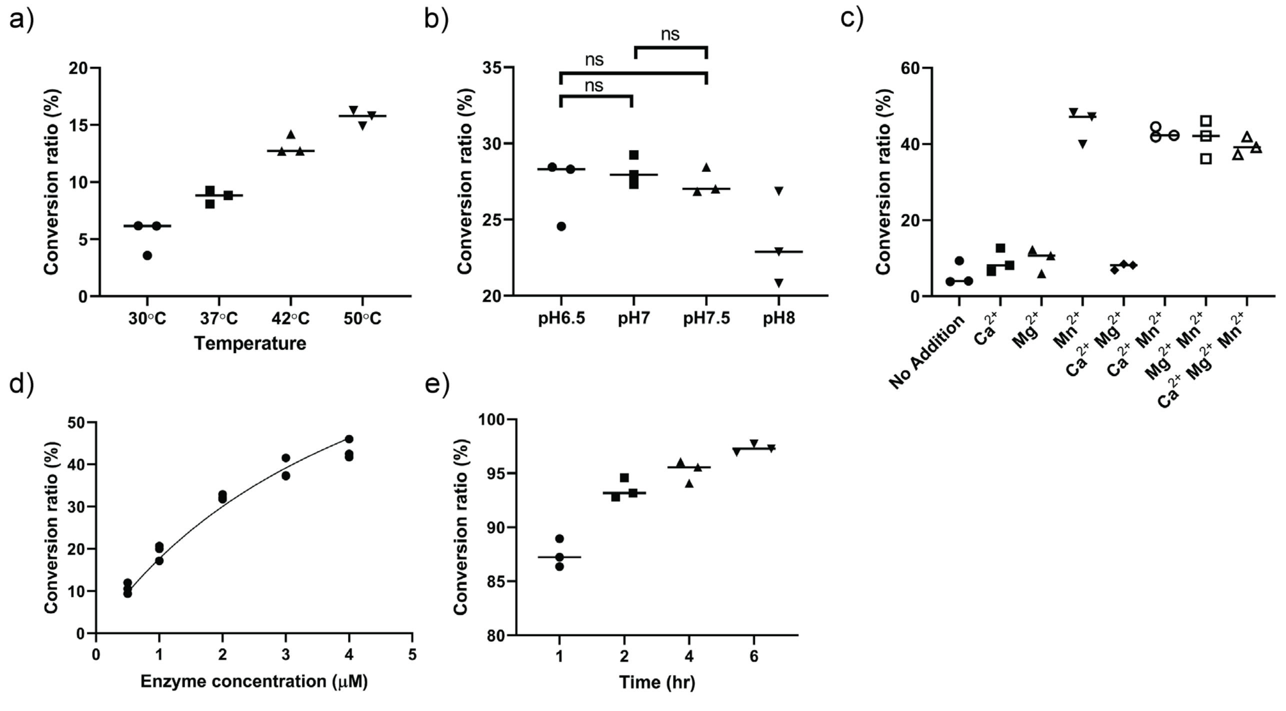

Figure S6. Characterization of β1-4 Galactosyltransferase 1 (*H. sapiens*) activity and its working condition optimization. **(a)** Temperature optimization (N=3). **(b)** pH optimization (N=3, t-test). ns: not significant. **(c)** Cation optimization (N=3). **(d)** Dose-dependent experiment (N=3). **(e)** Time-course study (N=3). Refer to **Table S2** for reaction conditions.

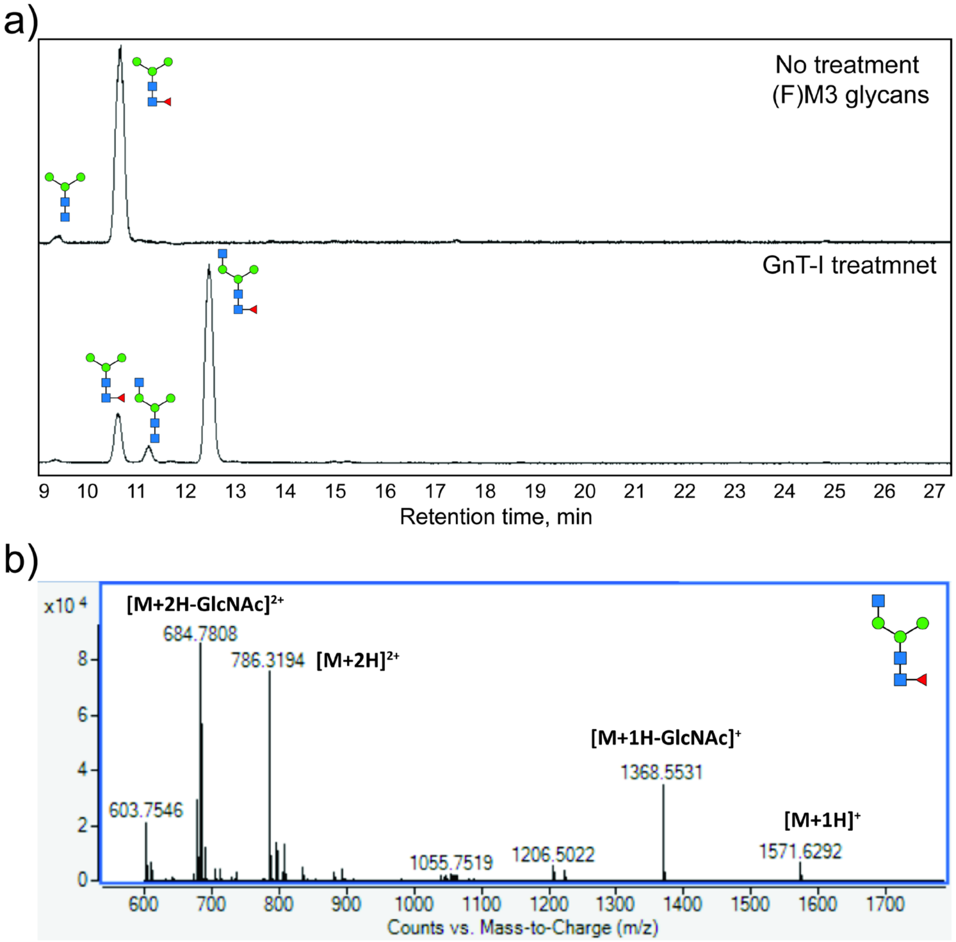

Figure S7. Characterization of *N*-Acetylglucosaminyltransferases I (GnT-I, MGAT1) activity. **(a)** Chromatographic analyses revealed GnT-I activity on (F)M3 glycans prepared by SPGR. **(b)** Mass spectrometry confirmed the formation of (F)A1[3] glycans. Glycan samples for the mass spectrometry analyses were labeled by RapiFlour-MS probe (MW=312.4) from Waters. On-column cleavage of terminal GlcNAc residues was often observed in HPLC-MS analyses. The IgG substrate bearing (F)M3 glycans was prepared by SPGR.

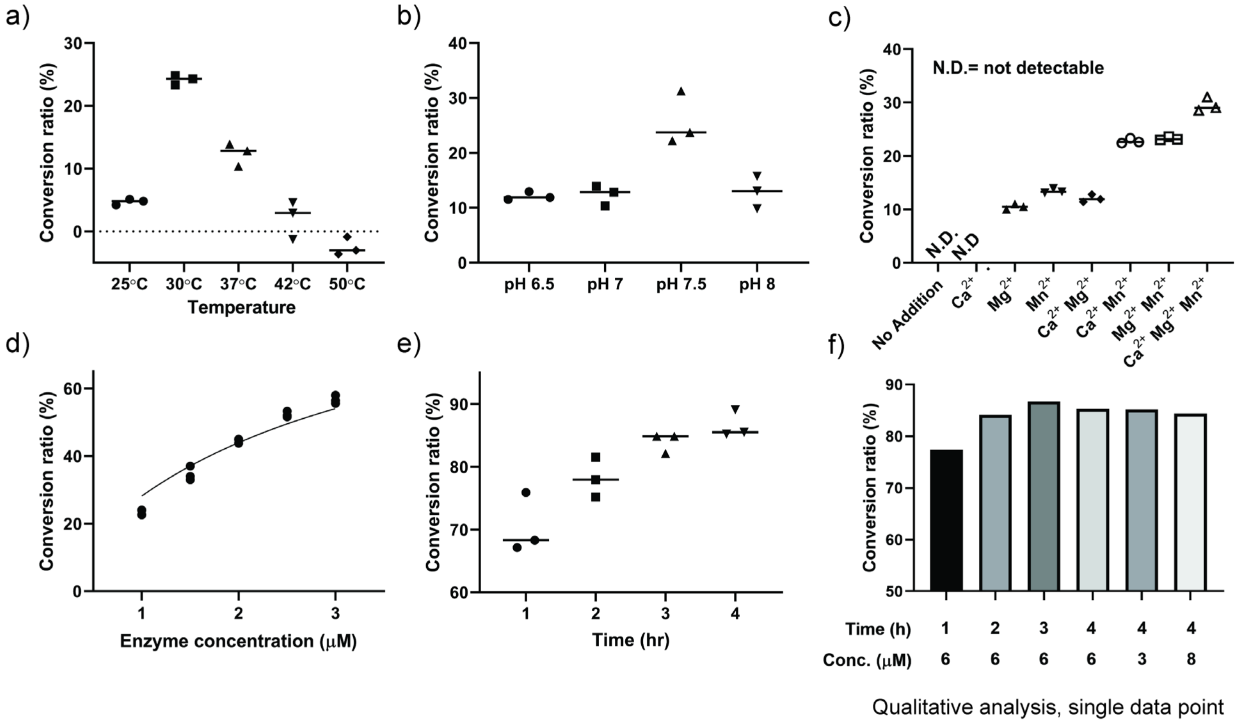

Figure S8. Characterization of *N*-Acetylglucosaminyltransferase 1 (GnT-I, *H. sapiens*) activity and its working condition optimization. **(a)** Temperature optimization (N=3). **(b)** pH optimization (N=3). **(c)** Cation optimization (N=3). **(d)** Dose-dependent experiment (N=3). **(e)** Time-course study (N=3). **(f)** The conversion ratio reached a plateau at ~ 85%. Qualitative analysis only (N=1). Refer to **Table S2** for reaction conditions.

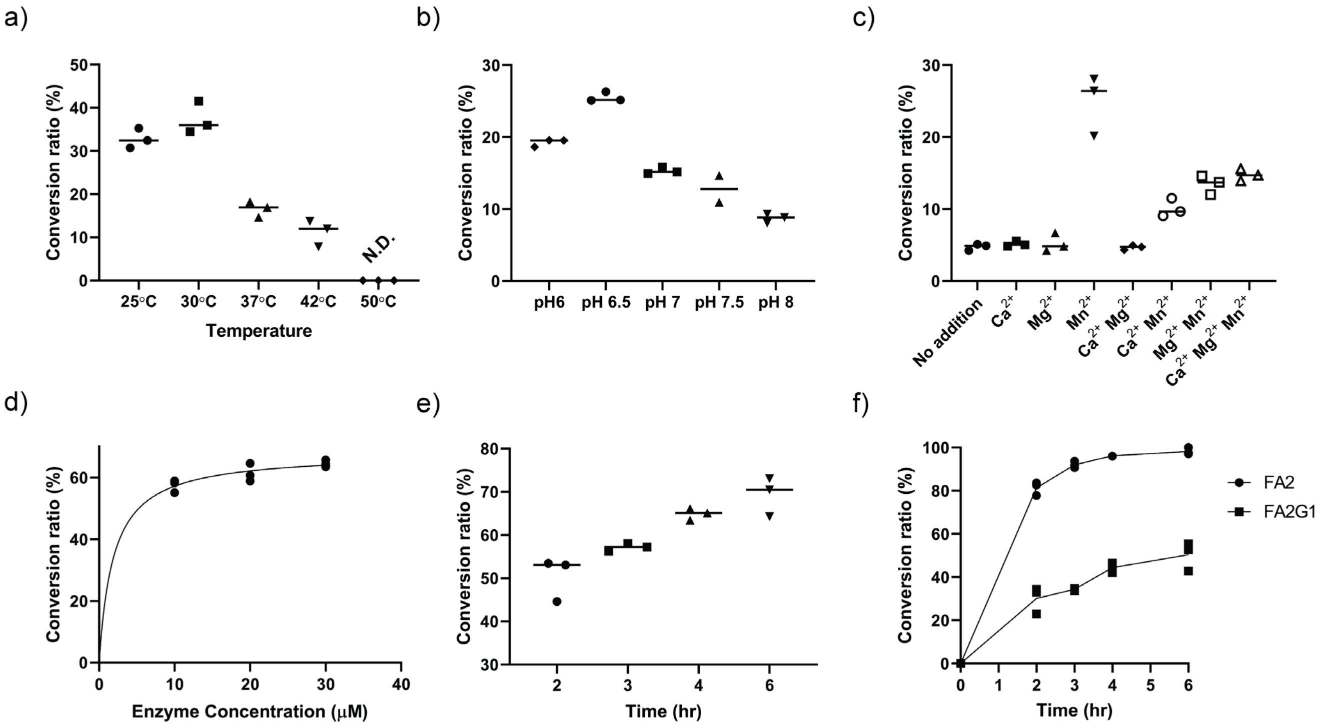

Figure S9. Characterization of *N*-Acetylglucosaminyltransferase 3 (GnT-III, *H. sapiens*) activity and its working condition optimization. **(a)** Temperature optimization (N=3). **(b)** pH optimization (N=3). **(c)** Cation optimization (N=3). **(d)** Dose-dependent experiment (N=3). **(e)** Time-course study (N=3). **(f)** Comparison of enzyme activity (in conversion ratio) between different IgG glycoforms. The data was collected from the time-course experiments (N=3). Refer to **Table S2** for reaction conditions.

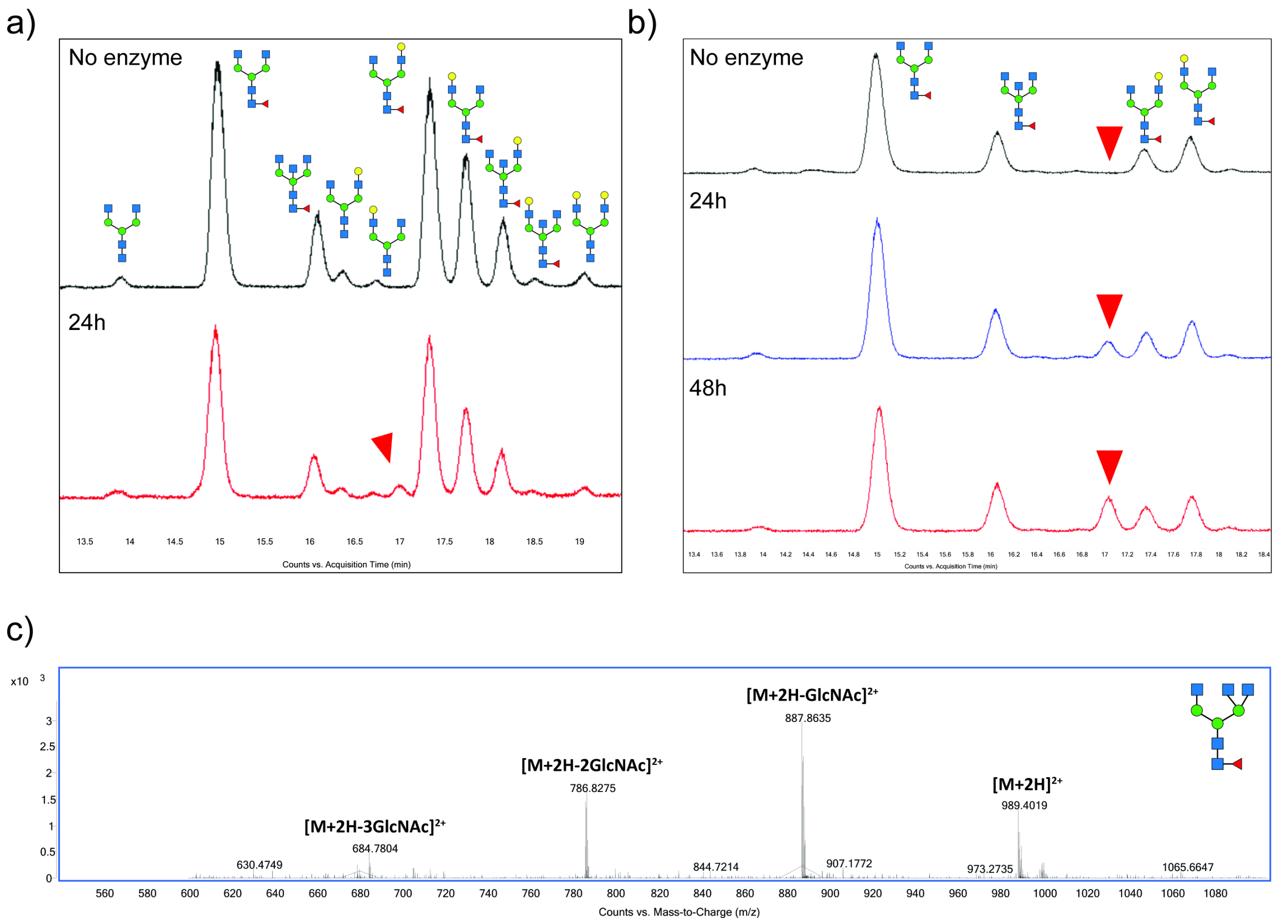

Figure S10. *In vitro* activity of *N*-Acetylglucosaminyltransferase 5 (GnT-V, *H. sapiens*) on intact human serum IgG immobilized on protein A resin. **(a)** GnT-V reaction with native human IgG. A new peak (indicated by red arrowhead) was found after a 24 hours reaction. **(b)** GnT-V reaction with glycoengineered human IgG. Human serum IgG was first treated with galactosidase in order to minimize the overlapping between the product signal and the FA2G1 glycan signals. A signal increase in the newly found peak was observed when the incubation time was increased. **(c)** Mass spectrometry analysis of the newly formed peak after the GnT-V reaction. The molecular weight (reported in m/z) of FA3 glycan was confirmed. The fragments resulting from on-column cleavage of GlcNA also supported that this analyte contains three terminal GlcNAc. The newly formed FA3 glycan has a 1-minute shift in retention time from that of FA2B glycan, despite they share the same molecular weight. Glycan samples for the mass spectrometry analyses showing here were labeled by RapiFlour-MS probe from Waters (MW=312.4).

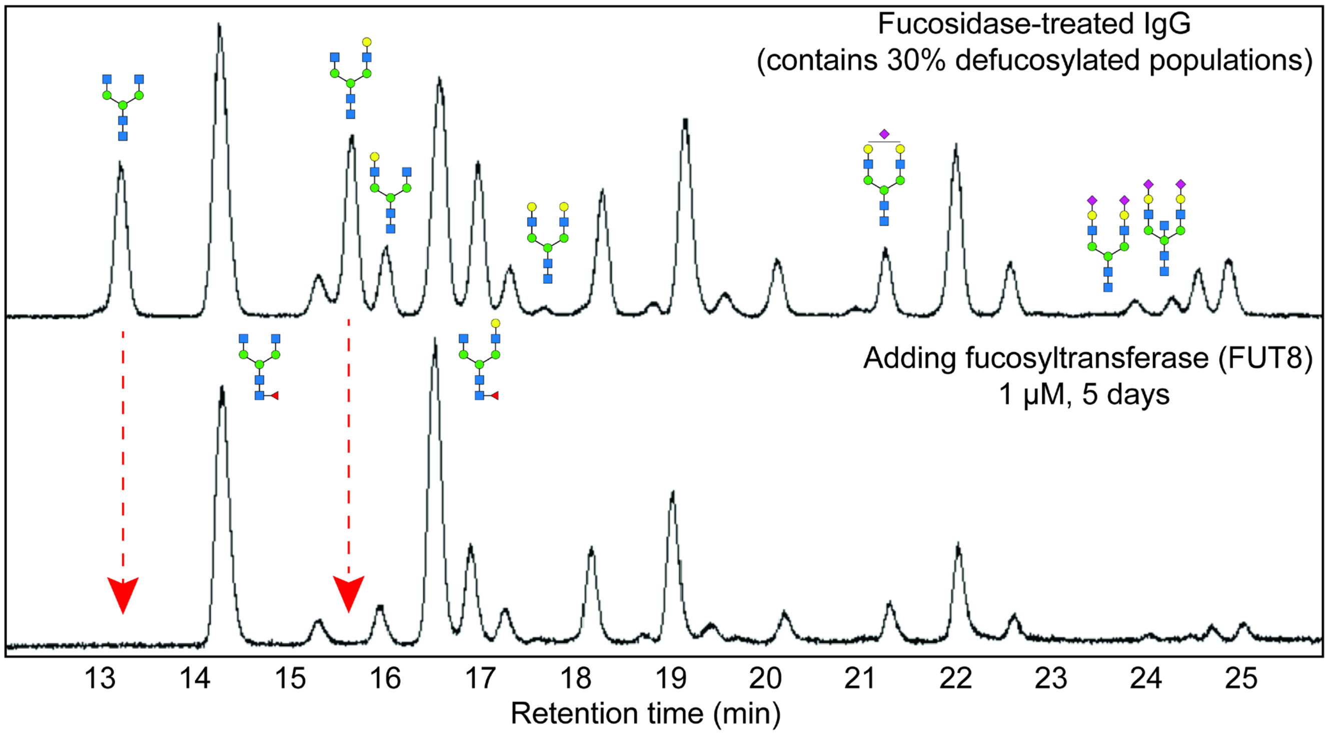

Figure S11. Characterization of fucosyltransferase (*Homo sapiens,* FUT8) activity. Serum IgG was treated with fucosidase (100 mol%) for 5 days. The resulting product (30% of the IgG population was defucosylated) was then used for the FUT8 reaction. Red arrows indicate the glycoforms consumed by FUT8. Refer to **Table S2** for reaction conditions.

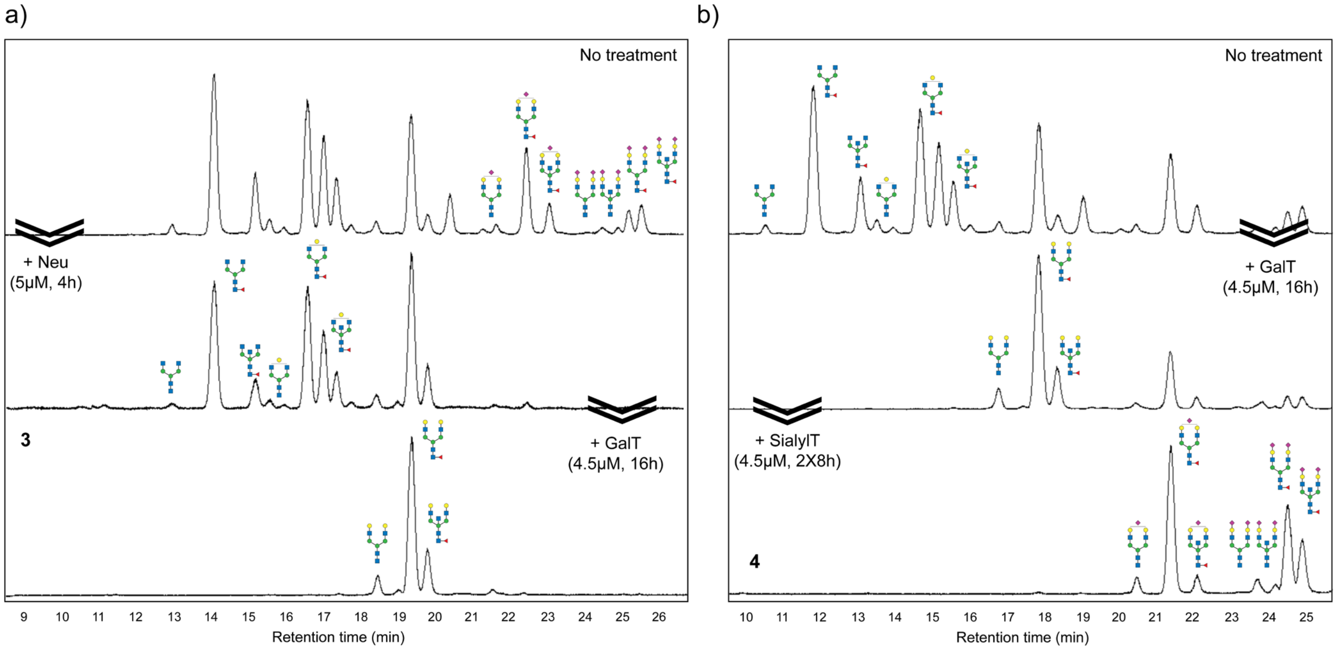

Figure S12. Chromatogram of fluorescently labeled glycans collected from IgG undergoing successive glycan remodeling using SPGR. **(a)** Harmonization of terminal residues into galactose. **(b)** Harmonization of terminal residues into sialic acid. The reactions were conducted on 1 mg human serum IgG immobilized on 0.1 ml protein A resin. Please refer **Figure 4** for the sample numbering and **Table 1** for working conditions.

a)

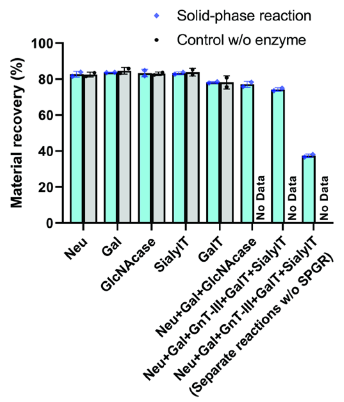

	Eq.	pH	Temp.	Time	Cation
Neu	0.1	5.5	42°C	4h	Ca^{2+}
Gal	0.2	5.5	37°C	16h	Ca^{2+}
GlcNAcase	0.05	5.5	42°C	4h	Ca^{2+}
SialylT	0.1	7.5	37°C	16h	Mg^{2+}
GalT	0.1	7	50°C	16h	Mn^{2+}
GnT-III	0.05	6.5	30°C	16h	Na^{+} Mn^{2+}

b)

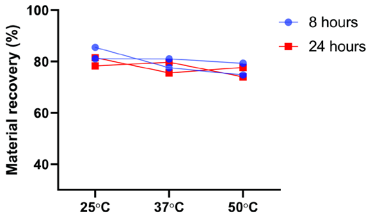

Figure S13. Studies of material recovery after SPGR reactions. **(a)** Recovery ratio after single-step reactions and multi-step reactions (N=2). Optimized reaction conditions that ensure full conversion were used, as summarized in the inserted table. In control experiments, glycoengineering enzymes were not added to the reactions. Reaction scale: 1 mg IgG. **(b)** High reaction temperature resulted in slight decrease of material recovery (N=2). Abbreviations: Neu: neuraminidase; Gal: galactosidase; GlcNAcase: *N*-Acetylglucosaminidase; SialylT: sialyltransferase; GalT: galactosyltransferase; GnT-III: *N*-Acetylglucosaminyltransferase III.

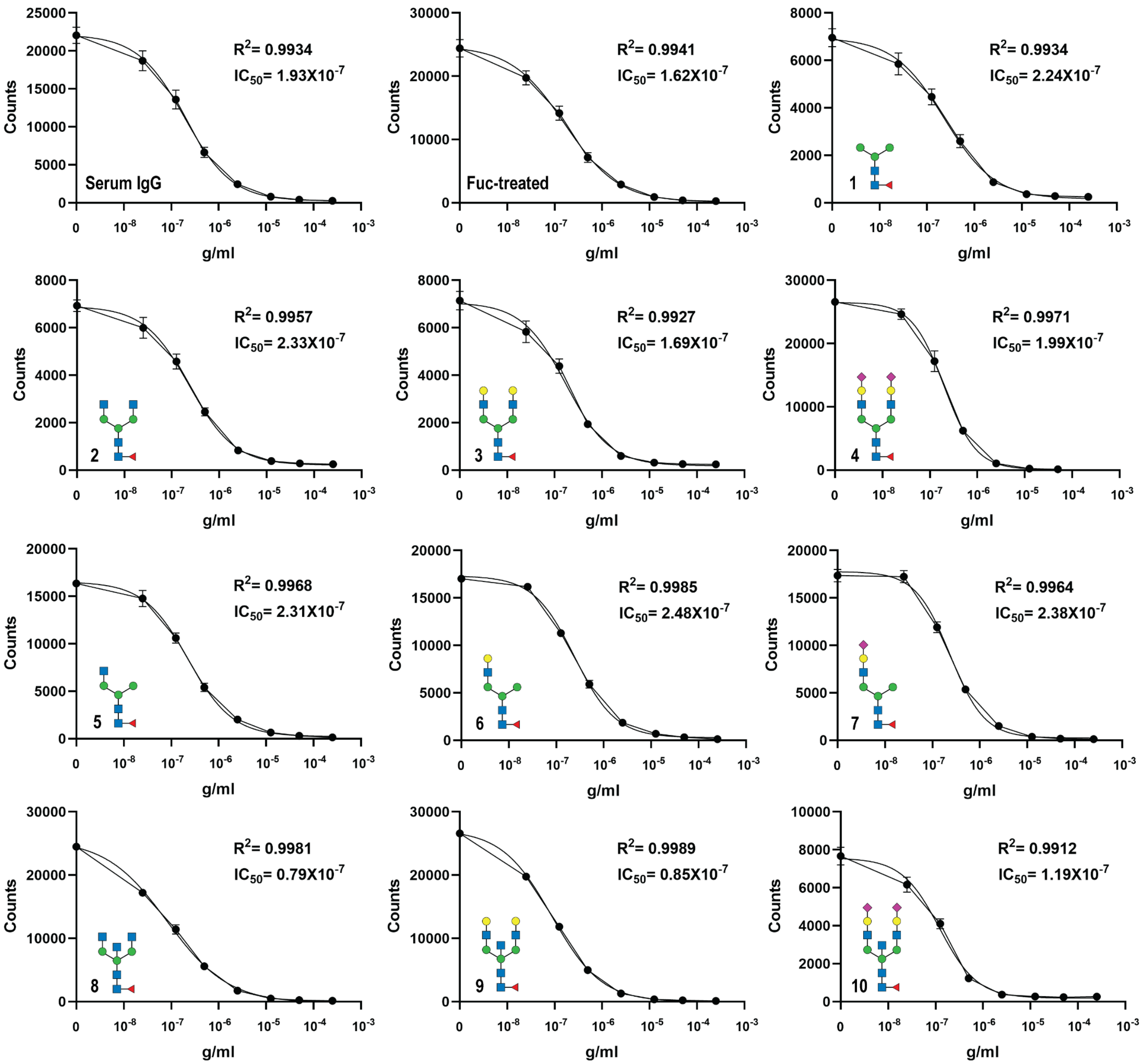

Figure S14. Investigating the binding affinity between SPGR-engineered IgG and Fcγ Receptor I using competition assays. IgG-FcRI interaction resulted in a signal decrease. Please note that all the IgG samples (**1**-**10**) contained a ~5% defucosylated population. The biantennary samples (**2**-**4**) had ~10% bisecting glycoforms; while the mono-antennary samples (**5**-**7**) also had ~10% (F)M3 glycans due to the GlcNAcase activity of GnT-I. Sialylated samples (**4** & **10**) possessed about 1:1 mono- and bi-sialylated populations. The Fucosidase-treated sample contained a 30% defucosylated glycan population. Effective concentration that leads to 50% signal reduction (EC_{50}) was calculated using IC_{50} curve fitting by GraphPad Prism 8 (Dose-response, inhibition). Sample size: N=3.

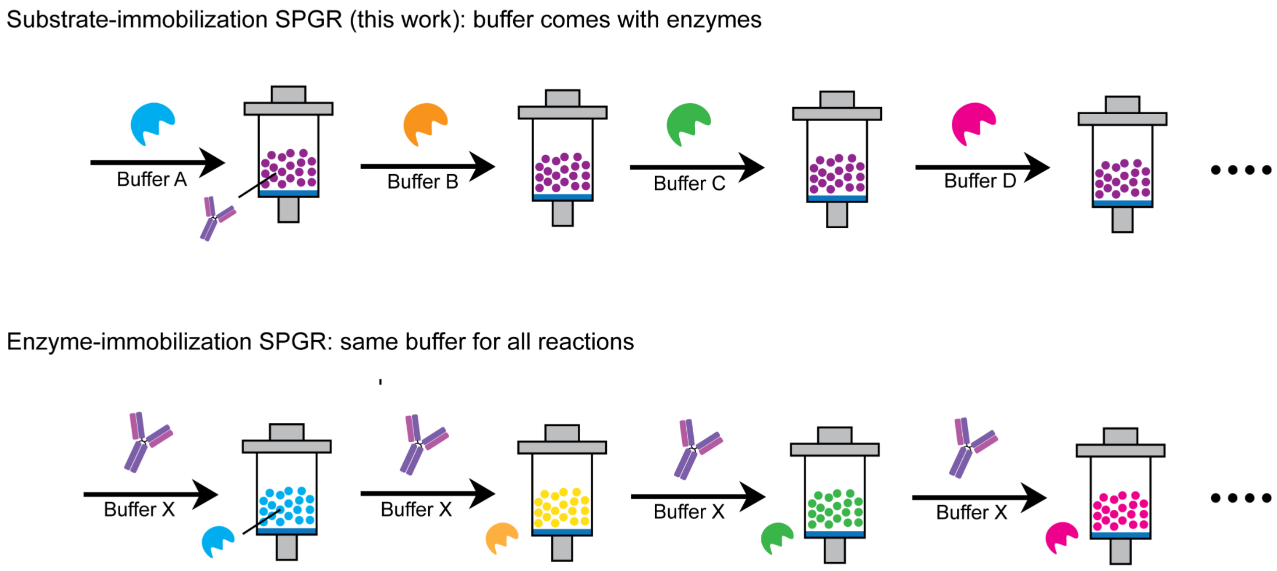

Figure S15. Comparison between substrate immobilization and enzyme immobilization in SPGR.

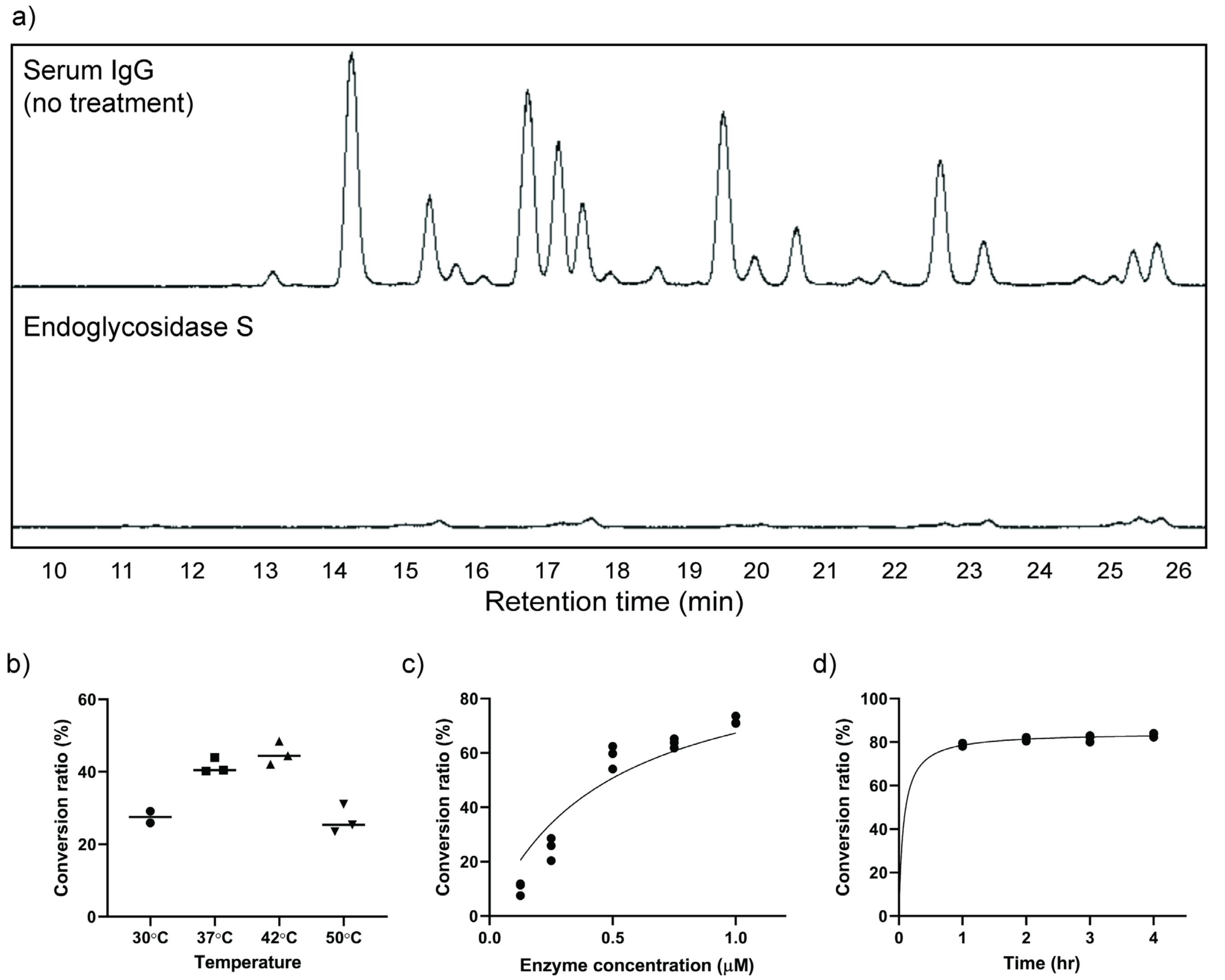

Figure S16. Characterization of endoglycosidase (*Streptococcus pyogenes*) activity and its working condition optimization. **(a)** Chromatography of IgG glycans before and after endo S treatment. **(b)** Temperature optimization (N=3). **(c)** Dose-dependent experiment (N=3). **(d)** Time-course study (N=3). Refer to **Table S2** for reaction conditions.

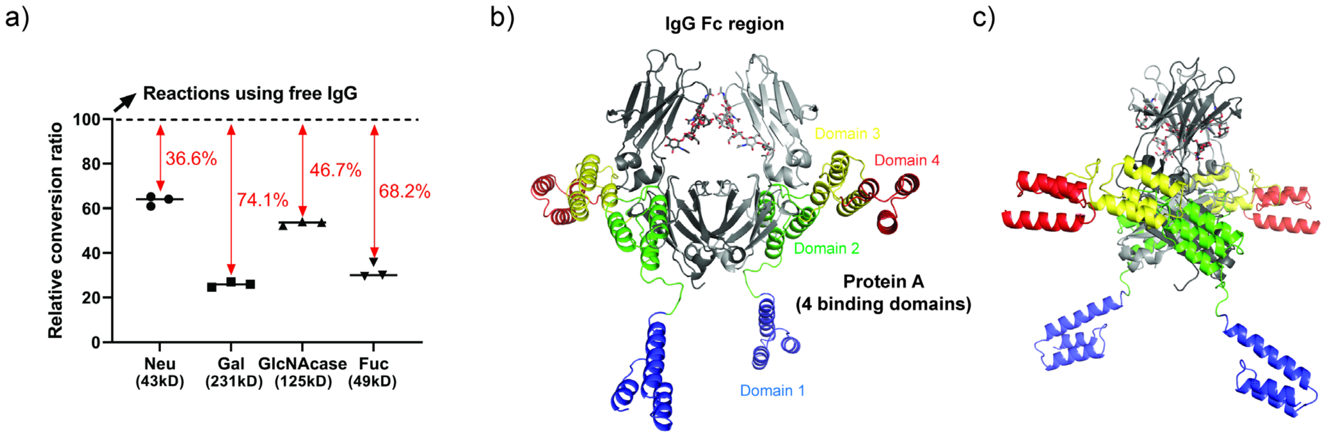

Figure S17. (a) Comparison of enzyme activity between the use of free and immobilized IgG as substrates (N=3). **(b-c)** Computational modeling of IgG Fc region and protein A interaction. **(b)** Front-view and **(c)** side-view of IgG Fc-protein A complex. Gray: IgG Fc region; blue: protein A binding domain 1; green: protein A binding domain 2; yellow: protein A binding domain 3; red: protein A binding domain 4. Bone structure: glycans.

Supplementary References

1 Li, T., Li, C., Quan, D. N., Bentley, W. E. & Wang, L. X. Site-specific immobilization of endoglycosidases for streamlined chemoenzymatic glycan remodeling of antibodies. *Carbohydr Res* **458-459**, 77-84, doi:10.1016/j.carres.2018.02.007 (2018).

2 Ruzic, L., Bolivar, J. M. & Nidetzky, B. Glycosynthase reaction meets the flow: Continuous synthesis of lacto-N-triose II by engineered β-hexosaminidase immobilized on solid support. *Biotechnology and bioengineering* **117**, 1597-1602, doi:10.1002/bit.27293 (2020).

3 Freeze, H. H. & Kranz, C. Endoglycosidase and glycoamidase release of N-linked glycans. *Curr Protoc Mol Biol* **17**, doi:10.1002/0471142727.mb1713as89 (2010).

4 Collin, M. & Olsén, A. EndoS, a novel secreted protein from Streptococcus pyogenes with endoglycosidase activity on human IgG. *EMBO J* **20**, 3046-3055, doi:10.1093/emboj/20.12.3046 (2001).

5 Fan, J. Q. *et al.* Transfer of Man9GlcNAc to L-fucose by endo-beta-N-acetylglucosaminidase from Arthrobacter protophormiae. *Glycoconj J* **13**, 643-652, doi:10.1007/bf00731453 (1996).

6 Yamamoto, K., Kadowaki, S., Watanabe, J. & Kumagai, H. Transglycosylation activity of Mucor hiemalis endo-beta-N-acetyl-glucosaminidase which transfers complex oligosaccharides to the N-acetylglucosamine moieties of peptides. *Biochem Biophys Res Commun* **203**, 244-252, doi:10.1006/bbrc.1994.2174 (1994).

7 Huang, W., Giddens, J., Fan, S.-Q., Toonstra, C. & Wang, L.-X. Chemoenzymatic Glycoengineering of Intact IgG Antibodies for Gain of Functions. *Journal of the American Chemical Society* **134**, 12308-12318, doi:10.1021/ja3051266 (2012).

8 Mizuochi, T., Amano, J. & Kobata, A. New evidence of the substrate specificity of endo-beta-N-acetylglucosaminidase D. *J Biochem* **95**, 1209-1213, doi:10.1093/oxfordjournals.jbchem.a134711 (1984).

9 Deisenhofer, J. Crystallographic refinement and atomic models of a human Fc fragment and its complex with fragment B of protein A from Staphylococcus aureus at 2.9- and 2.8-.ANG. resolution. *Biochemistry* **20**, 2361-2370, doi:10.1021/bi00512a001 (1981).

10 Kiyoshi, M., Tsumoto, K., Ishii-Watabe, A. & Caaveiro, J. M. M. Glycosylation of IgG-Fc: a molecular perspective. *International Immunology* **29**, 311-317, doi:10.1093/intimm/dxx038 (2017).
