# Supplementary material for: Successive remodeling of IgG glycans using a solid-phase enzymatic platform
Source: Commun Biol. 2022 Apr 7;5:328. doi: 10.1038/s42003-022-03257-4 (PMC8990068; doi:10.1038/s42003-022-03257-4)
Supplement: Supplementary file 3 — Description of Additional Supplementary Files [file 42003_2022_3257_MOESM3_ESM.pdf]

Description of Additional Supplementary Files

File name: Supplementary Data 1
Description: Source data used for diagram plotting and analyses in Figure 1c, 2, 3, 5, and Supplementary Figure 1-14, 16, and 17.
