# Supplementary figures and images for: Successive remodeling of IgG glycans using a solid-phase enzymatic platform
Source: Commun Biol. 2022 Apr 7;5:328. doi: 10.1038/s42003-022-03257-4 (PMC8990068; doi:10.1038/s42003-022-03257-4)

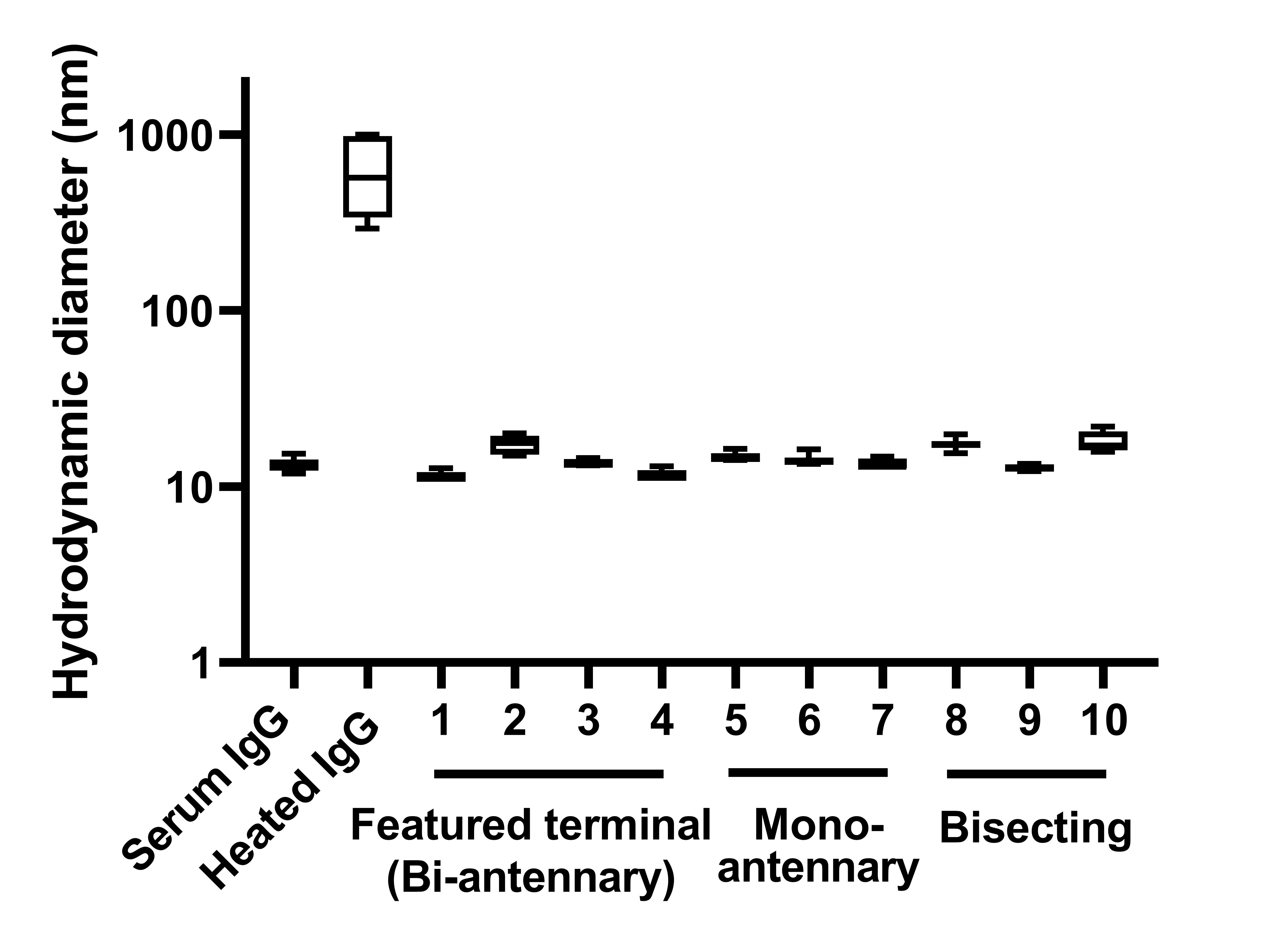

Supplement: Supplementary file 4 — Supplementary Data 1 [file 42003_2022_3257_MOESM4_ESM.zip › Source Data/Figure 5a-c/Figure 5a.png]

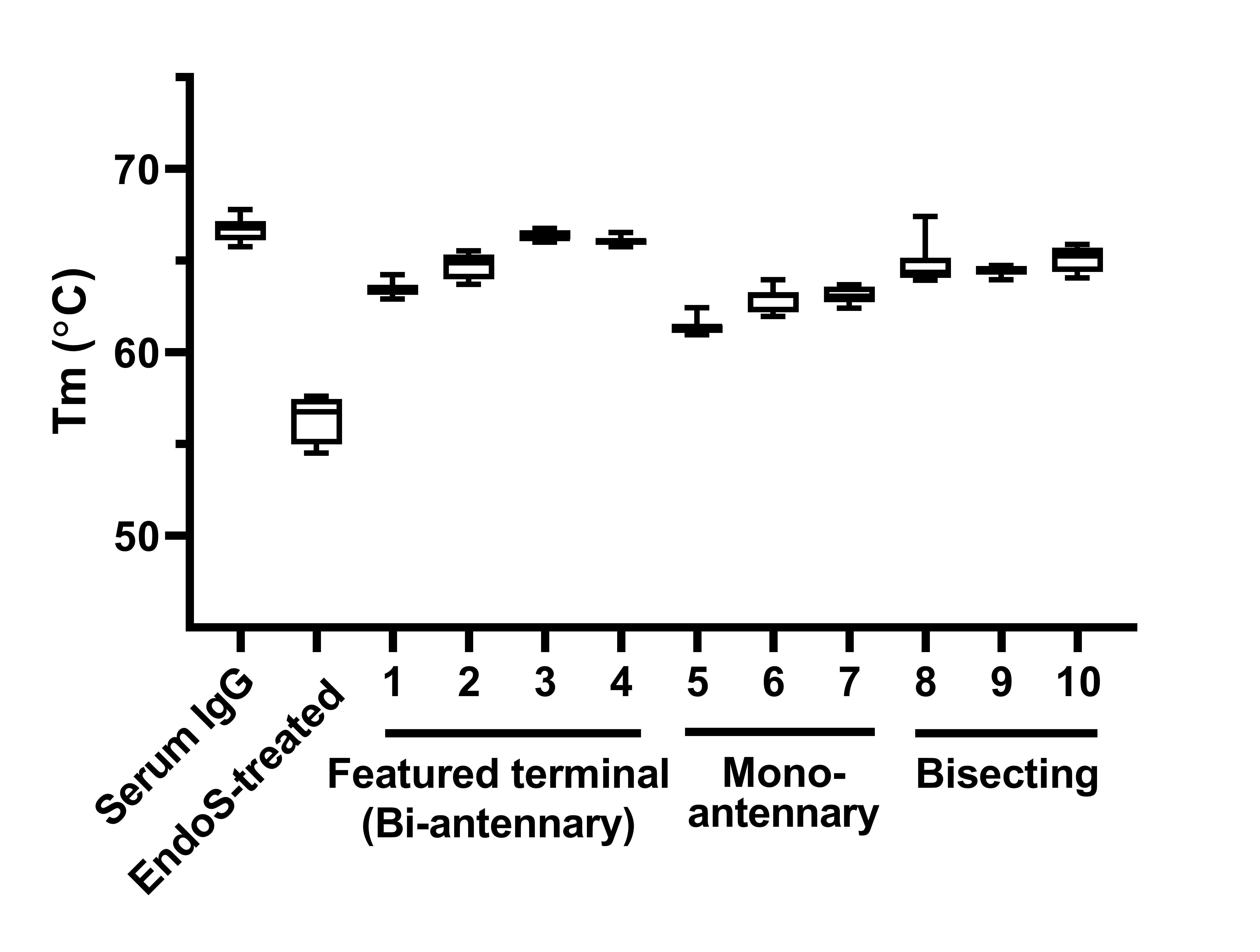

Supplement: Supplementary file 4 — Supplementary Data 1 [file 42003_2022_3257_MOESM4_ESM.zip › Source Data/Figure 5a-c/Figure 5b.png]

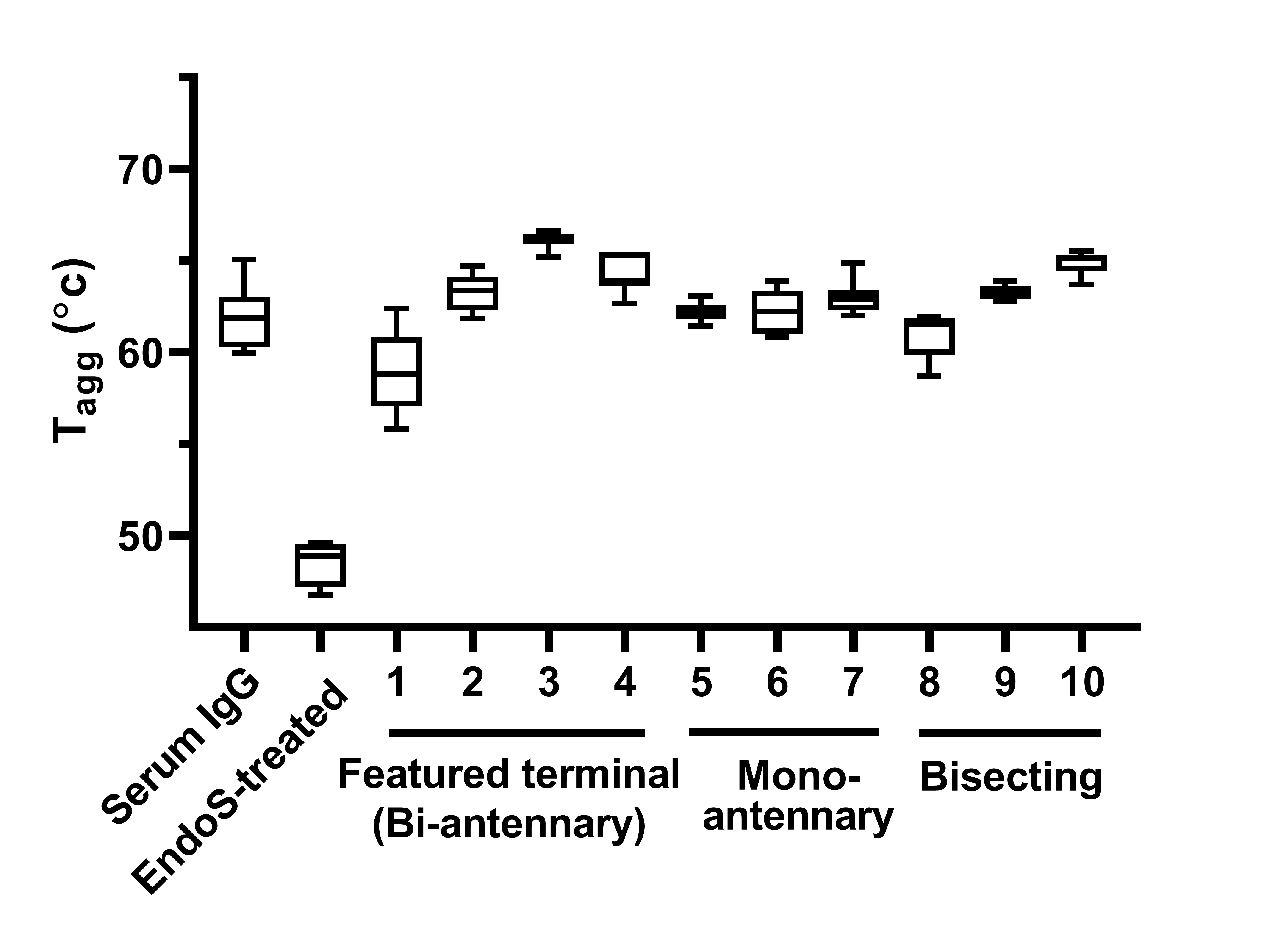

Supplement: Supplementary file 4 — Supplementary Data 1 [file 42003_2022_3257_MOESM4_ESM.zip › Source Data/Figure 5a-c/Figure 5c.png]

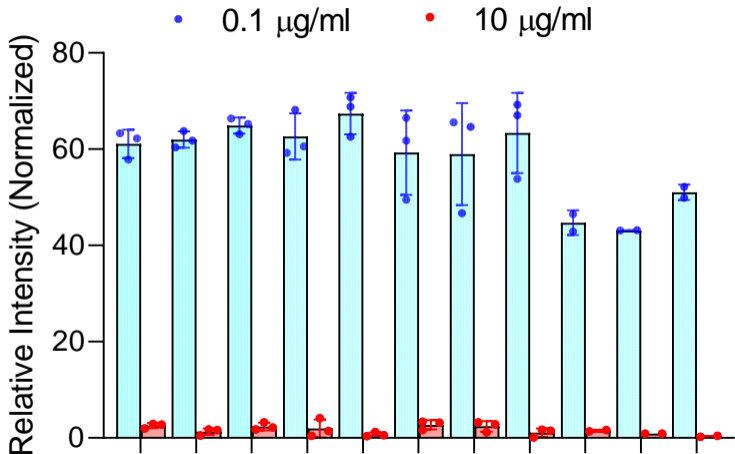

Supplement: Supplementary file 4 — Supplementary Data 1 [file 42003_2022_3257_MOESM4_ESM.zip › Source Data/Figure 5d/Figure 5d.pdf]

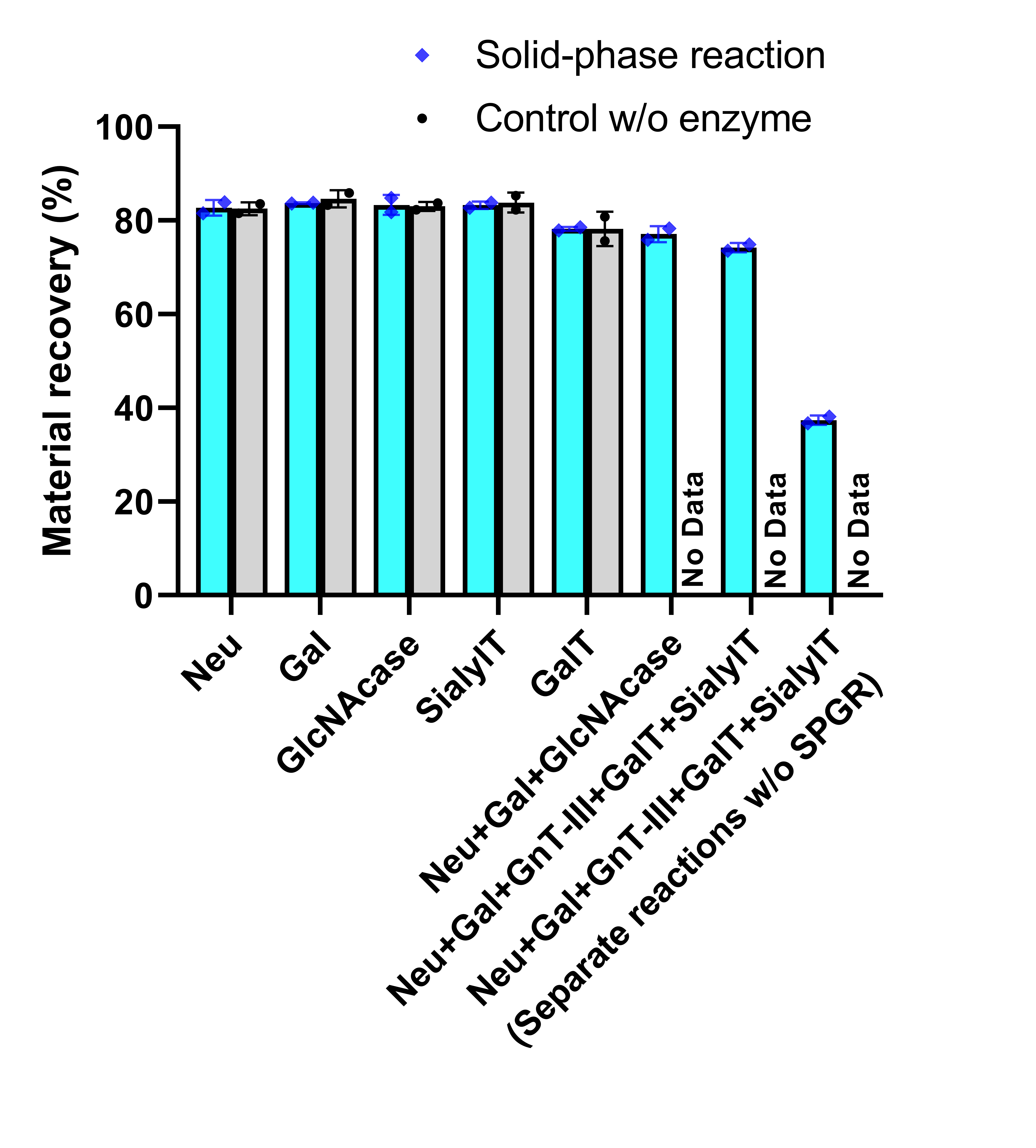

Supplement: Supplementary file 4 — Supplementary Data 1 [file 42003_2022_3257_MOESM4_ESM.zip › Source Data/Figure S13a/figure S13a.png]

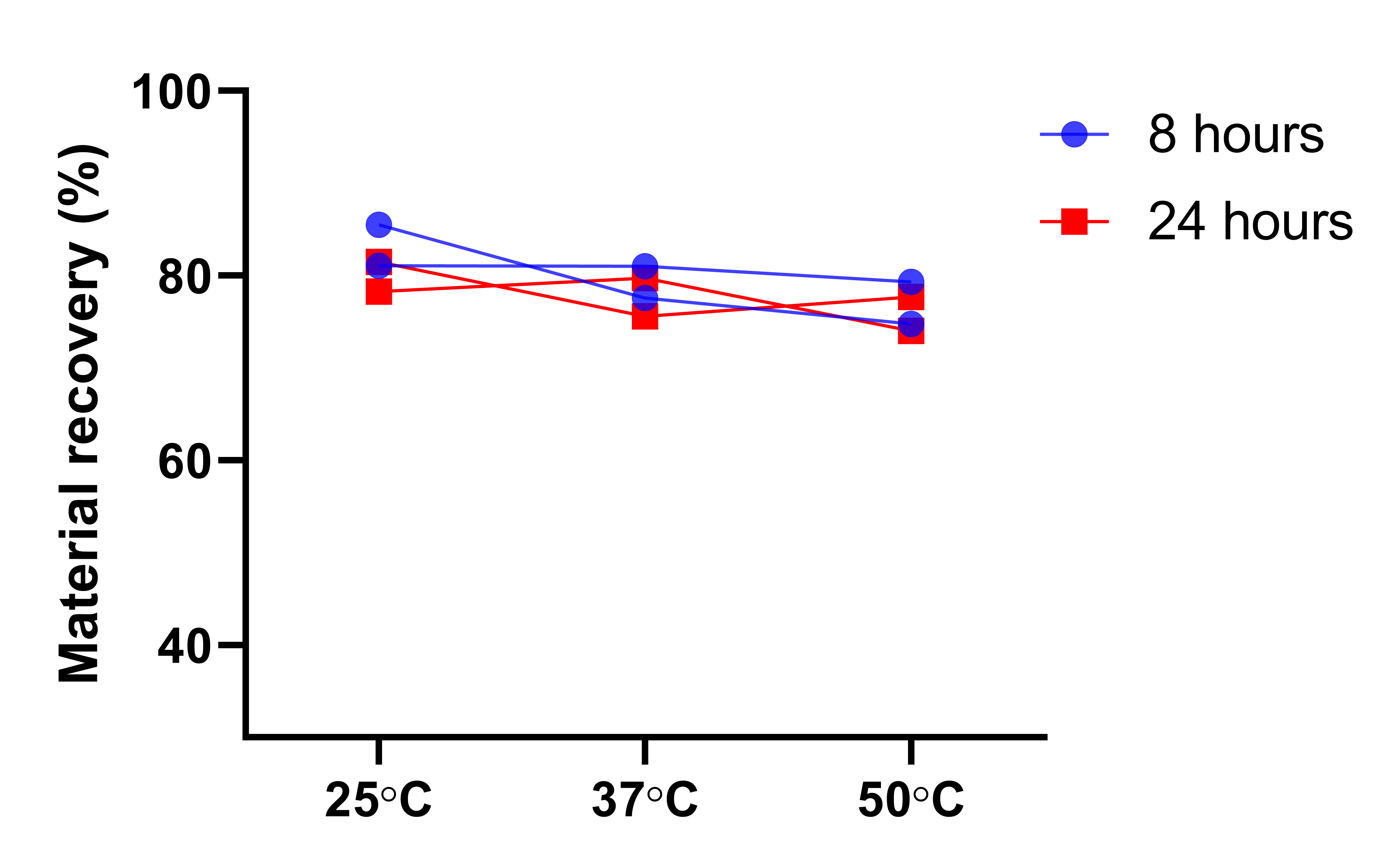

Supplement: Supplementary file 4 — Supplementary Data 1 [file 42003_2022_3257_MOESM4_ESM.zip › Source Data/Figure S13b/Figure S13b.png]

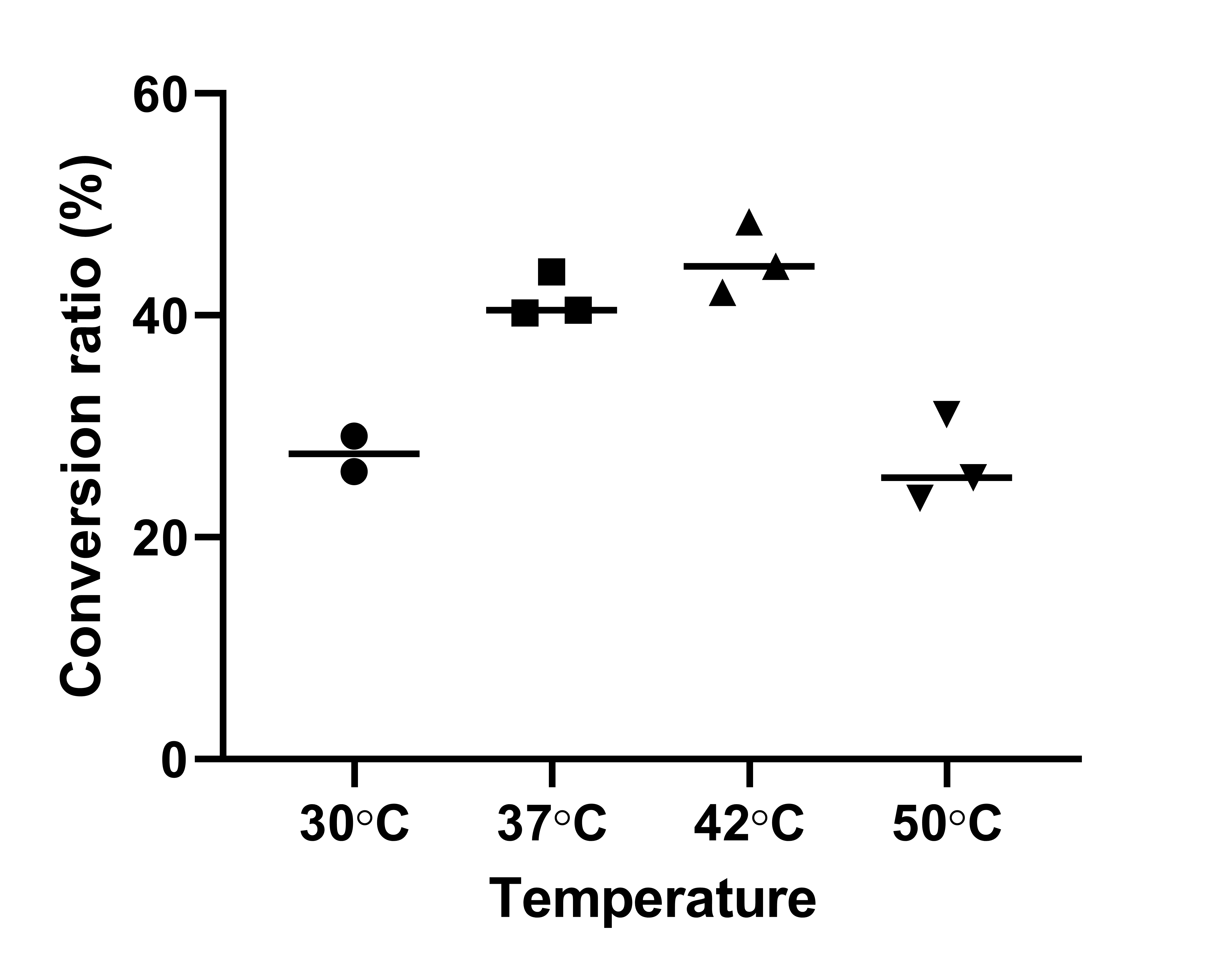

Supplement: Supplementary file 4 — Supplementary Data 1 [file 42003_2022_3257_MOESM4_ESM.zip › Source Data/Figure S16b/Figure S16b.png]

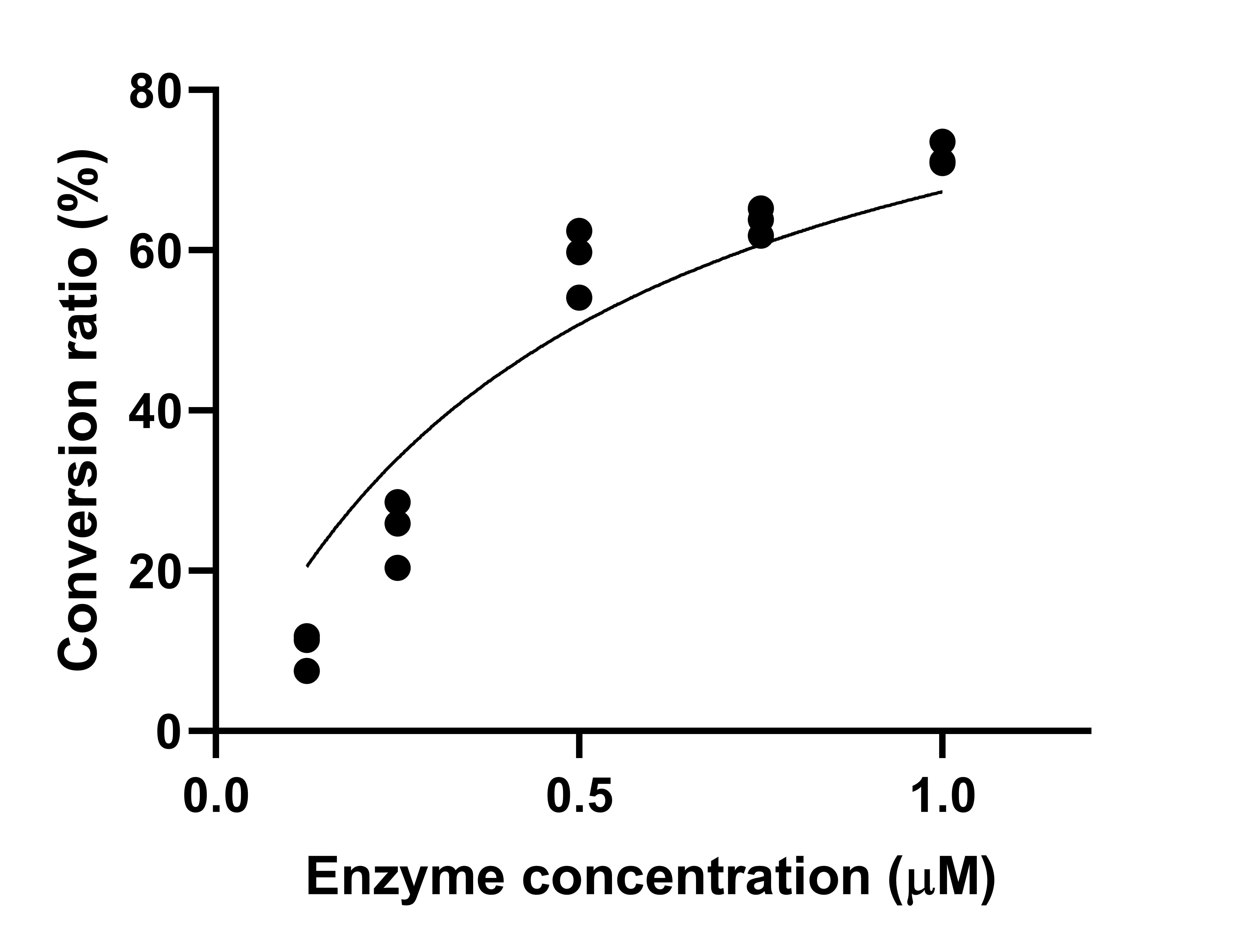

Supplement: Supplementary file 4 — Supplementary Data 1 [file 42003_2022_3257_MOESM4_ESM.zip › Source Data/Figure S16c/Figure S16c.png]

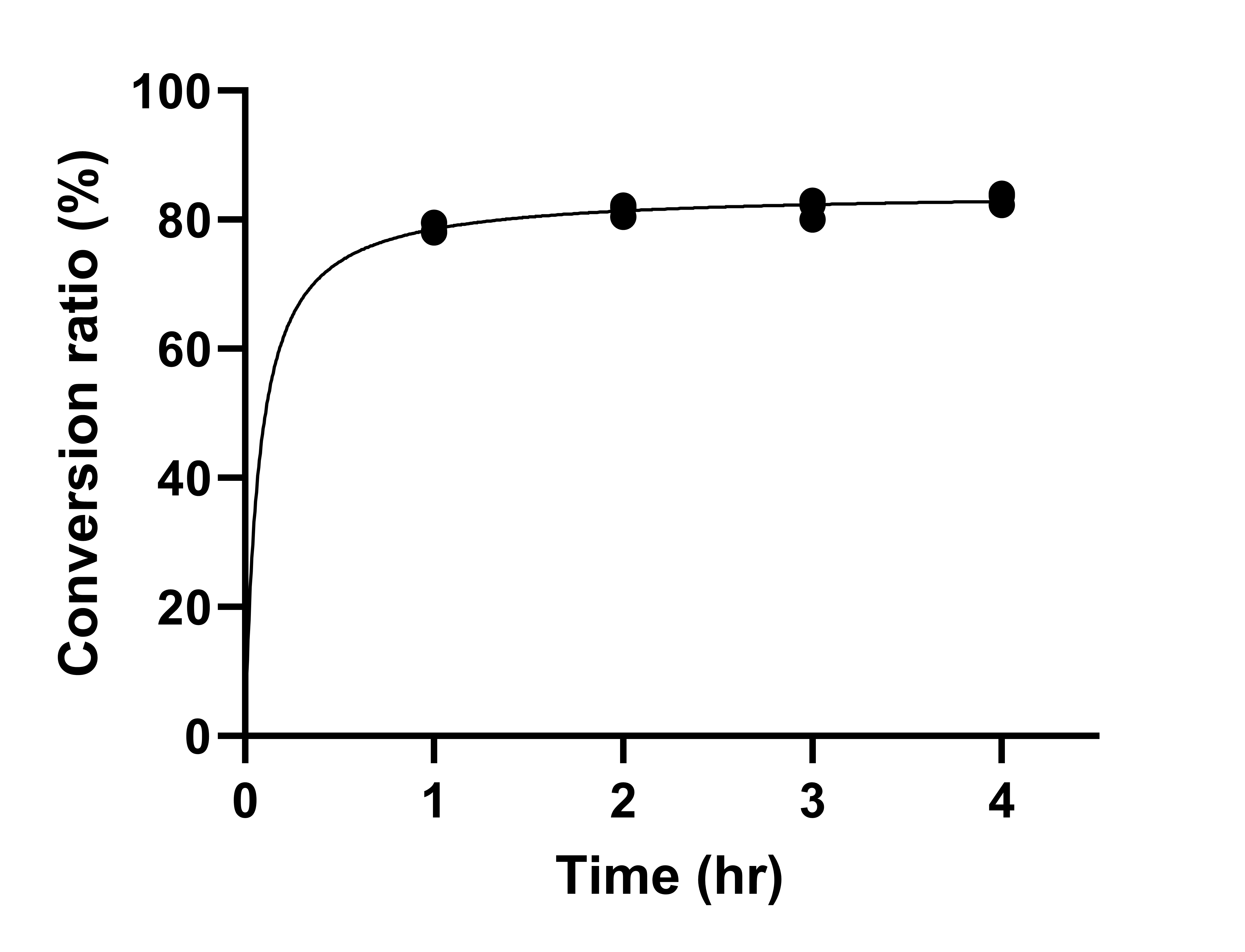

Supplement: Supplementary file 4 — Supplementary Data 1 [file 42003_2022_3257_MOESM4_ESM.zip › Source Data/Figure S16d/Figure S16d.png]

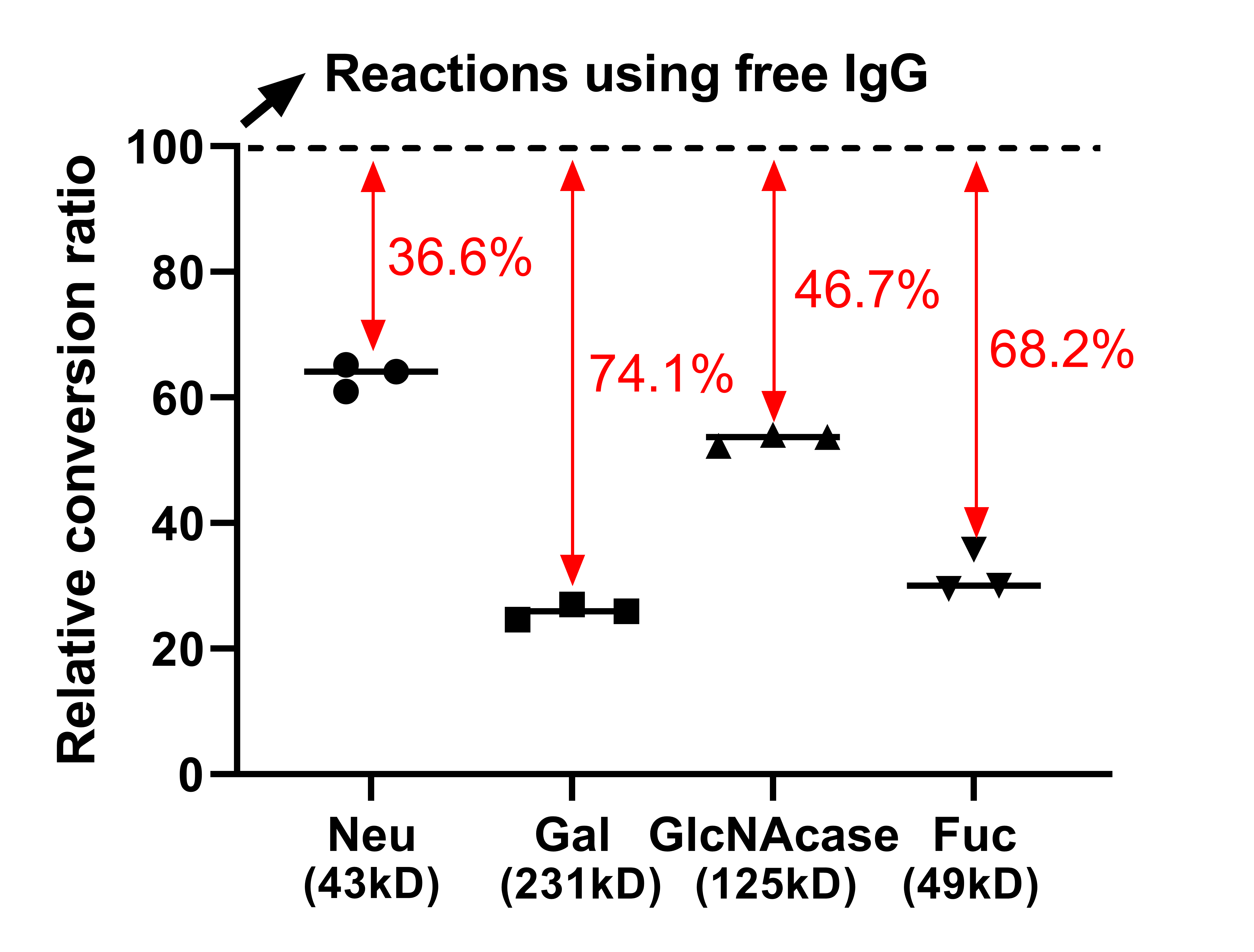

Supplement: Supplementary file 4 — Supplementary Data 1 [file 42003_2022_3257_MOESM4_ESM.zip › Source Data/Figure S17a/Figure S17a.png]

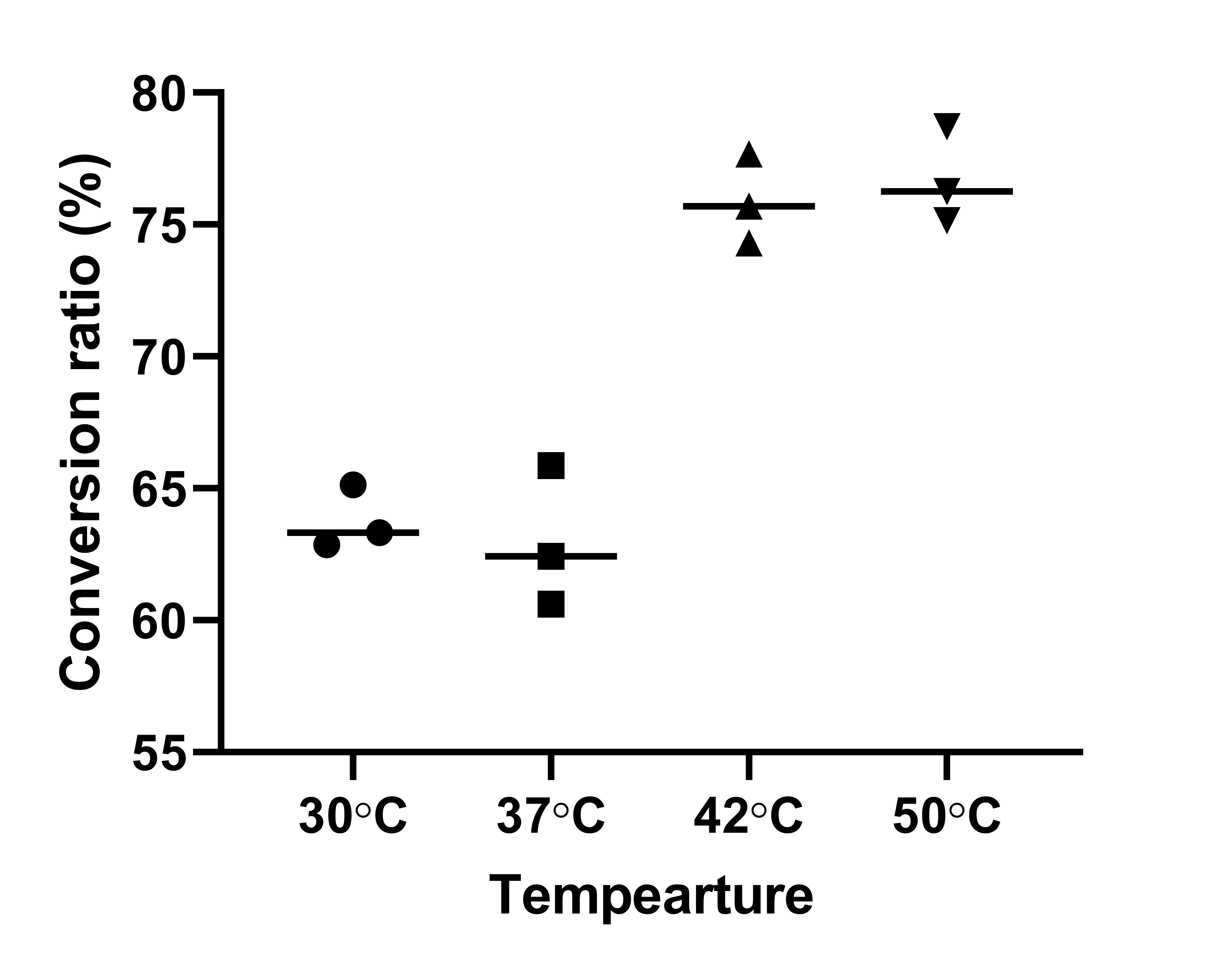

Supplement: Supplementary file 4 — Supplementary Data 1 [file 42003_2022_3257_MOESM4_ESM.zip › Source Data/Figure S1a/Figure S1a.png]

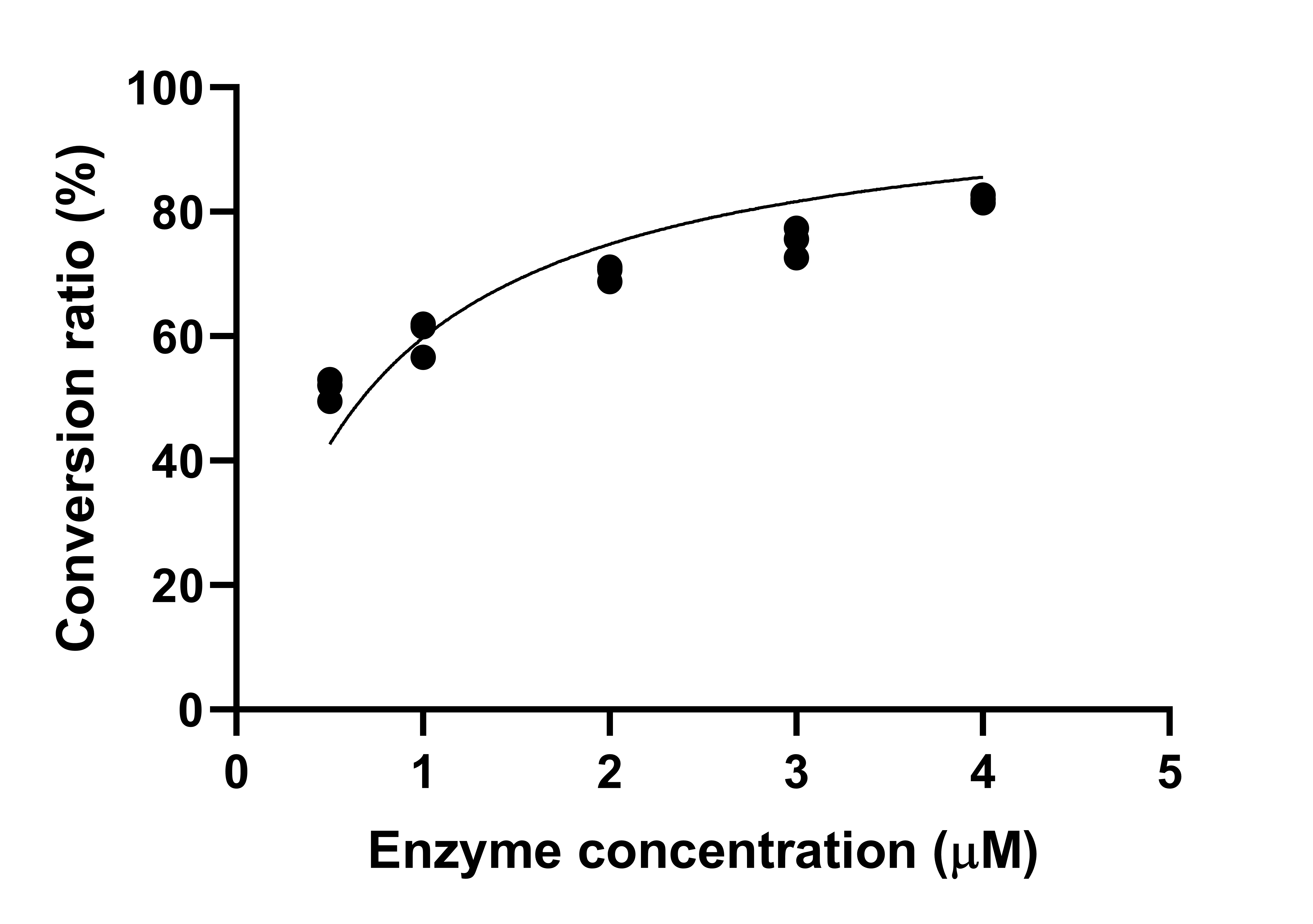

Supplement: Supplementary file 4 — Supplementary Data 1 [file 42003_2022_3257_MOESM4_ESM.zip › Source Data/Figure S1b/Figure S1b.png]

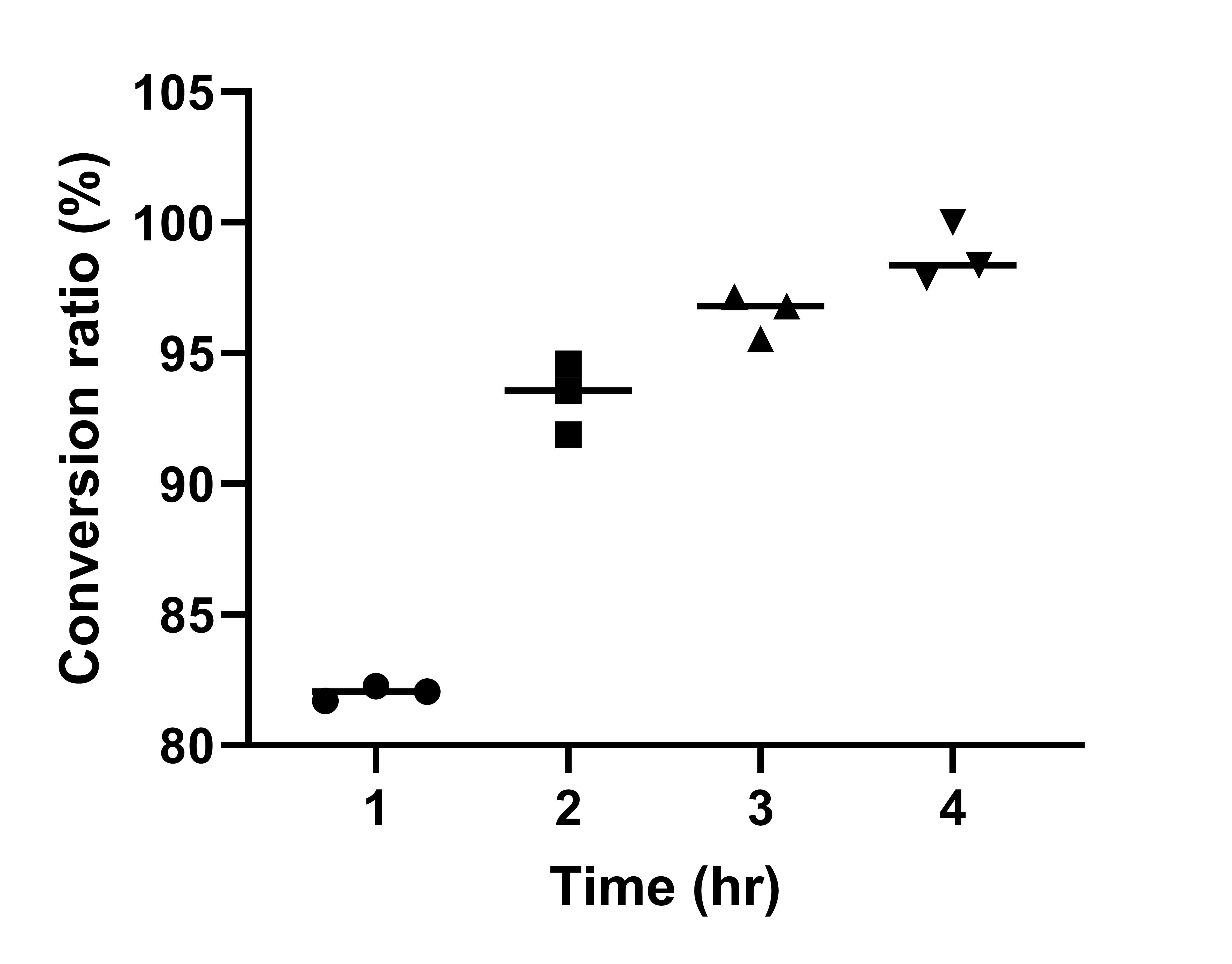

Supplement: Supplementary file 4 — Supplementary Data 1 [file 42003_2022_3257_MOESM4_ESM.zip › Source Data/Figure S1c/Figure S1c.png]

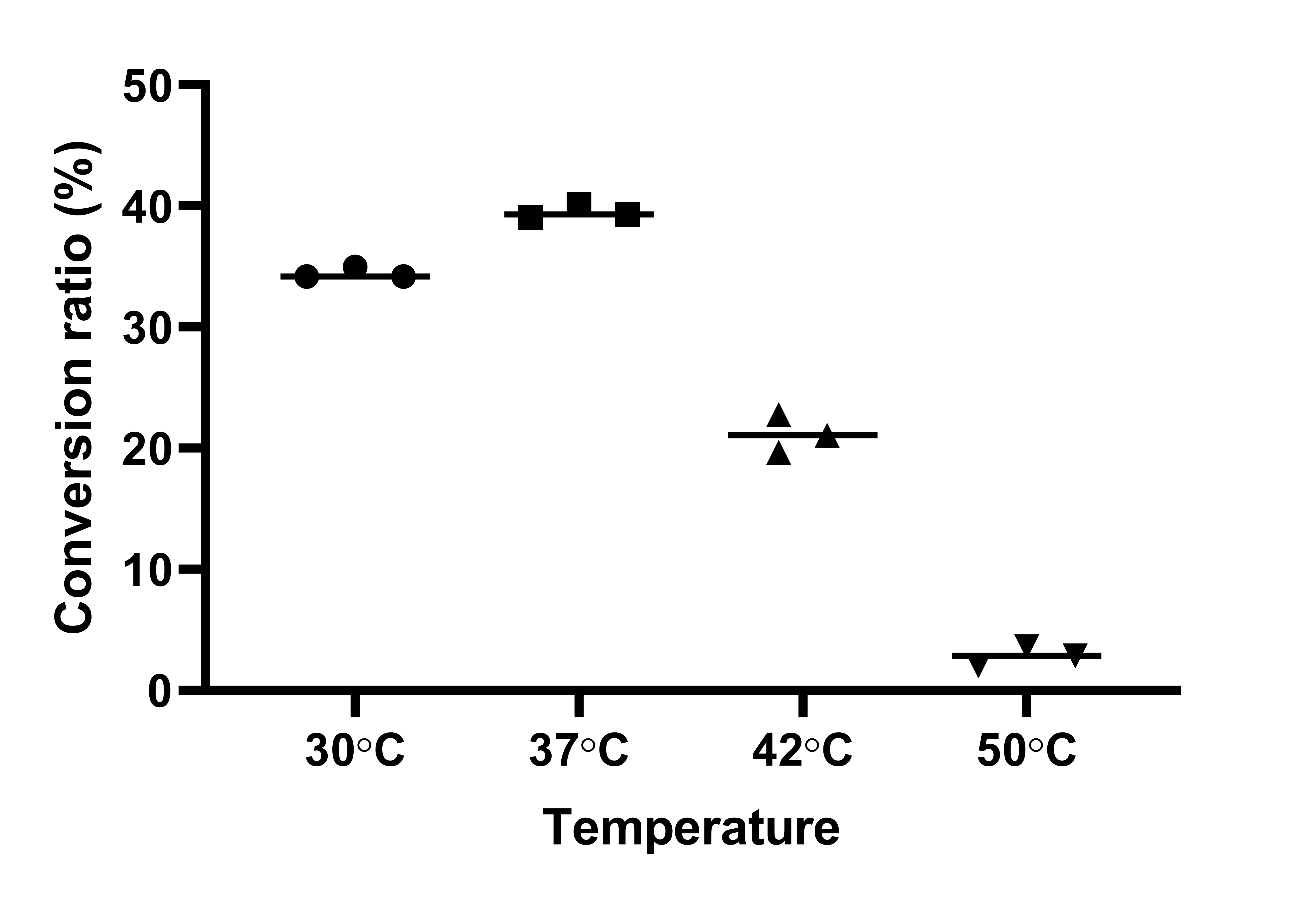

Supplement: Supplementary file 4 — Supplementary Data 1 [file 42003_2022_3257_MOESM4_ESM.zip › Source Data/Figure S2a/Figure S2a.png]

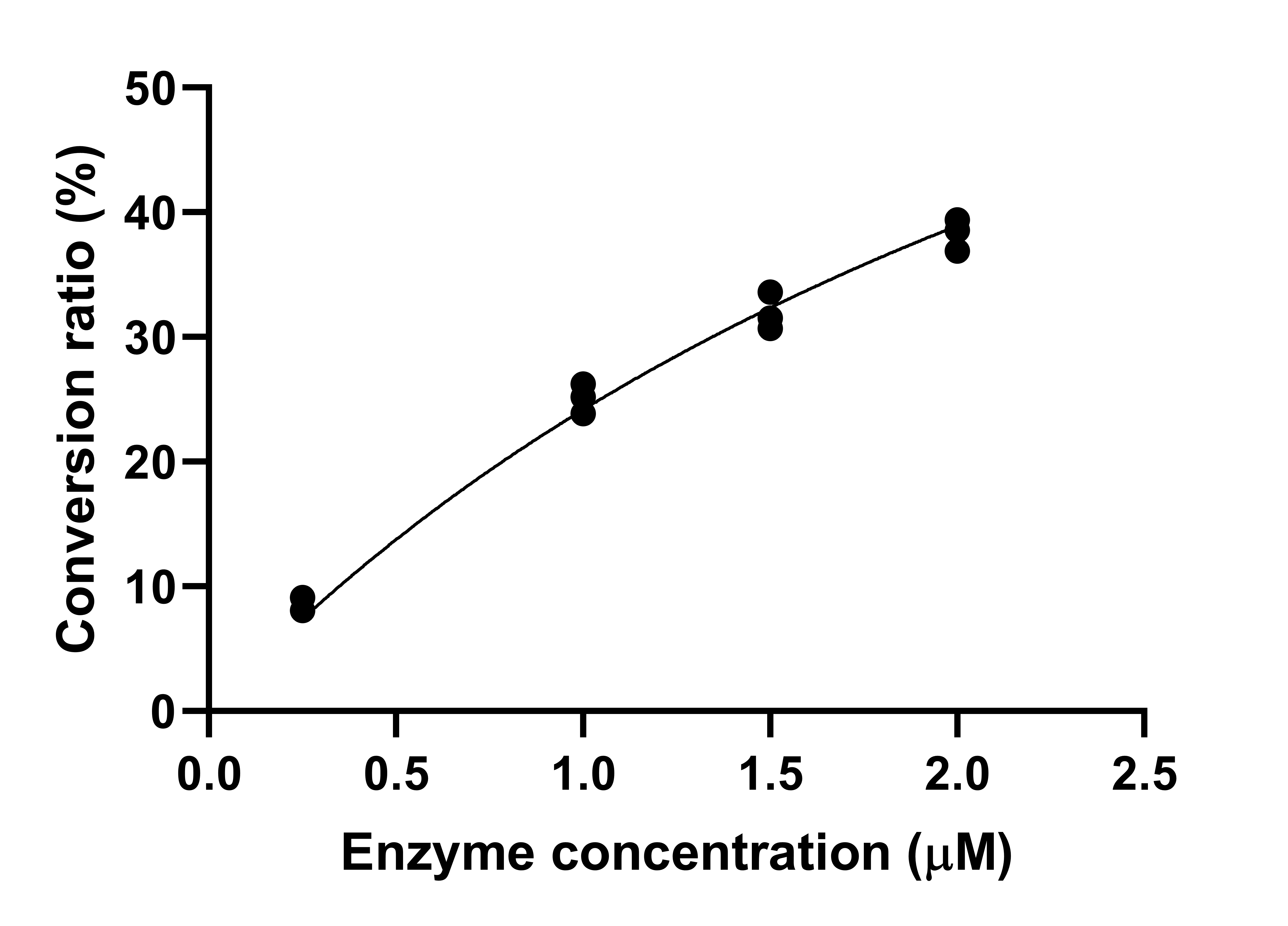

Supplement: Supplementary file 4 — Supplementary Data 1 [file 42003_2022_3257_MOESM4_ESM.zip › Source Data/Figure S2b/Figure S2b.png]

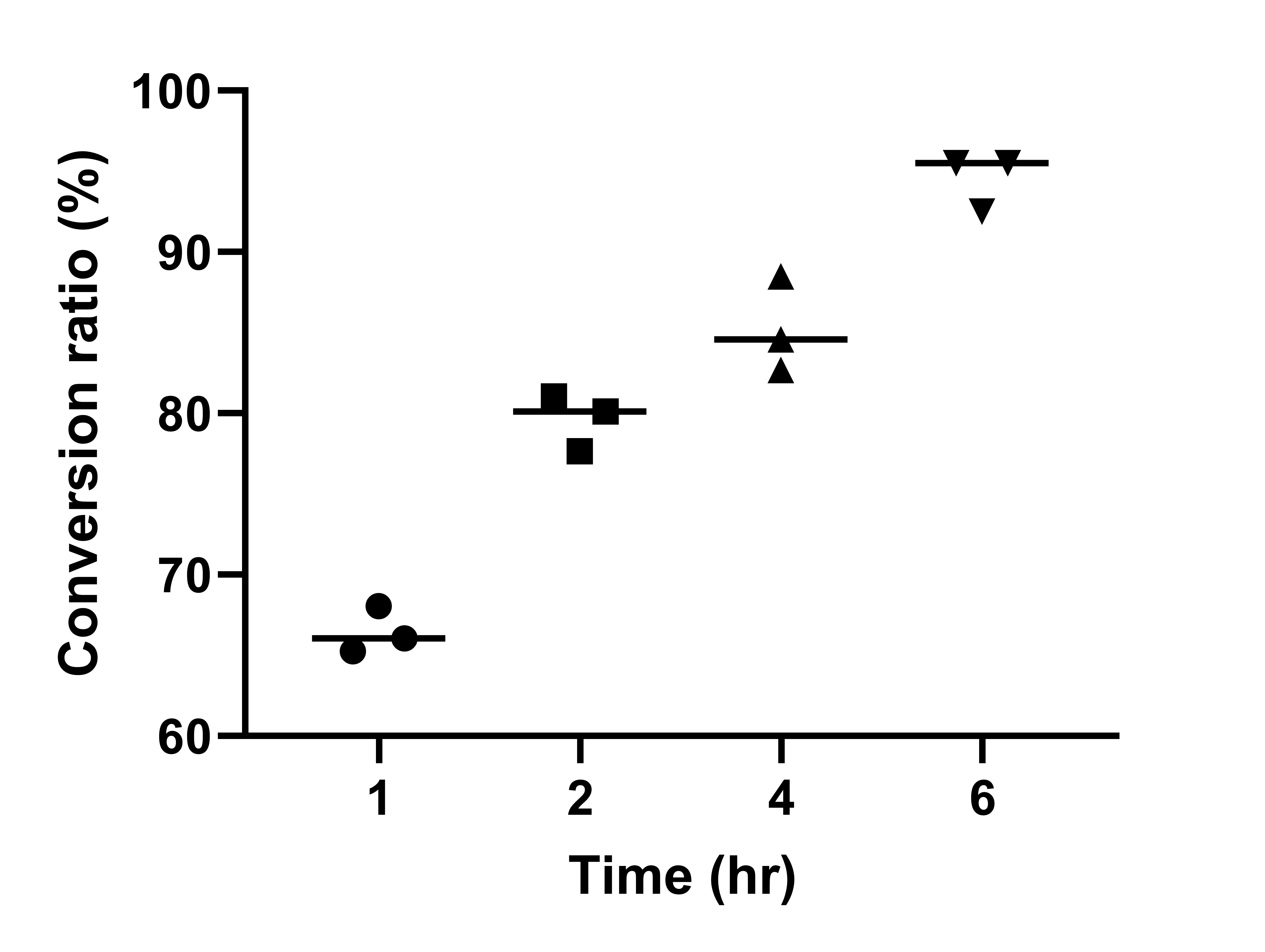

Supplement: Supplementary file 4 — Supplementary Data 1 [file 42003_2022_3257_MOESM4_ESM.zip › Source Data/Figure S2c/Figure S2c.png]

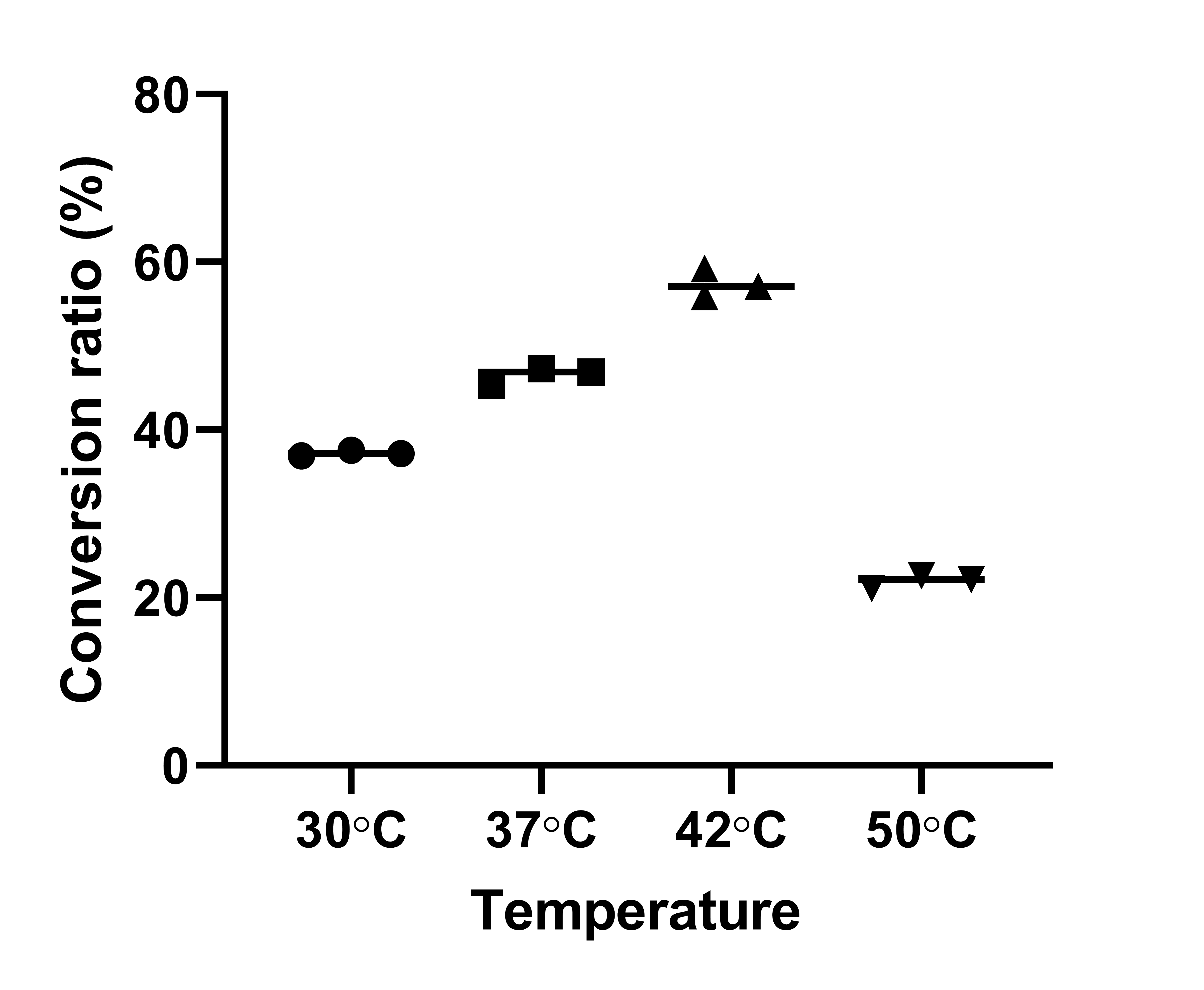

Supplement: Supplementary file 4 — Supplementary Data 1 [file 42003_2022_3257_MOESM4_ESM.zip › Source Data/Figure S3a/Figure S3a.png]

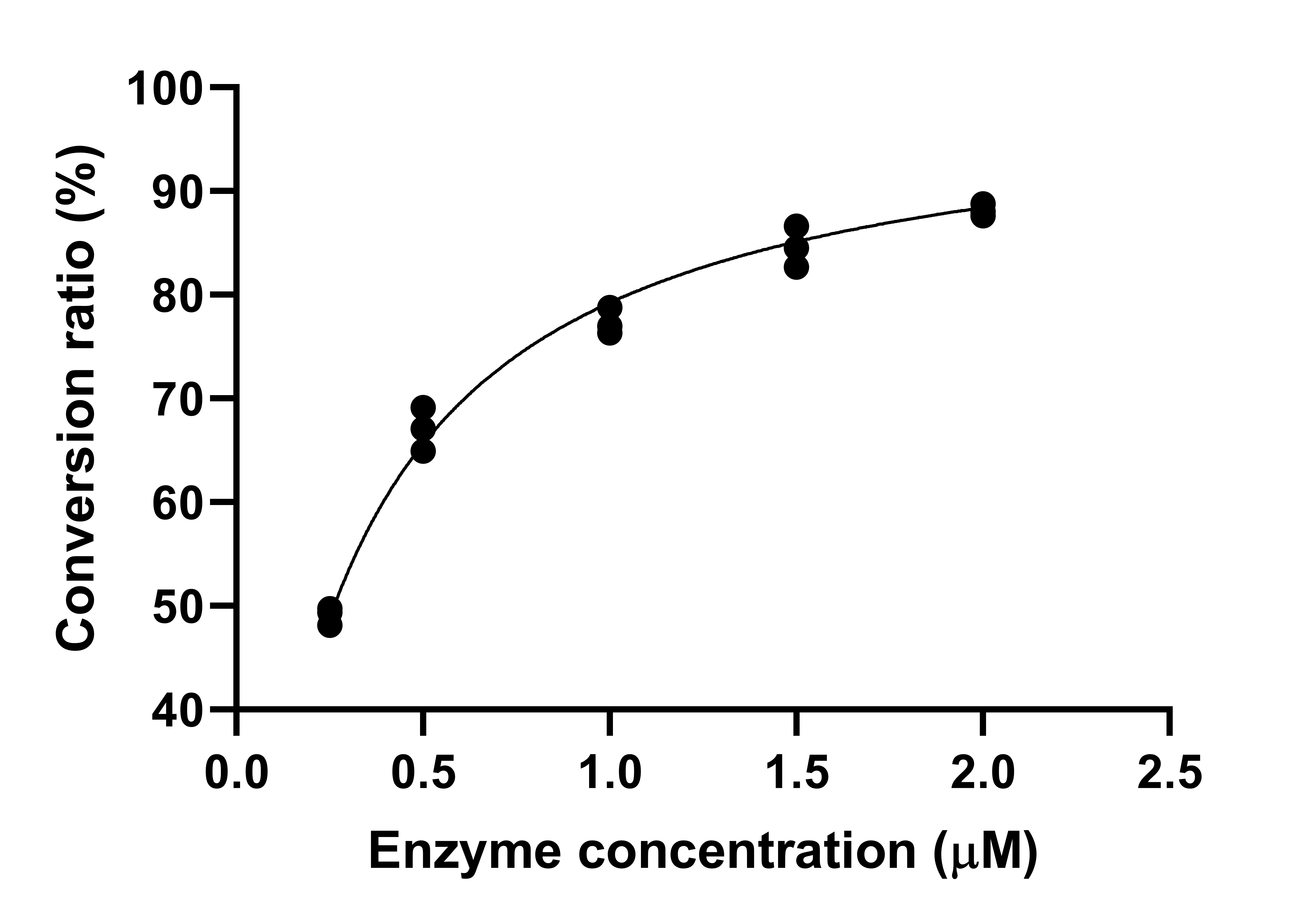

Supplement: Supplementary file 4 — Supplementary Data 1 [file 42003_2022_3257_MOESM4_ESM.zip › Source Data/Figure S3b/Figure S3b.png]

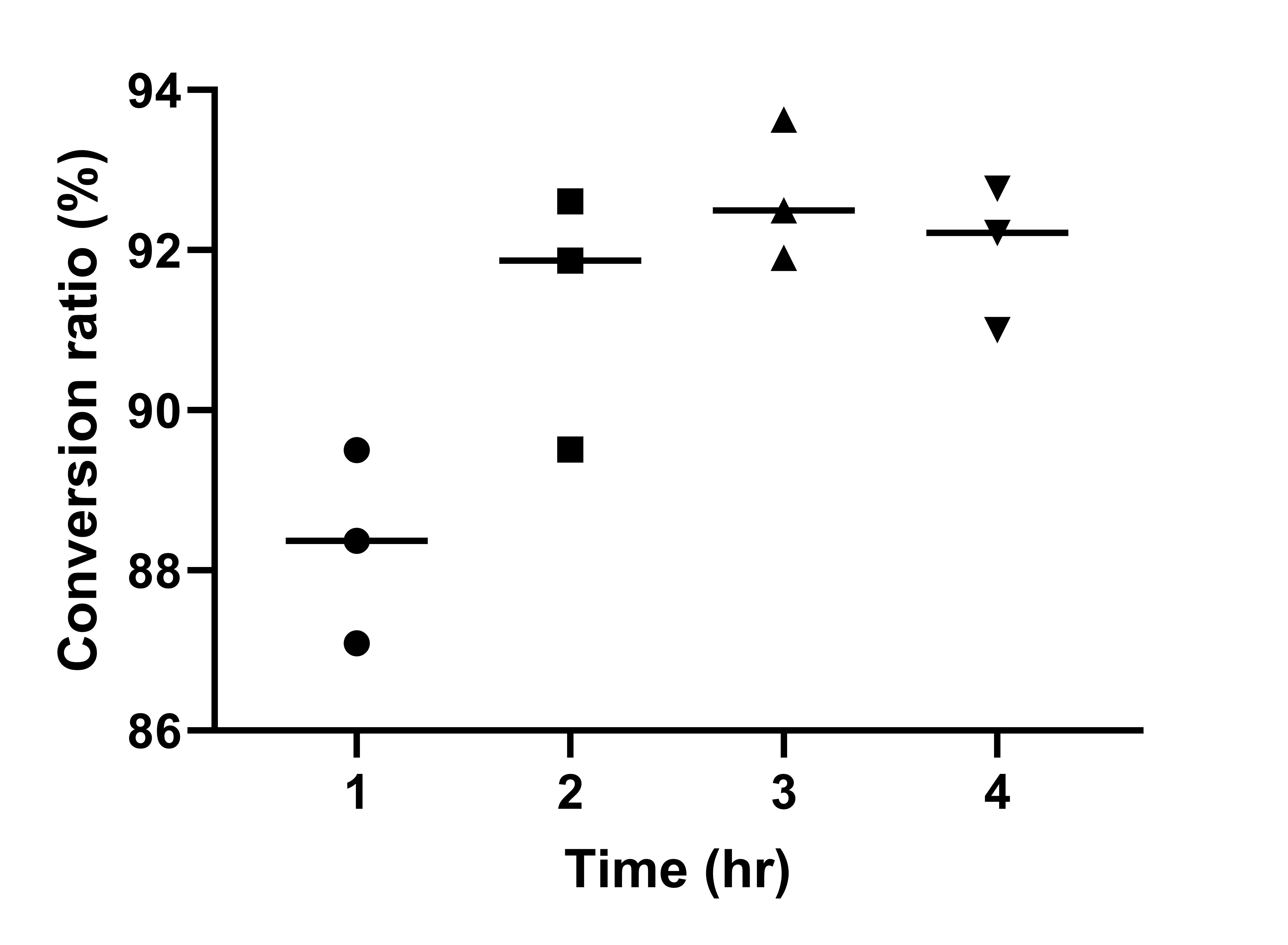

Supplement: Supplementary file 4 — Supplementary Data 1 [file 42003_2022_3257_MOESM4_ESM.zip › Source Data/Figure S3c/Figure S3c.png]

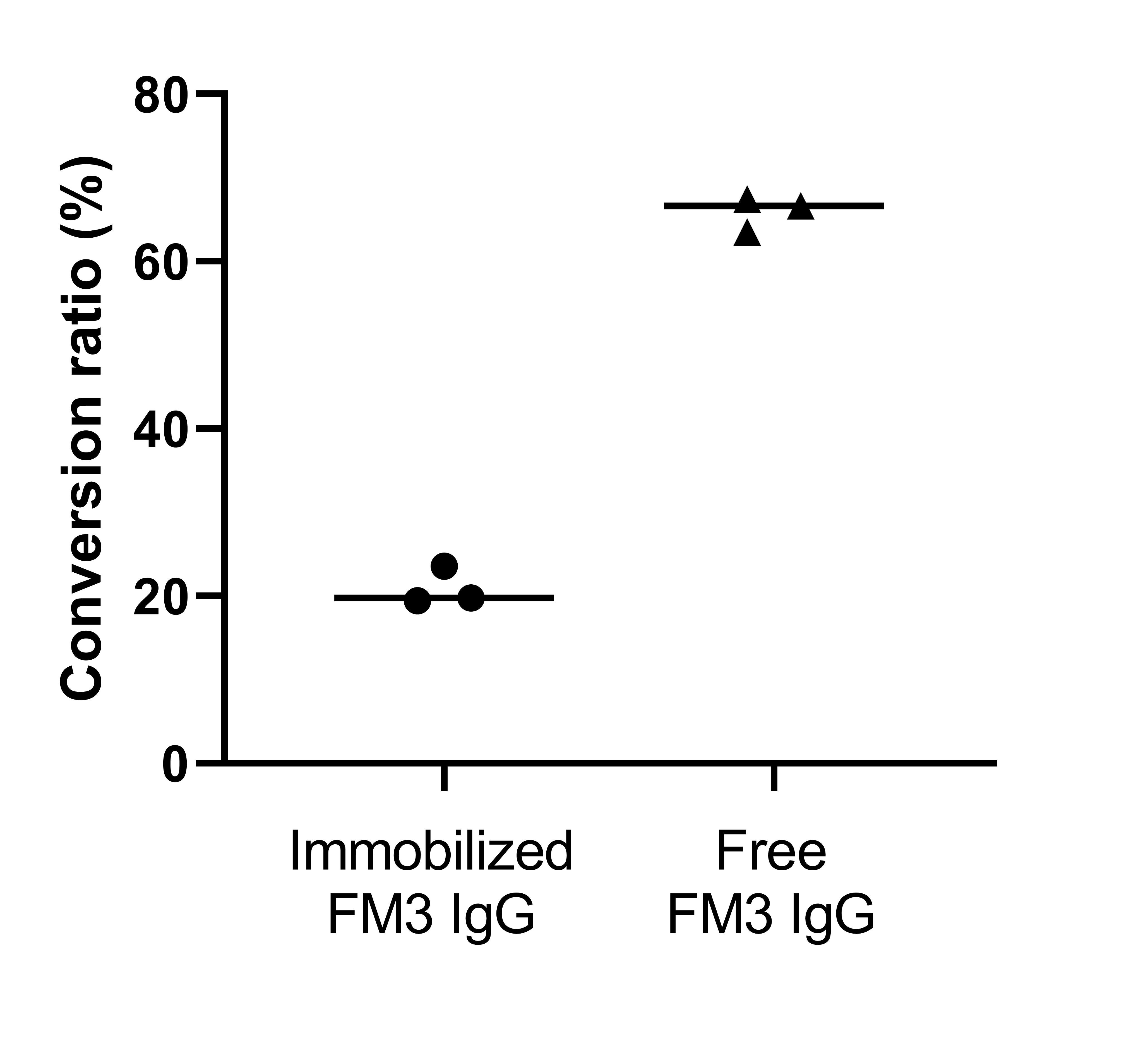

Supplement: Supplementary file 4 — Supplementary Data 1 [file 42003_2022_3257_MOESM4_ESM.zip › Source Data/Figure S4b/Figure S4a.png]

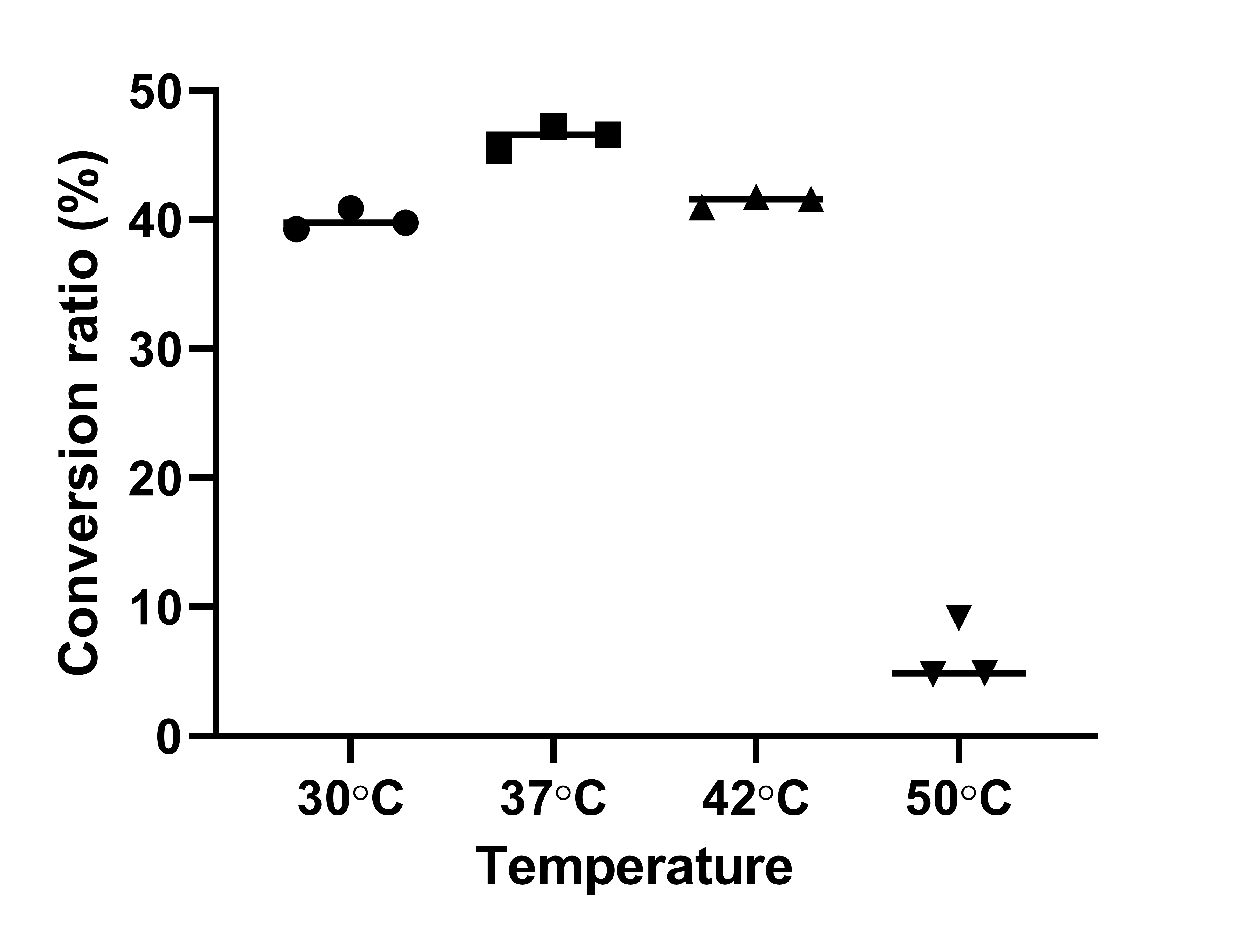

Supplement: Supplementary file 4 — Supplementary Data 1 [file 42003_2022_3257_MOESM4_ESM.zip › Source Data/Figure S5a/Figure S5a.png]

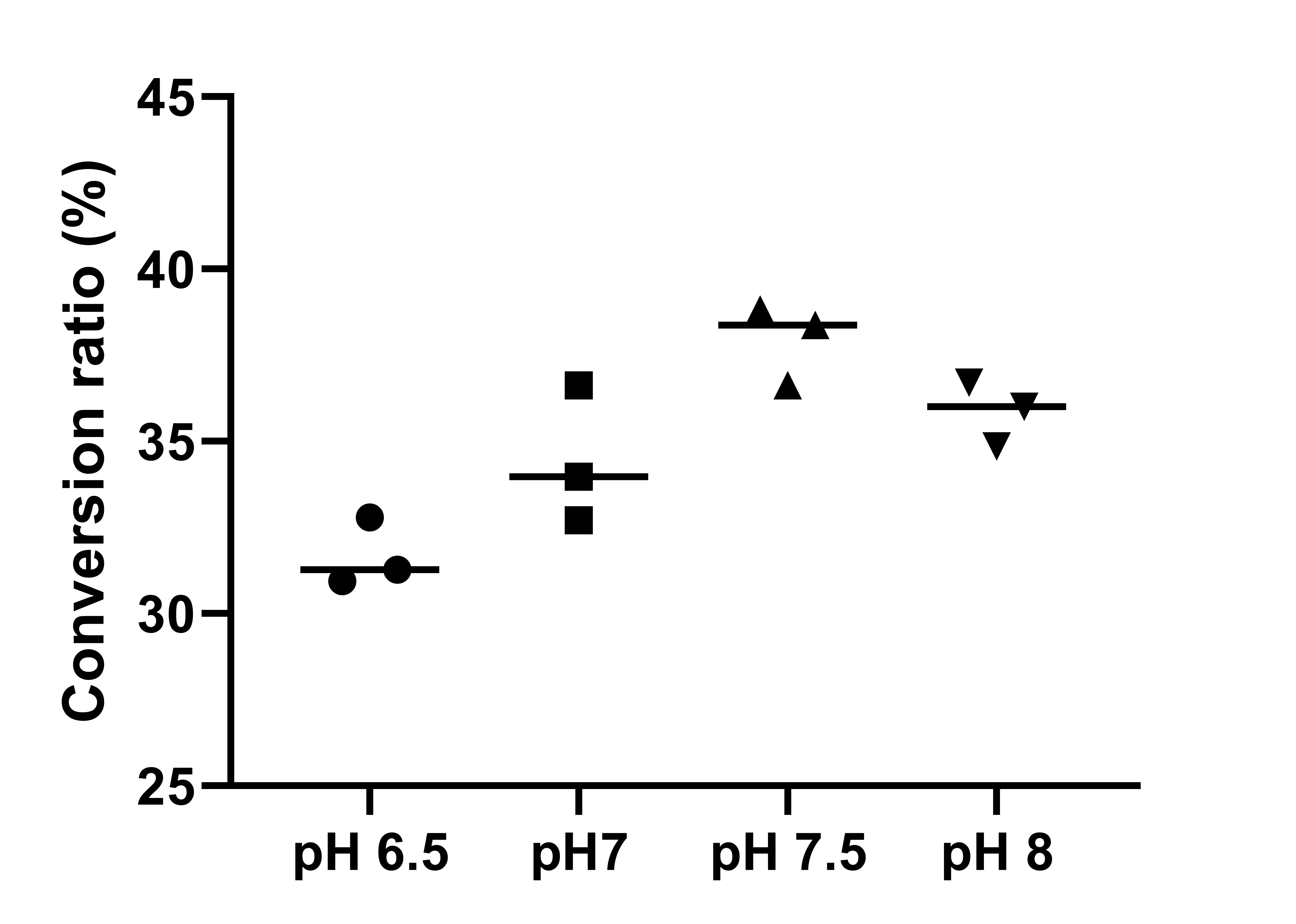

Supplement: Supplementary file 4 — Supplementary Data 1 [file 42003_2022_3257_MOESM4_ESM.zip › Source Data/Figure S5b/Figure S5b.png]

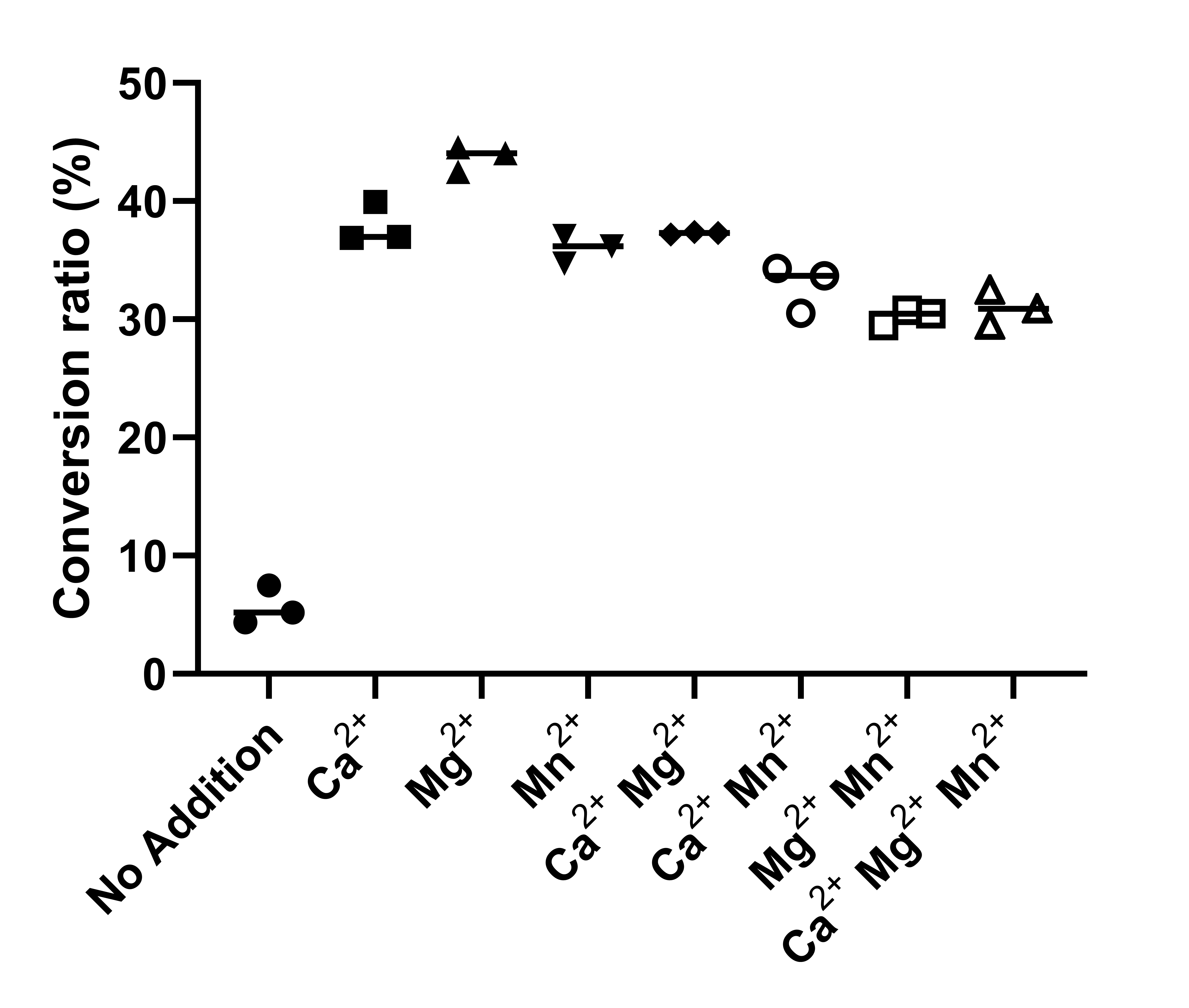

Supplement: Supplementary file 4 — Supplementary Data 1 [file 42003_2022_3257_MOESM4_ESM.zip › Source Data/Figure S5c/Figure S5c.png]

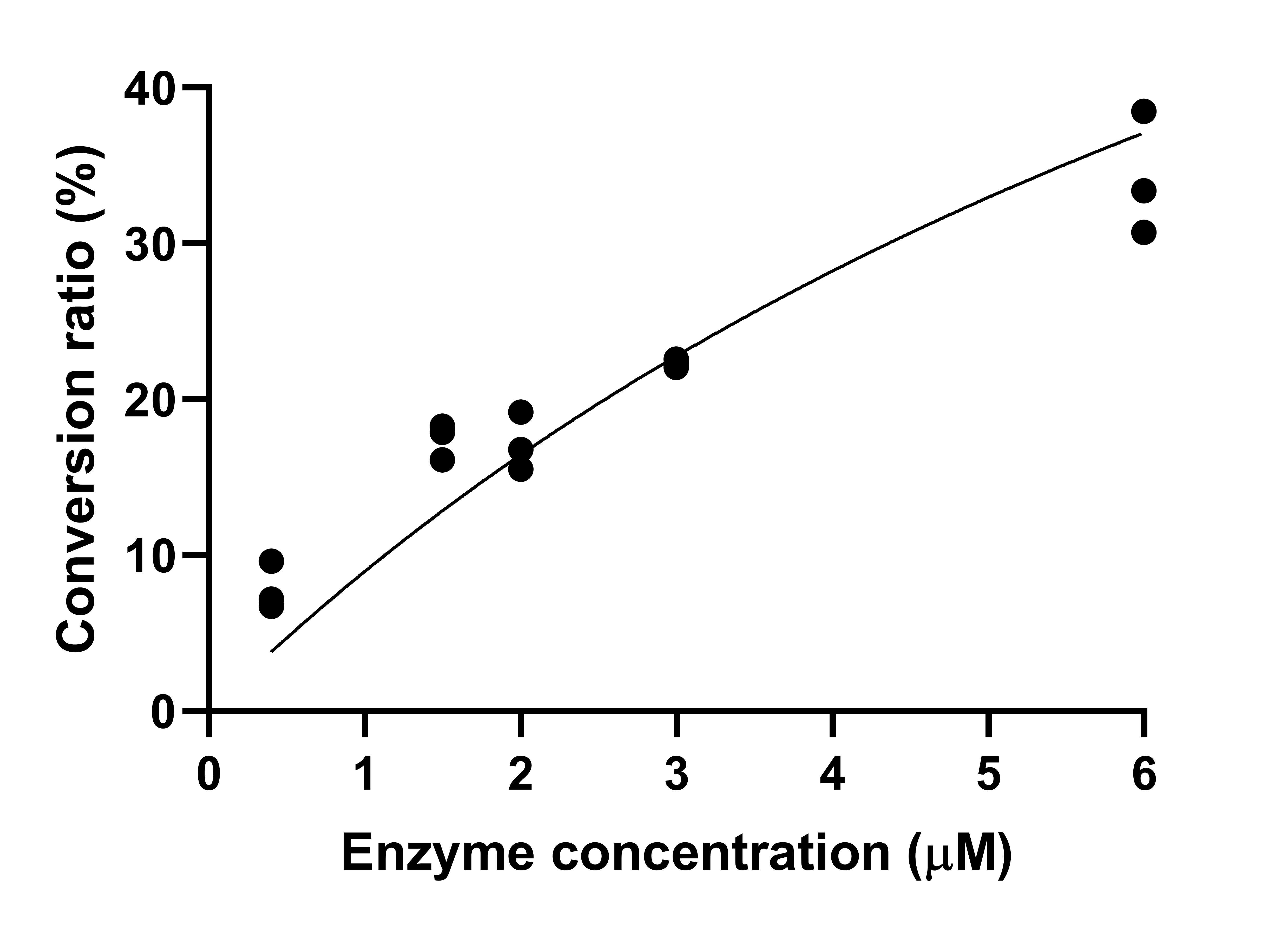

Supplement: Supplementary file 4 — Supplementary Data 1 [file 42003_2022_3257_MOESM4_ESM.zip › Source Data/Figure S5d/Figure S5d.png]

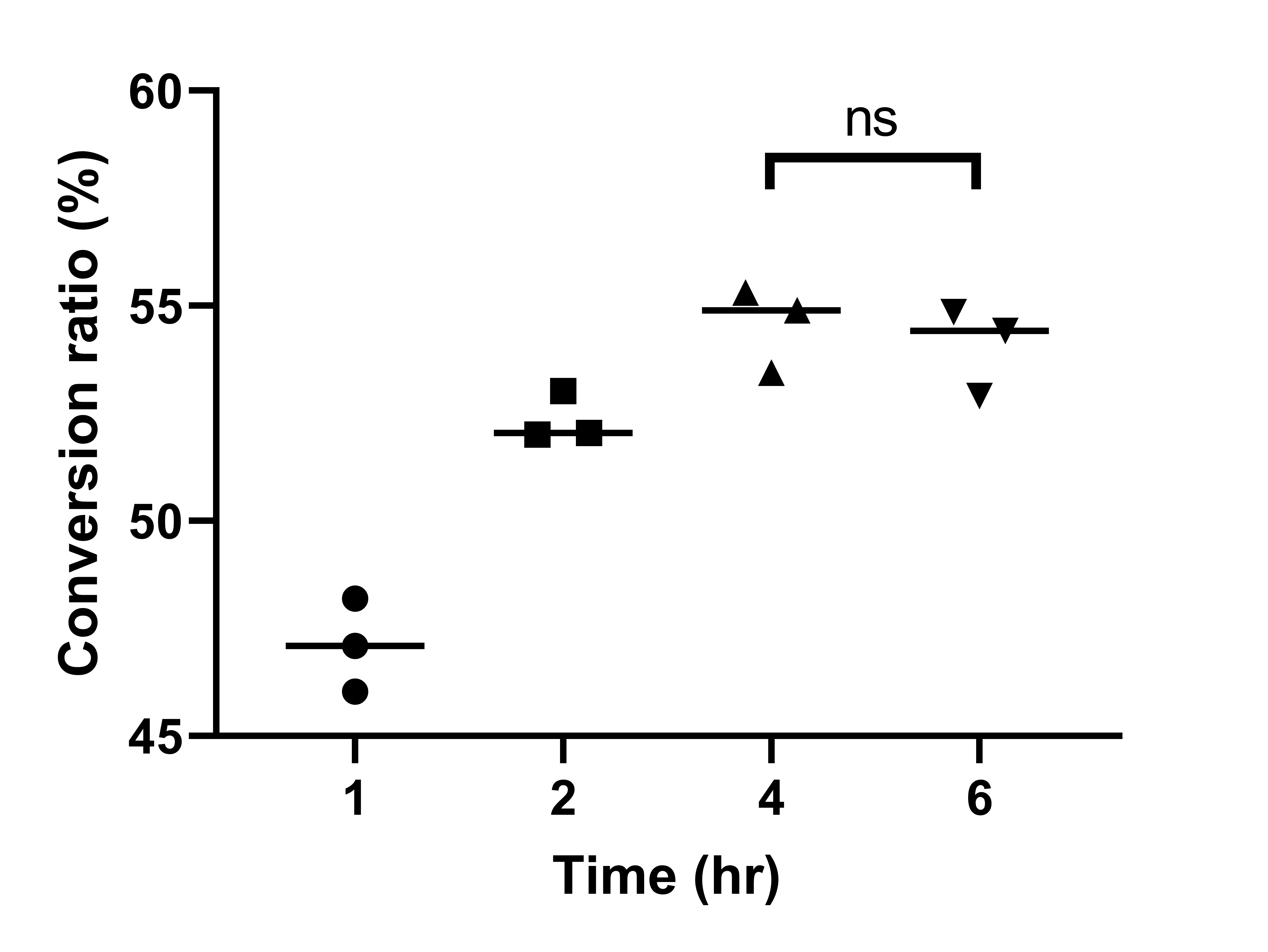

Supplement: Supplementary file 4 — Supplementary Data 1 [file 42003_2022_3257_MOESM4_ESM.zip › Source Data/Figure S5e-g/Figure S5e.png]

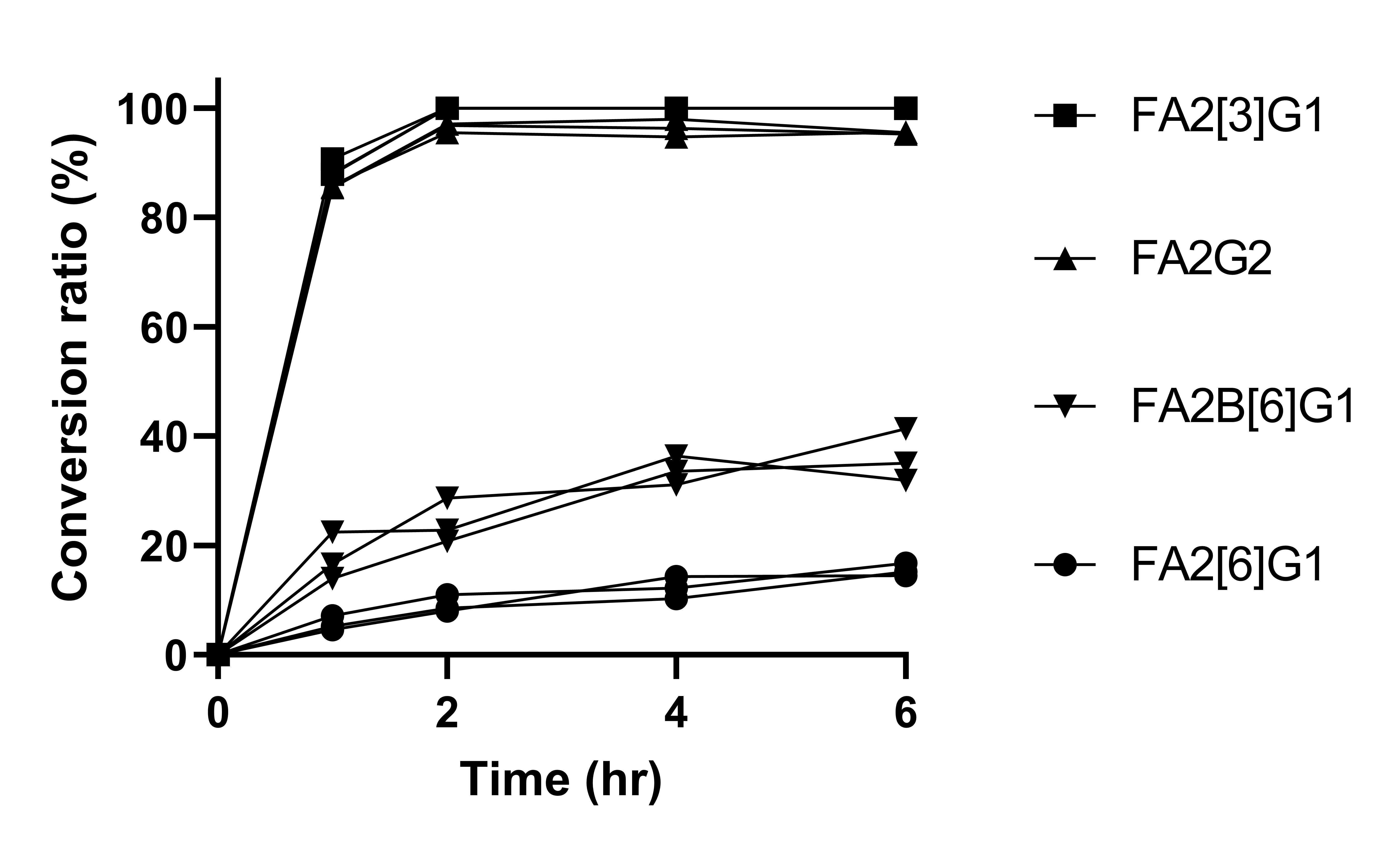

Supplement: Supplementary file 4 — Supplementary Data 1 [file 42003_2022_3257_MOESM4_ESM.zip › Source Data/Figure S5e-g/Figure S5f.png]

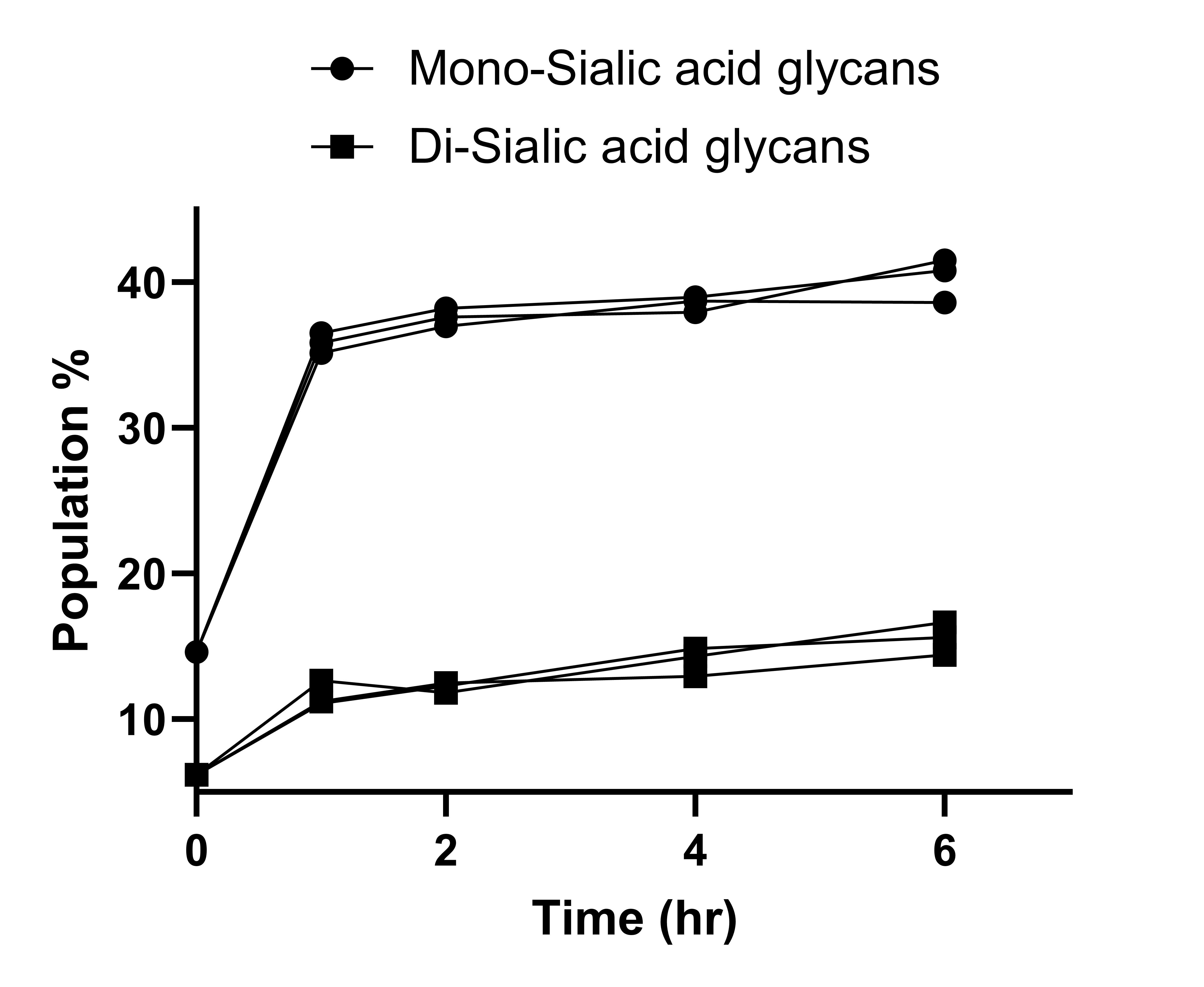

Supplement: Supplementary file 4 — Supplementary Data 1 [file 42003_2022_3257_MOESM4_ESM.zip › Source Data/Figure S5e-g/Figure S5g.png]

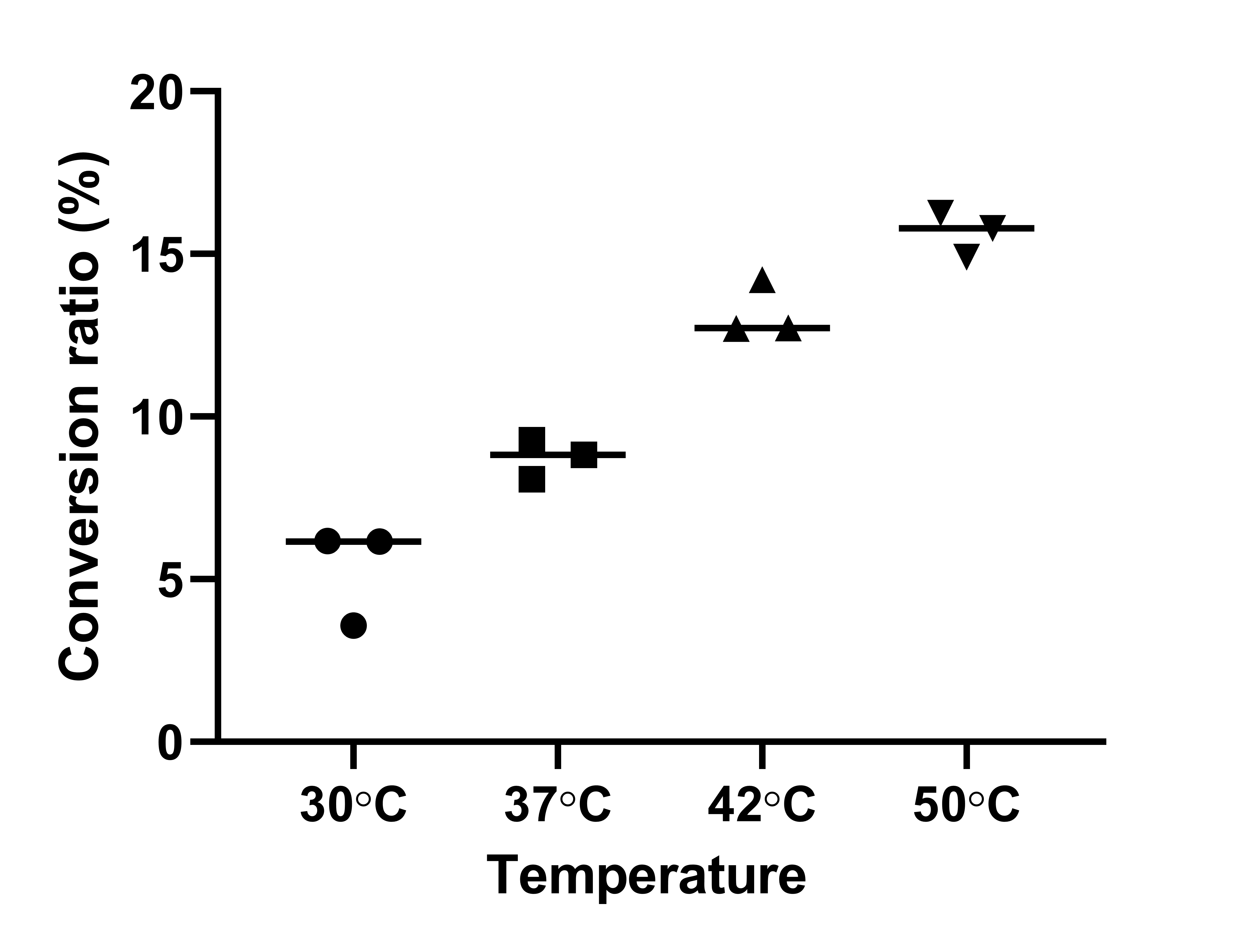

Supplement: Supplementary file 4 — Supplementary Data 1 [file 42003_2022_3257_MOESM4_ESM.zip › Source Data/Figure S6a/Figure S6a.png]

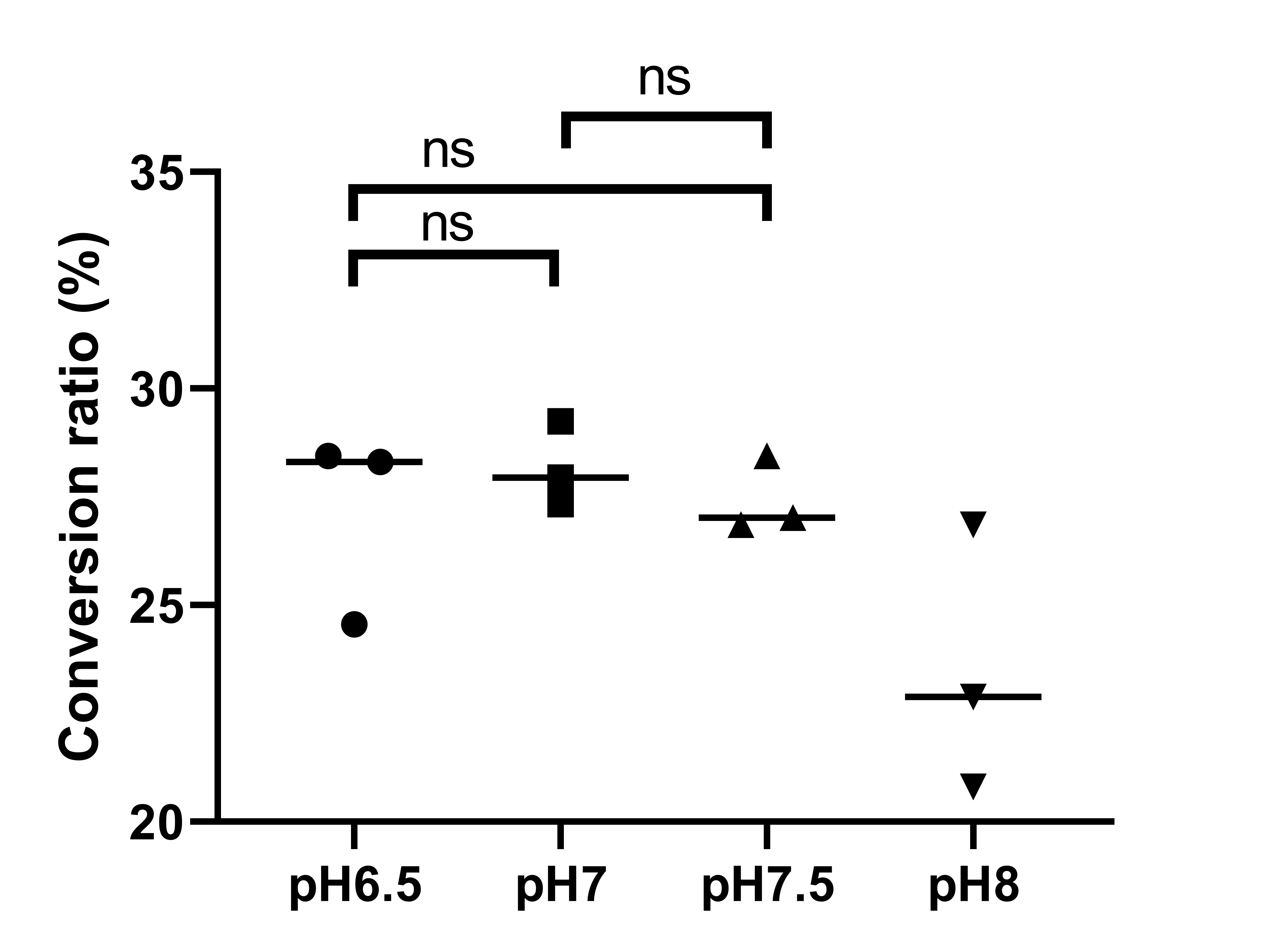

Supplement: Supplementary file 4 — Supplementary Data 1 [file 42003_2022_3257_MOESM4_ESM.zip › Source Data/Figure S6b/Figure S6b.png]

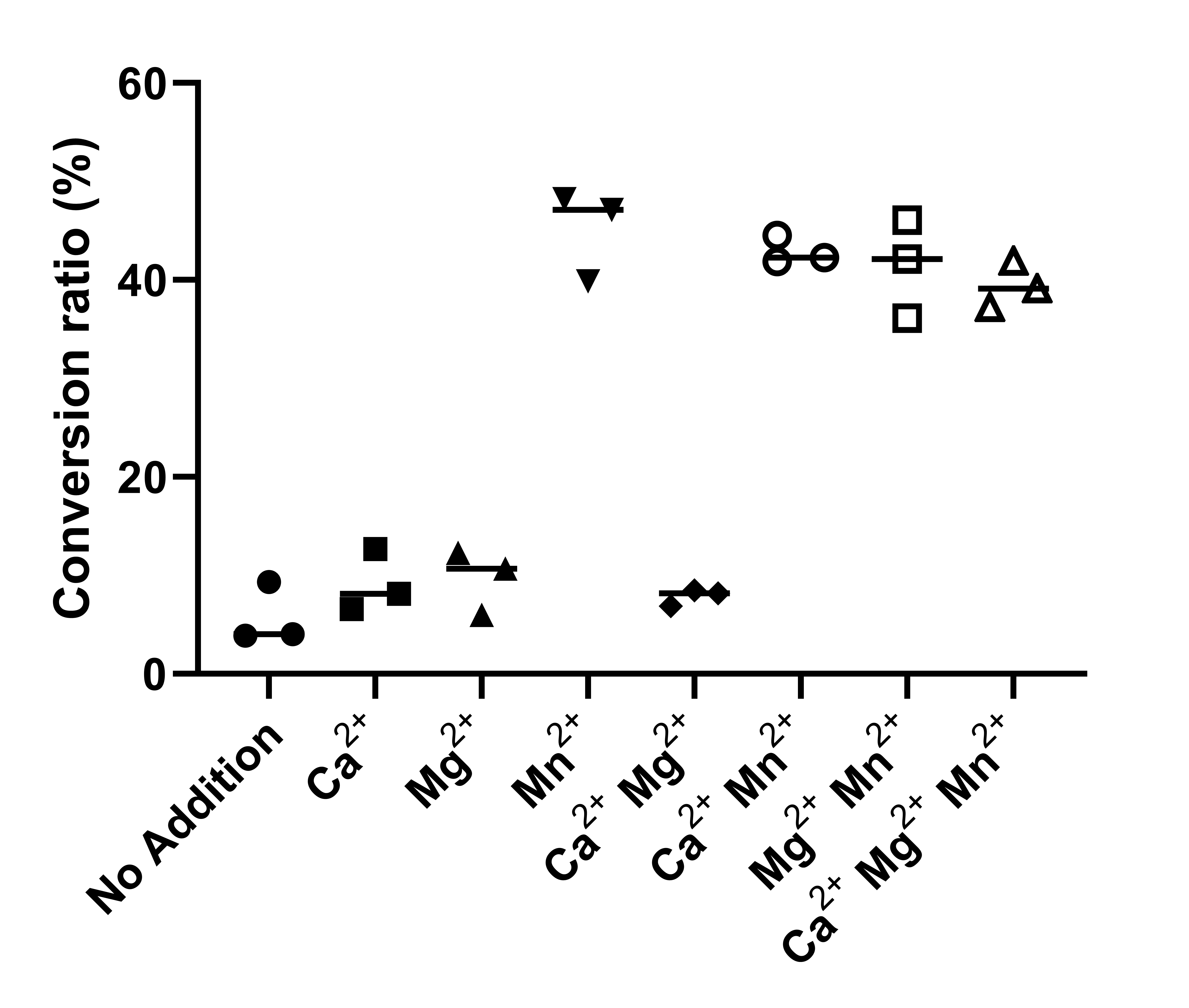

Supplement: Supplementary file 4 — Supplementary Data 1 [file 42003_2022_3257_MOESM4_ESM.zip › Source Data/Figure S6c/Figure S6c.png]

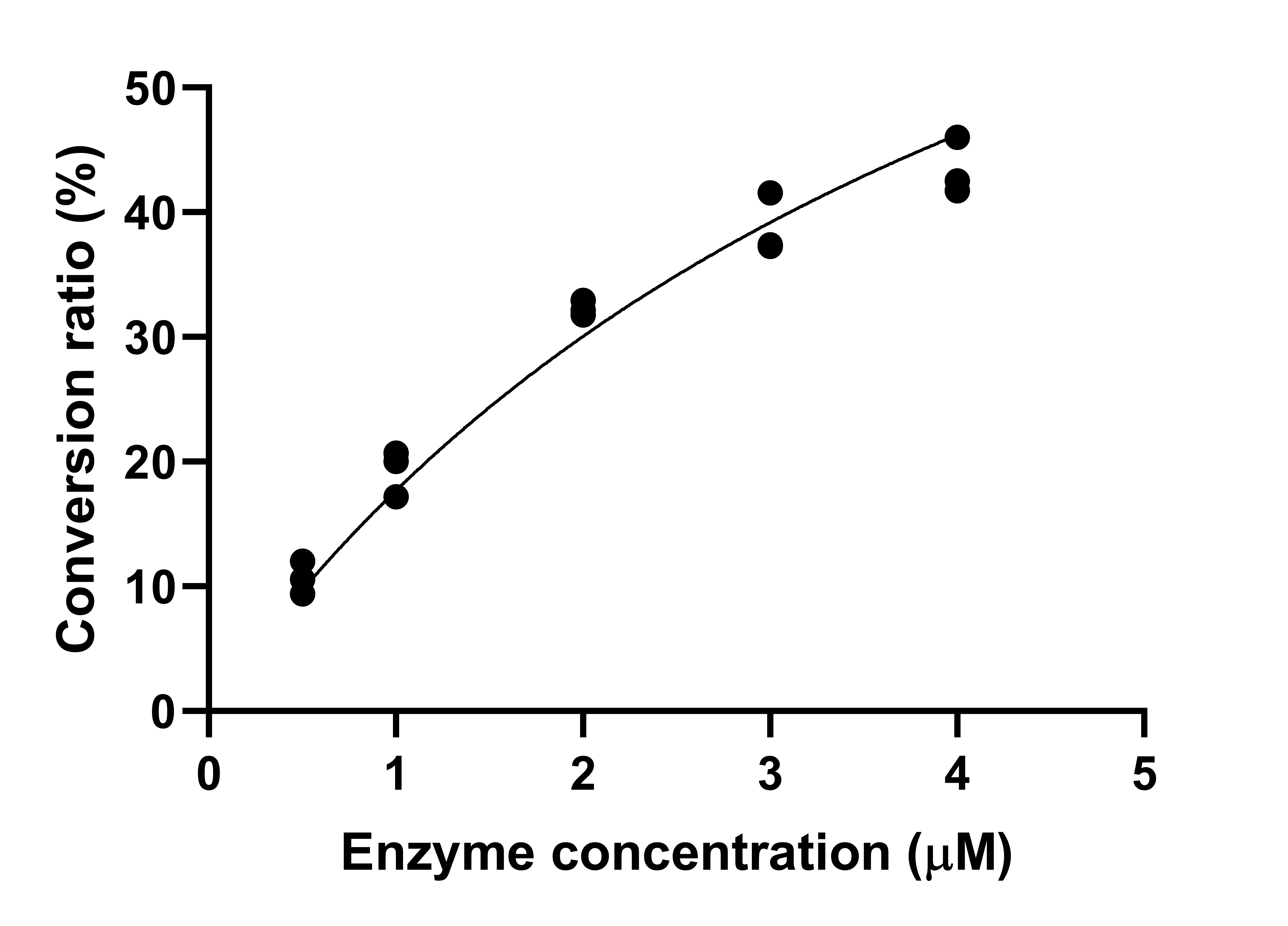

Supplement: Supplementary file 4 — Supplementary Data 1 [file 42003_2022_3257_MOESM4_ESM.zip › Source Data/Figure S6d/Figure S6d.png]

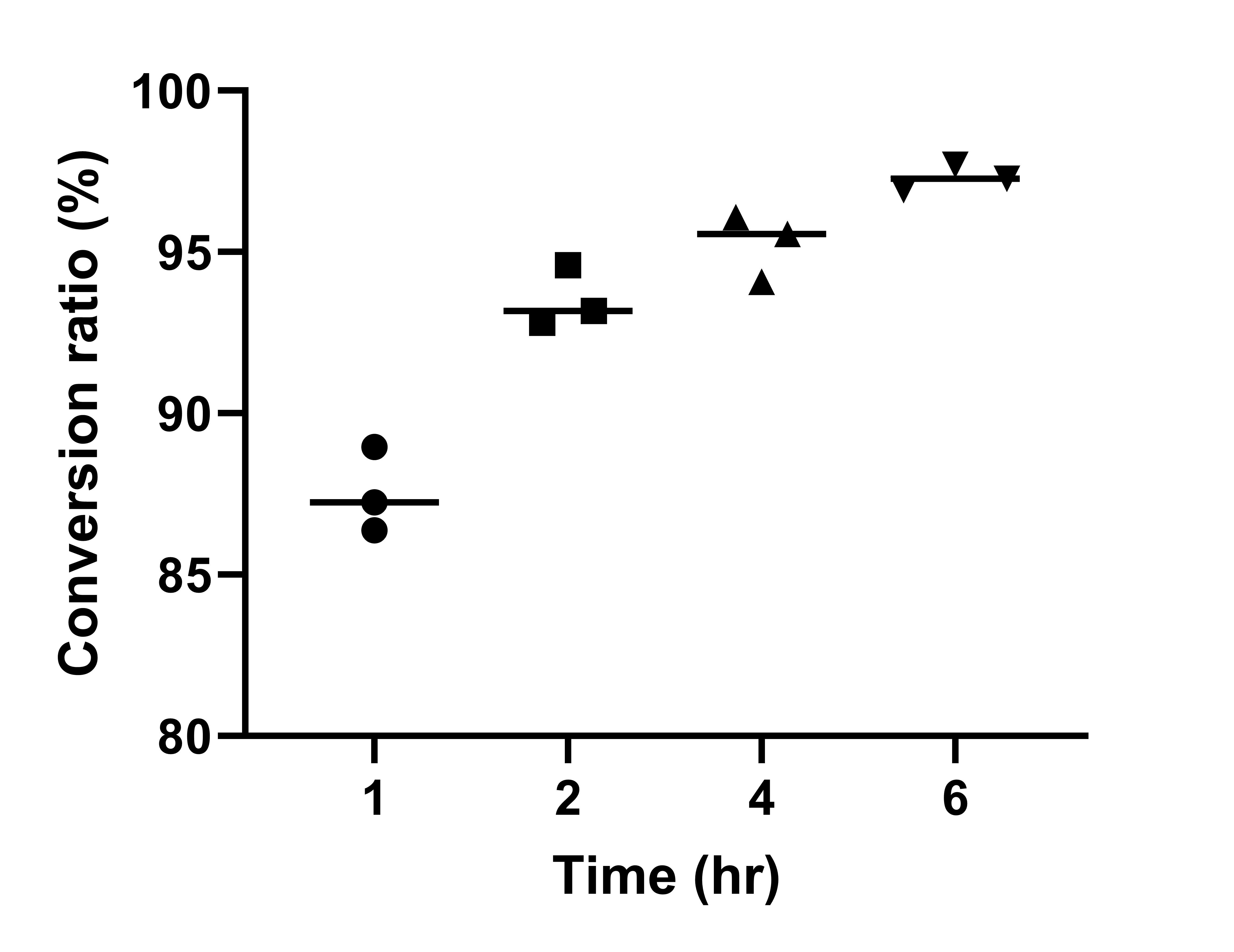

Supplement: Supplementary file 4 — Supplementary Data 1 [file 42003_2022_3257_MOESM4_ESM.zip › Source Data/Figure S6e/figure S6e.png]

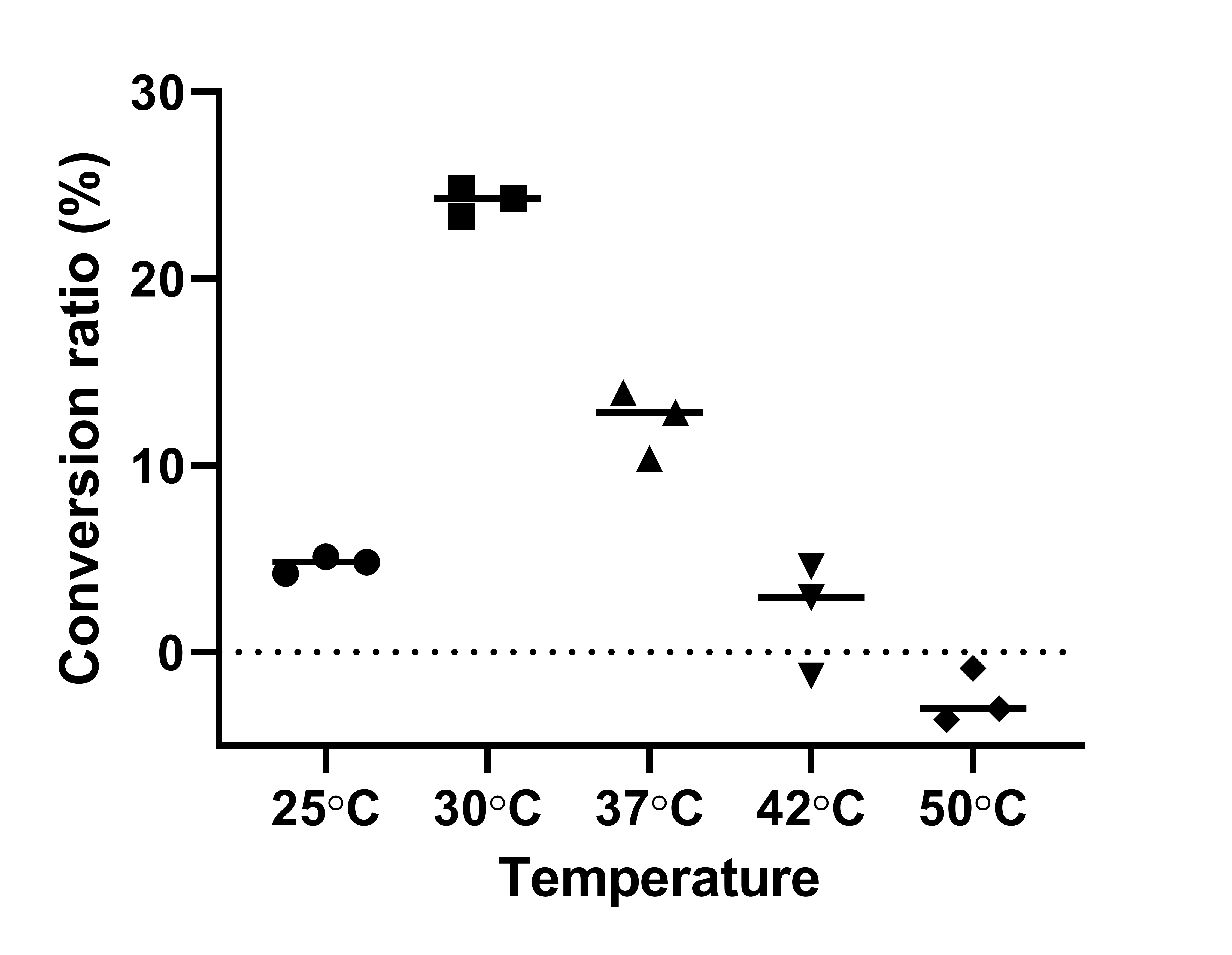

Supplement: Supplementary file 4 — Supplementary Data 1 [file 42003_2022_3257_MOESM4_ESM.zip › Source Data/Figure S8a-b/Figure S8a.png]

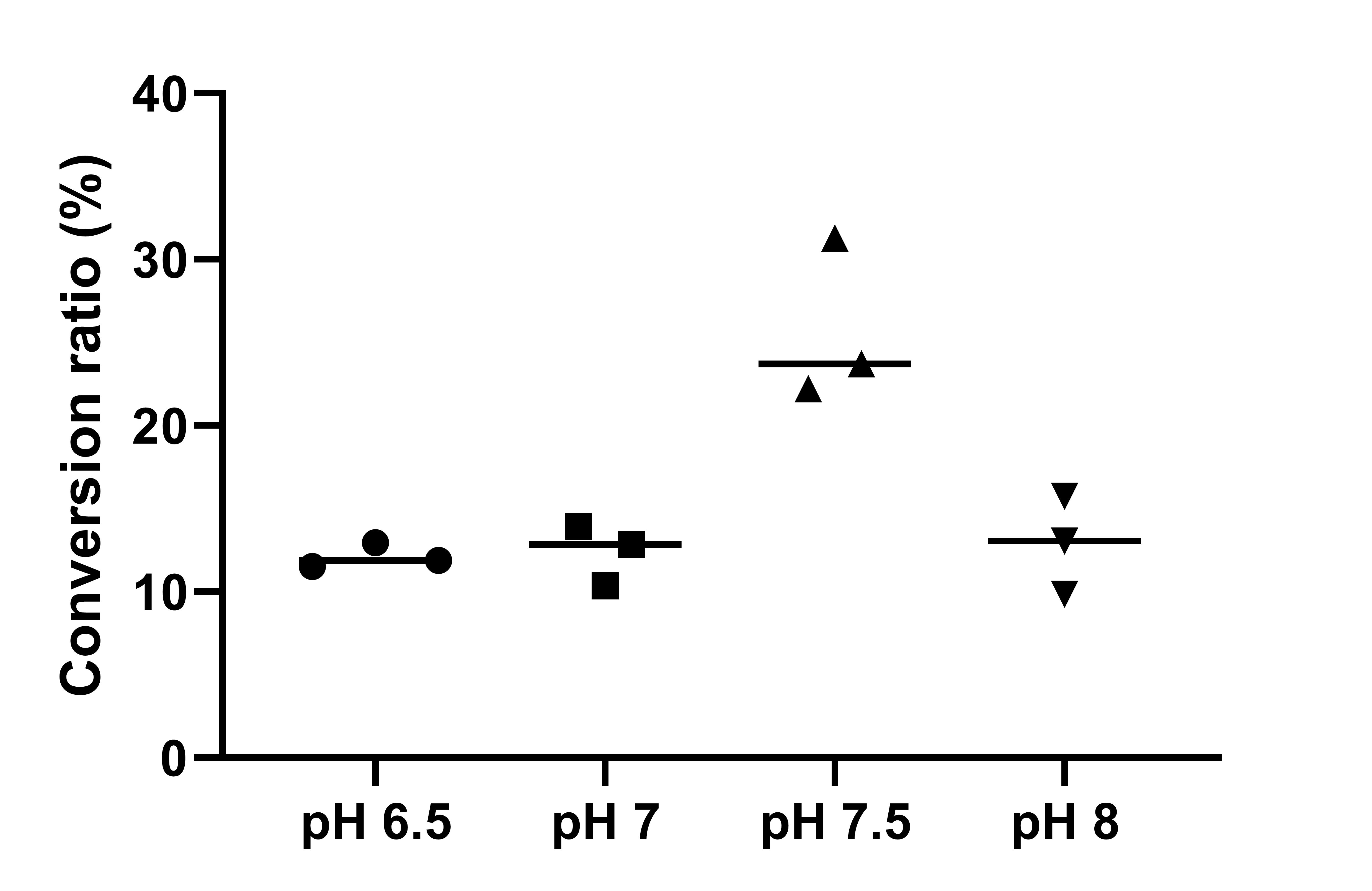

Supplement: Supplementary file 4 — Supplementary Data 1 [file 42003_2022_3257_MOESM4_ESM.zip › Source Data/Figure S8a-b/Figure S8b.png]

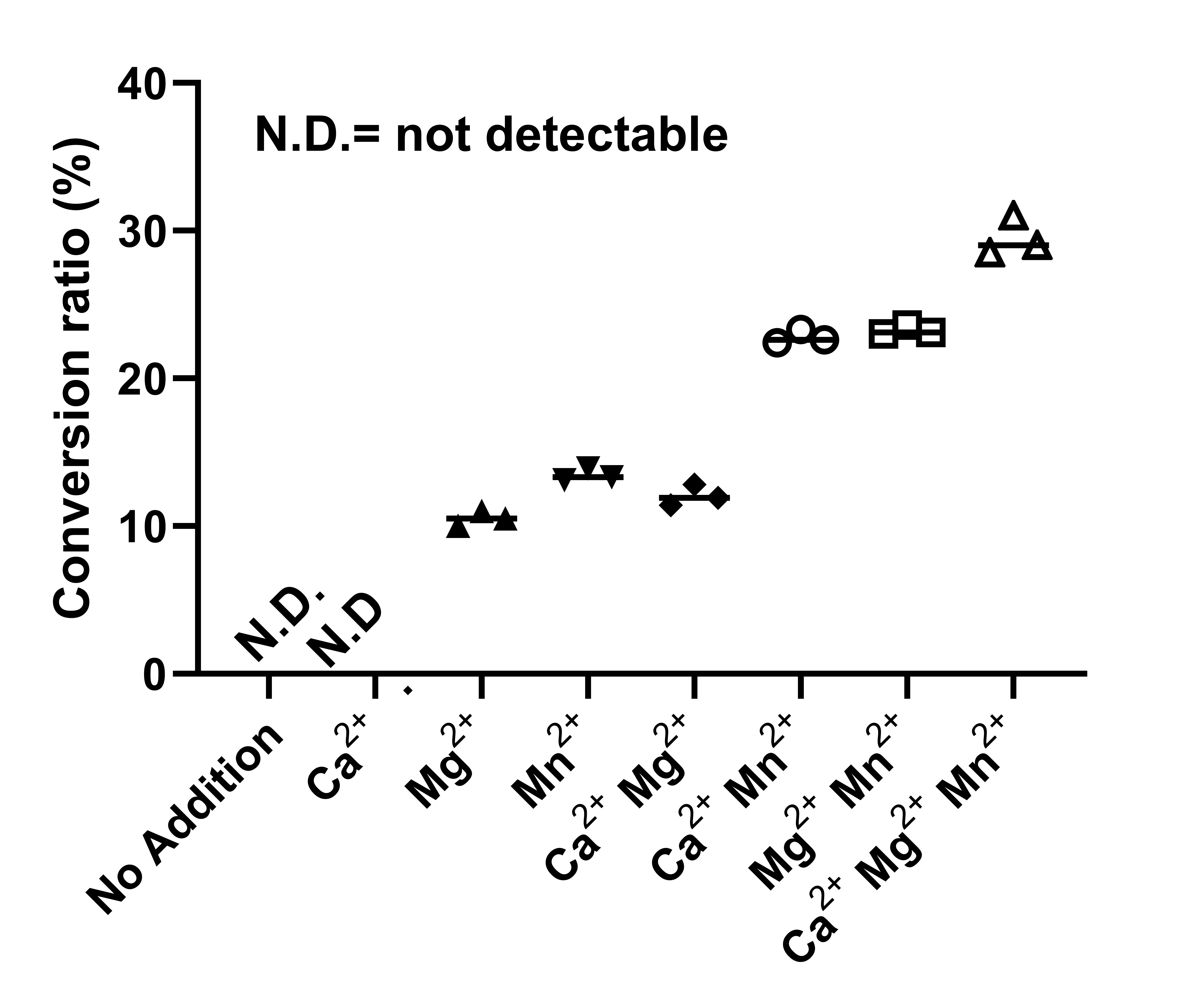

Supplement: Supplementary file 4 — Supplementary Data 1 [file 42003_2022_3257_MOESM4_ESM.zip › Source Data/Figure S8c/Figure S8c.png]

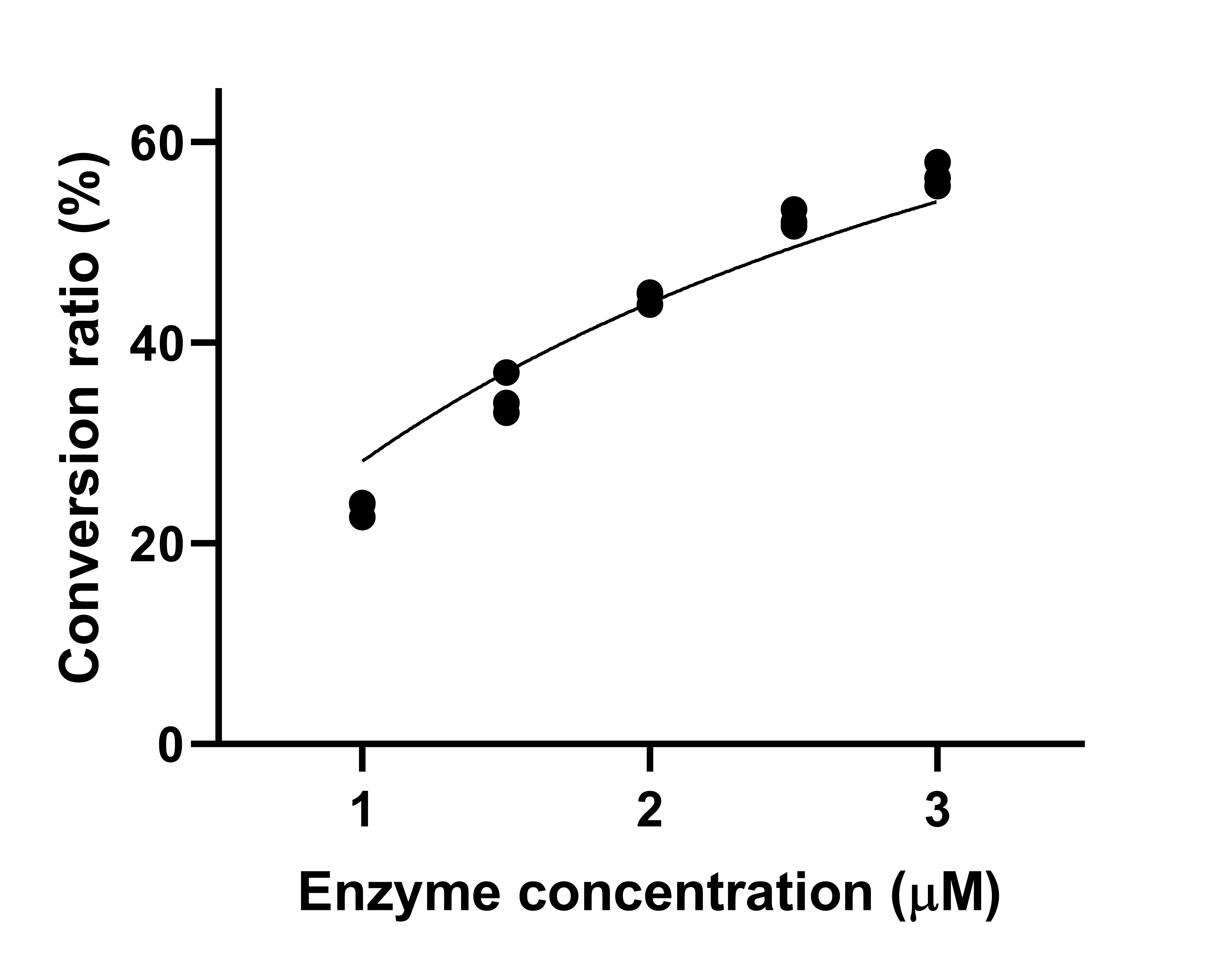

Supplement: Supplementary file 4 — Supplementary Data 1 [file 42003_2022_3257_MOESM4_ESM.zip › Source Data/Figure S8d/Figure S8d.png]

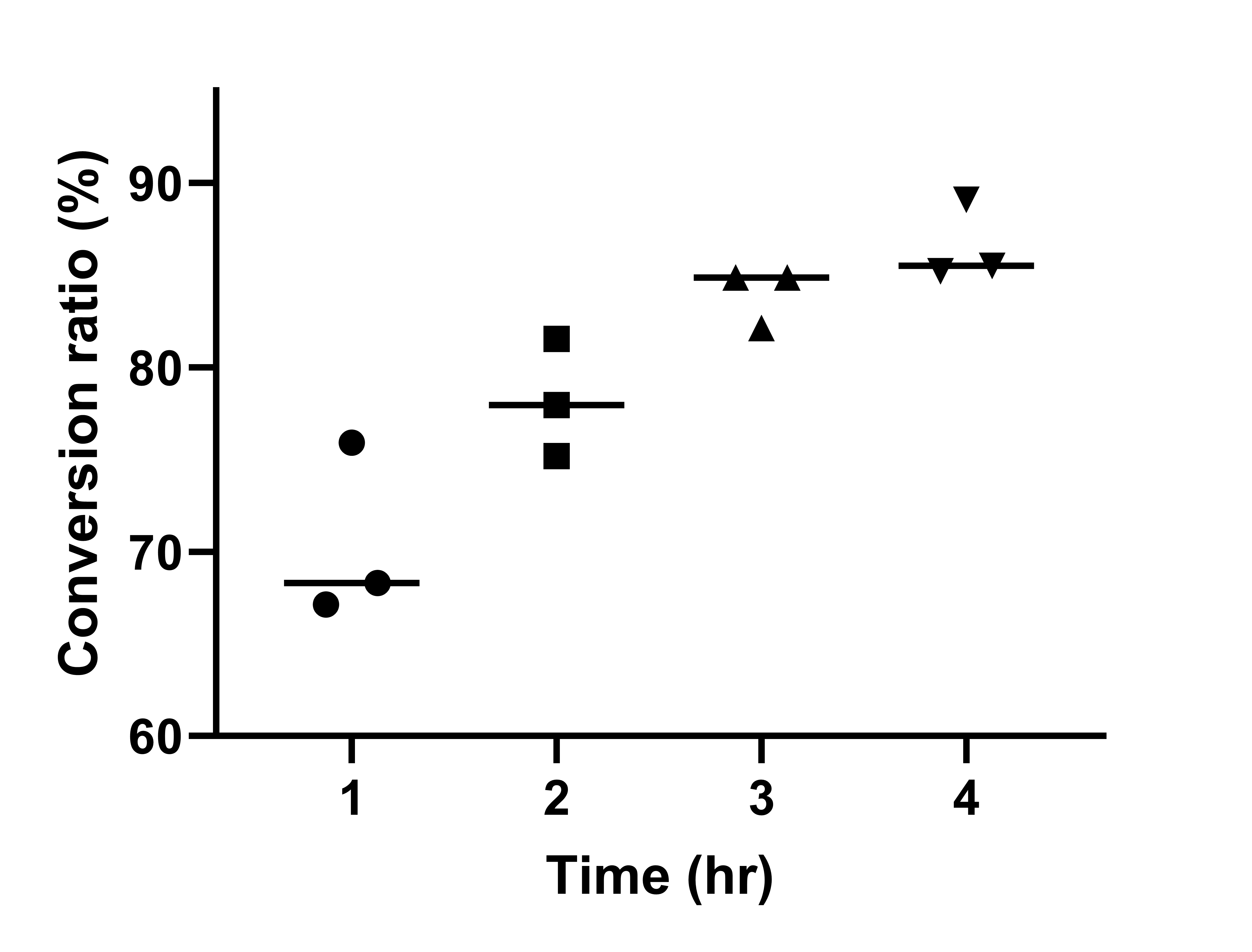

Supplement: Supplementary file 4 — Supplementary Data 1 [file 42003_2022_3257_MOESM4_ESM.zip › Source Data/Figure S8e/Figure S8e.png]

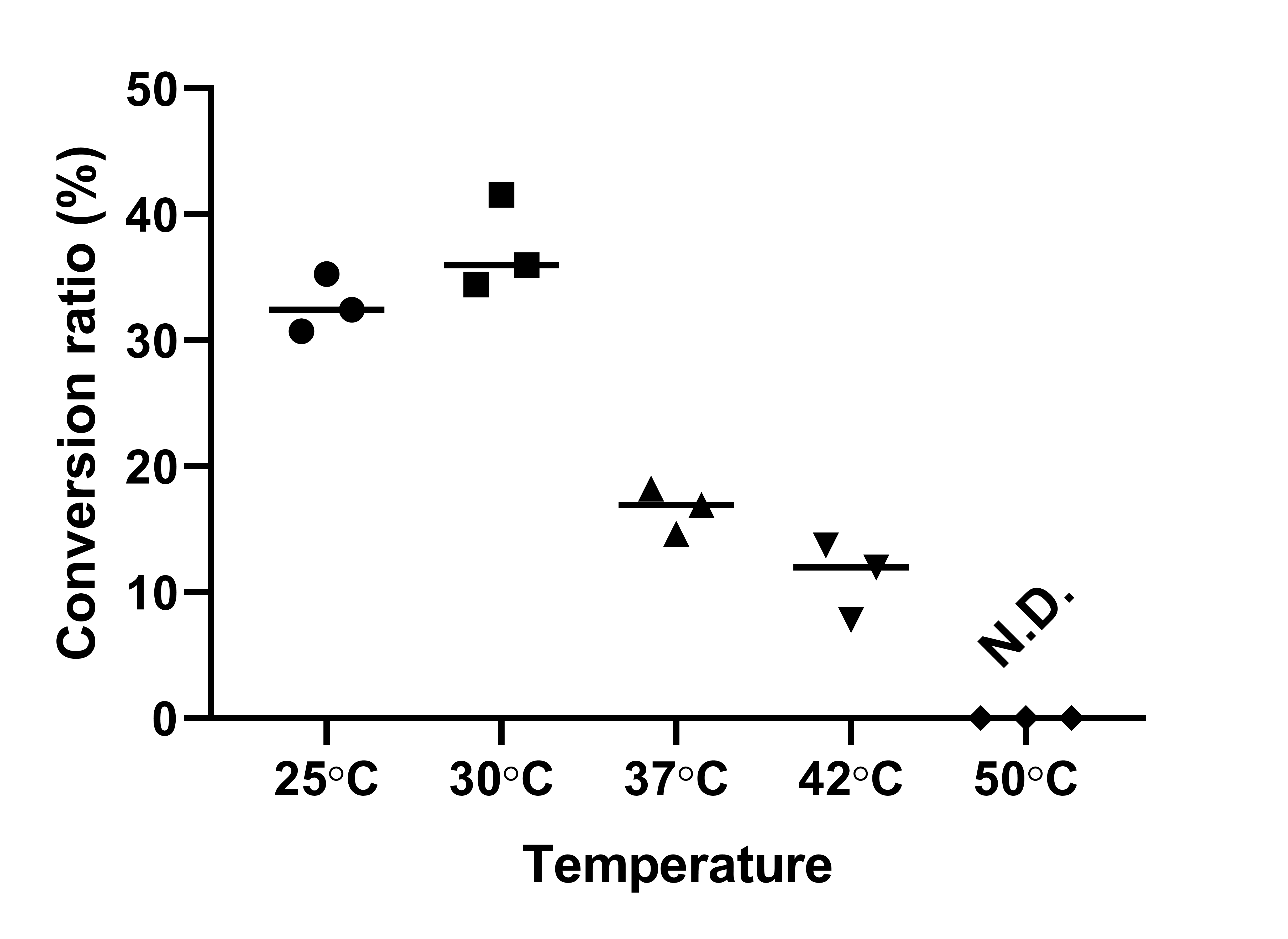

Supplement: Supplementary file 4 — Supplementary Data 1 [file 42003_2022_3257_MOESM4_ESM.zip › Source Data/Figure S9a-b/Figure S9a.png]

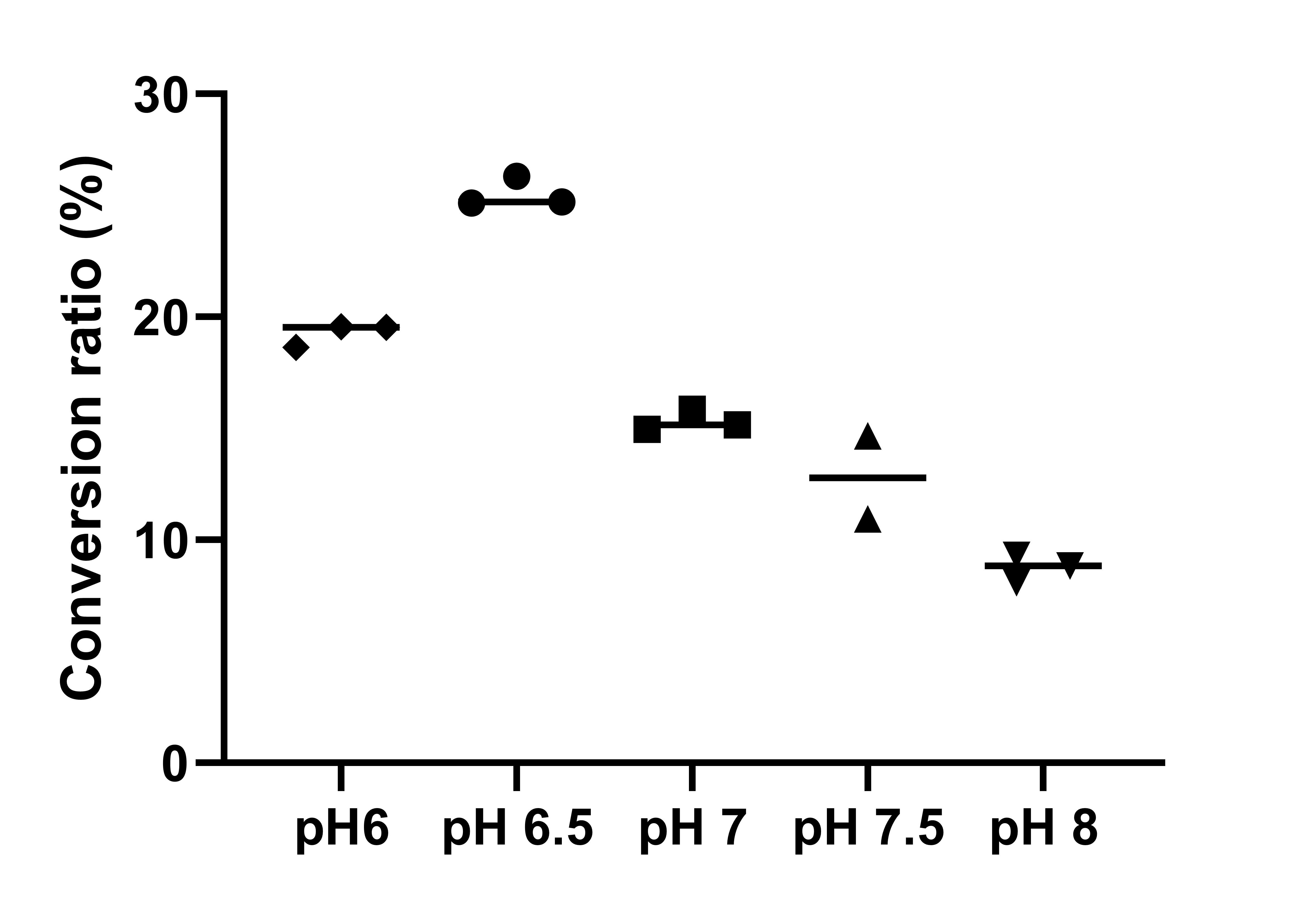

Supplement: Supplementary file 4 — Supplementary Data 1 [file 42003_2022_3257_MOESM4_ESM.zip › Source Data/Figure S9a-b/Figure S9b.png]

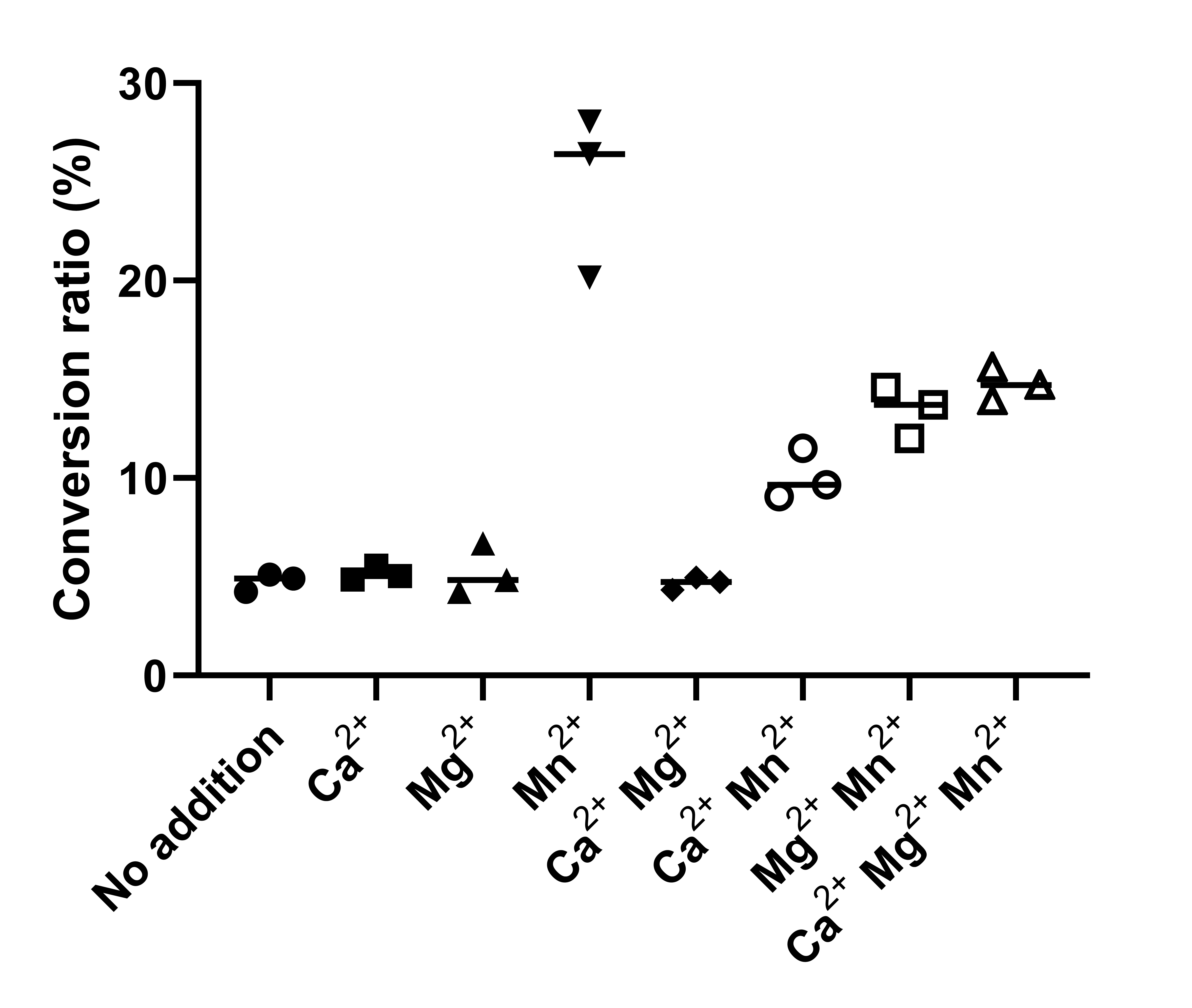

Supplement: Supplementary file 4 — Supplementary Data 1 [file 42003_2022_3257_MOESM4_ESM.zip › Source Data/Figure S9c/Figure S9c.png]

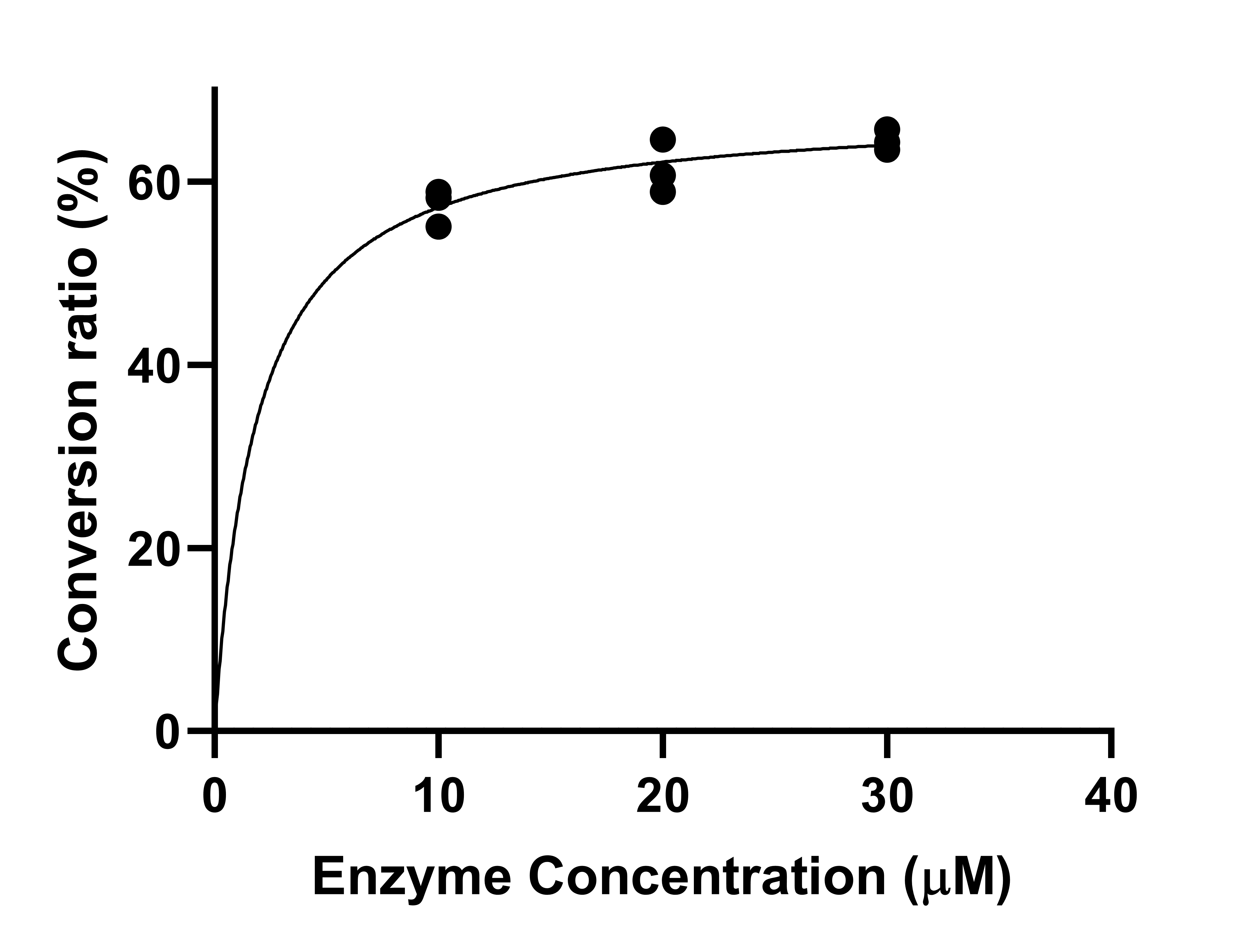

Supplement: Supplementary file 4 — Supplementary Data 1 [file 42003_2022_3257_MOESM4_ESM.zip › Source Data/Figure S9d/Figure S9d.png]

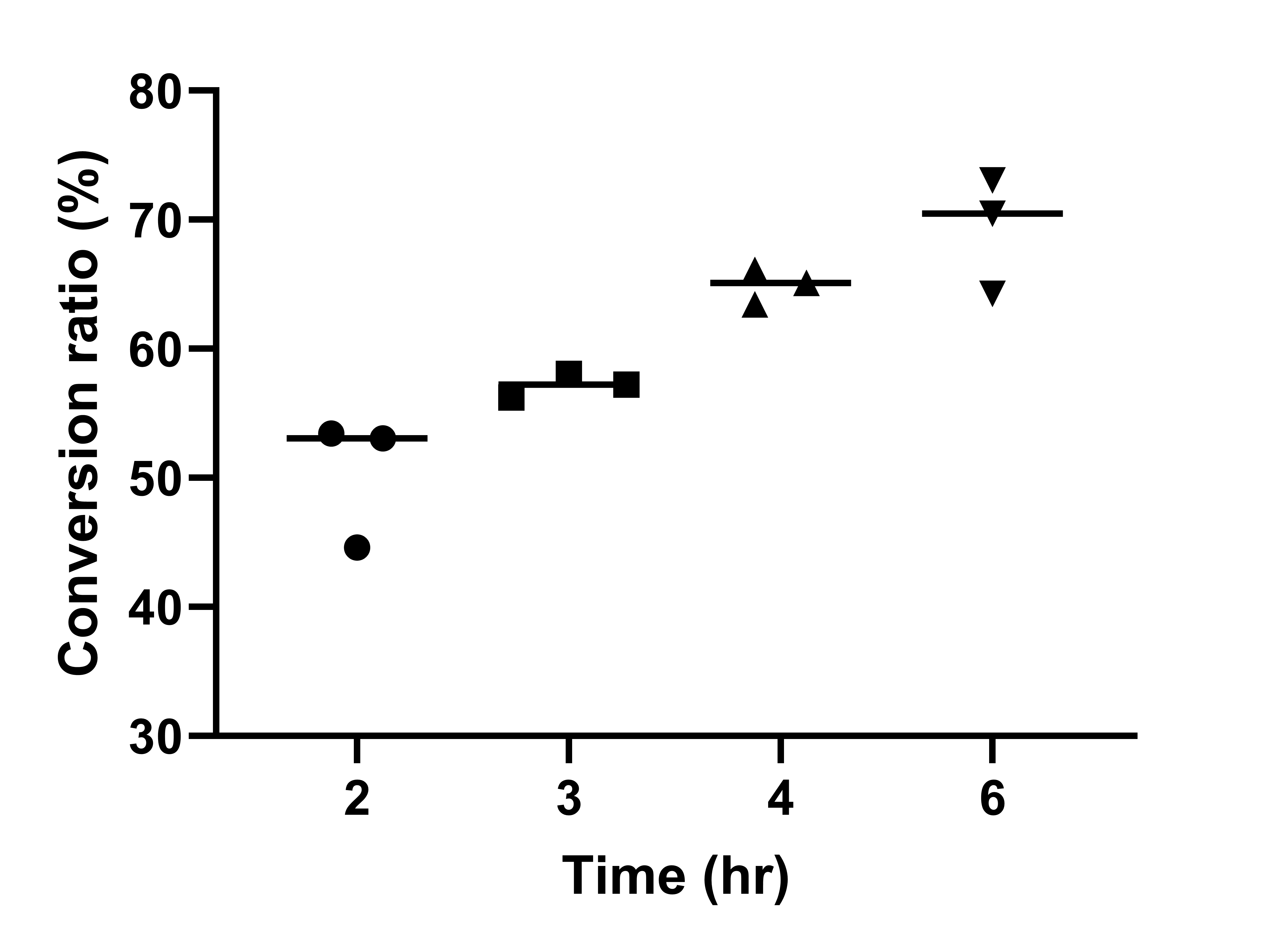

Supplement: Supplementary file 4 — Supplementary Data 1 [file 42003_2022_3257_MOESM4_ESM.zip › Source Data/Figure S9e-f/Figure S9e.png]

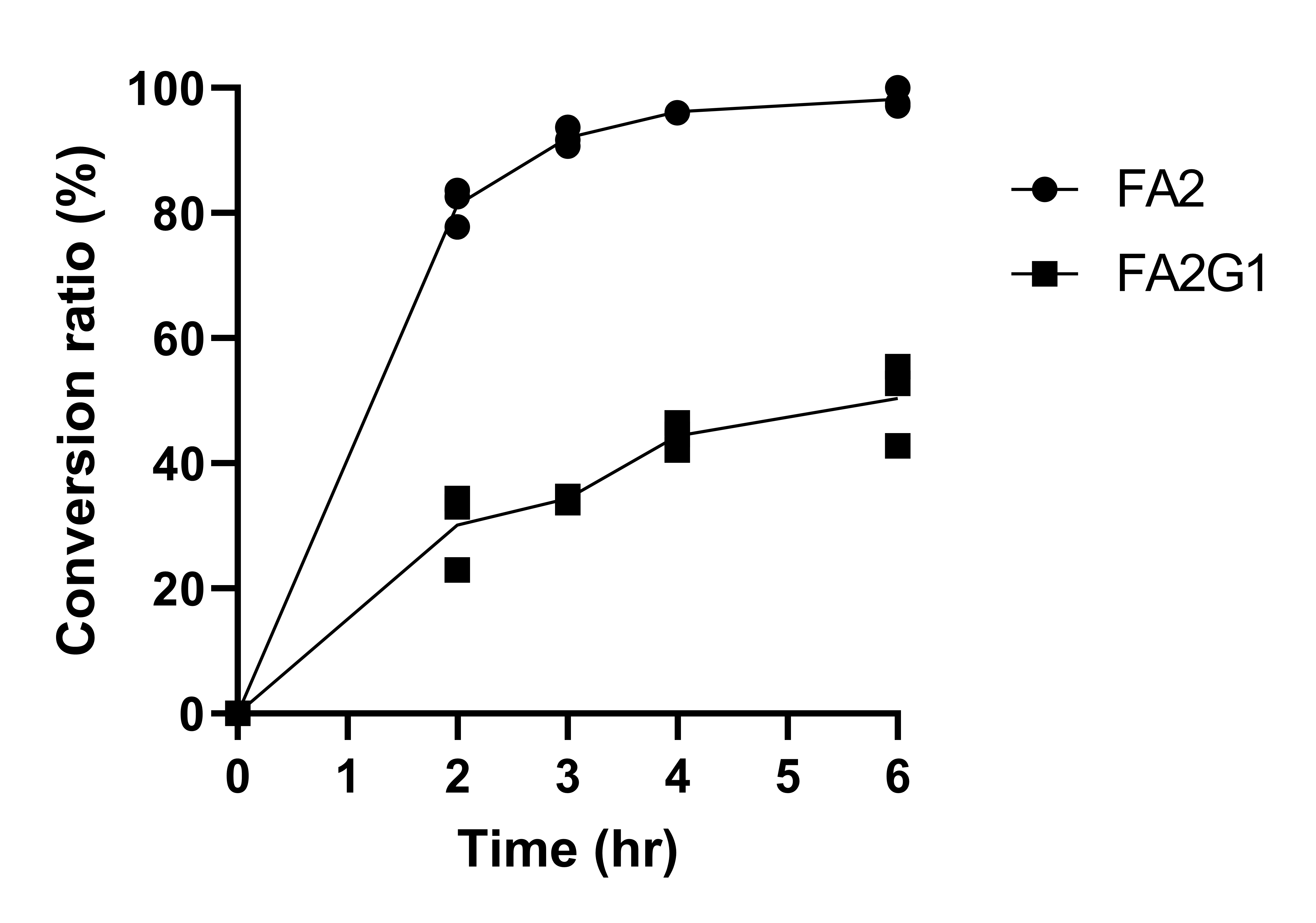

Supplement: Supplementary file 4 — Supplementary Data 1 [file 42003_2022_3257_MOESM4_ESM.zip › Source Data/Figure S9e-f/Figure S9f.png]
