# Supplementary material for: Successive remodeling of IgG glycans using a solid-phase enzymatic platform
Source: Commun Biol. 2022 Apr 7;5:328. doi: 10.1038/s42003-022-03257-4 (PMC8990068; doi:10.1038/s42003-022-03257-4)
Supplement: Supplementary file 5 — Reporting Summary [file 42003_2022_3257_MOESM5_ESM.pdf]

nature research

Corresponding author(s): Benjamin F. Mann

Last updated by author(s): Feb 4, 2022

Reporting Summary

Nature Research wishes to improve the reproducibility of the work that we publish. This form provides structure for consistency and transparency in reporting. For further information on Nature Research policies, see our Editorial Policies and the Editorial Policy Checklist.

Statistics

For all statistical analyses, confirm that the following items are present in the figure legend, table legend, main text, or Methods section.

n/a	Confirmed	
☐	☒	The exact sample size (n) for each experimental group/condition, given as a discrete number and unit of measurement
☐	☒	A statement on whether measurements were taken from distinct samples or whether the same sample was measured repeatedly
☒	☐	The statistical test(s) used AND whether they are one- or two-sided<br>*Only common tests should be described solely by name; describe more complex techniques in the Methods section.*
☒	☐	A description of all covariates tested
☒	☐	A description of any assumptions or corrections, such as tests of normality and adjustment for multiple comparisons
☐	☒	A full description of the statistical parameters including central tendency (e.g. means) or other basic estimates (e.g. regression coefficient) AND variation (e.g. standard deviation) or associated estimates of uncertainty (e.g. confidence intervals)
☒	☐	For null hypothesis testing, the test statistic (e.g. F, t, r) with confidence intervals, effect sizes, degrees of freedom and P value noted<br>*Give P values as exact values whenever suitable.*
☒	☐	For Bayesian analysis, information on the choice of priors and Markov chain Monte Carlo settings
☒	☐	For hierarchical and complex designs, identification of the appropriate level for tests and full reporting of outcomes
☒	☐	Estimates of effect sizes (e.g. Cohen's d, Pearson's r), indicating how they were calculated

Our web collection on statistics for biologists contains articles on many of the points above.

Software and code

Policy information about availability of computer code

Data collection	Glycan chromatograms and mass spectra were generated using Agilent MassHunter Workstation (Version B.09.00). Computational modeling data was generated using the Swiss-Model server and Pymol (Molecular Graphic System, Version 2.0). Protein physical chemistry property data was collected and calculated using the Unchained Labs UNcle software. EnVision Multimode Plate Reader 2105 and the associated analysis software (PerkinElmer) were used for AlphaLISA-based assays.
Data analysis	GraphPad Prism 8 was used for data analysis and plot/graph generation.

For manuscripts utilizing custom algorithms or software that are central to the research but not yet described in published literature, software must be made available to editors and reviewers. We strongly encourage code deposition in a community repository (e.g. GitHub). See the Nature Research guidelines for submitting code & software for further information.

Data

Policy information about availability of data

All manuscripts must include a data availability statement. This statement should provide the following information, where applicable:

- Accession codes, unique identifiers, or web links for publicly available datasets
- A list of figures that have associated raw data
- A description of any restrictions on data availability

All data are available upon request to the corresponding authors. Source data for plots is available in Supplementary Data 1

Field-specific reporting

Please select the one below that is the best fit for your research. If you are not sure, read the appropriate sections before making your selection.

☒ Life sciences ☐ Behavioural & social sciences ☐ Ecological, evolutionary & environmental sciences

For a reference copy of the document with all sections, see nature.com/documents/nr-reporting-summary-flat.pdf

Life sciences study design

All studies must disclose on these points even when the disclosure is negative.

Sample size	Triplicate repeats were performed with an exception of Fucosidase characterization studies due to the difficulties of material preparation.
Data exclusions	No data was excluded from the study.
Replication	Data were collected on different samples to ensure reproducibility. The experiments have been replicated on different days, with the use of different batches of materials. All attempts to replication were successful.
Randomization	Randomization is not relevant to this study as all data were generated by instrument-based measurements. In each experiment, identical conditions were used for all the samples to ensure fair comparison.
Blinding	Blinding is not relevant to this study as all data were generated by instrument-based measurements.

Reporting for specific materials, systems and methods

We require information from authors about some types of materials, experimental systems and methods used in many studies. Here, indicate whether each material, system or method listed is relevant to your study. If you are not sure if a list item applies to your research, read the appropriate section before selecting a response.

Materials & experimental systems

n/a	Involved in the study	
☐	☒	Antibodies
☒	☐	Eukaryotic cell lines
☒	☐	Palaeontology and archaeology
☒	☐	Animals and other organisms
☒	☐	Human research participants
☒	☐	Clinical data
☒	☐	Dual use research of concern

Methods

n/a	Involved in the study	
☒	☐	ChIP-seq
☒	☐	Flow cytometry
☒	☐	MRI-based neuroimaging

Antibodies

Antibodies used	Human serum IgG was used in this study. The material was provided by Sigma-Aldrich (Catalog number: I4506)
Validation	Information from the vendor: Purified Human IgG is isolated from pooled normal human serum by fractionation and ion-exchange chromatography. The product is supplied as an essentially salt-free (less than 1% sodium), lyophilized powder.
